# G3BP1 Succinylation at K413 is Critical for Cardiac Function by Modulating PI3K‐AKT‐mTOR Signal Axis

**DOI:** 10.1002/advs.202519856

**Published:** 2026-05-10

**Authors:** Yuan Zhang, Cancan Yao, Yan Chen, Ke Cai, Zhouping Lu, Boxuan Wu, Kun Yu, Yan Shi, Jianyuan Zhao, Xiangyu Zhou

**Affiliations:** ^1^ Shanghai Key Laboratory of Maternal Fetal Medicine Center for Assisted Reproduction Shanghai First Maternity and Infant Hospital Tongji University School of Medicine Shanghai China; ^2^ Obstetrics and Gynecology Hospital of Fudan University Shanghai Key Lab of Reproduction and Development Shanghai Key Lab of Female Reproductive Endocrine Related Diseases Shanghai China; ^3^ Institute For Developmental and Regenerative Cardiovascular Medicine MOE‐Shanghai Key Laboratory of Children's Environmental Health Xinhua Hospital Shanghai Jiao Tong University School of Medicine Shanghai China; ^4^ Suzhou Dushu Lake Hospital The Fourth Affiliated Hospital of Soochow University Soochow China

**Keywords:** dilated cardiomyopathy, G3BP1, heart failure, PI3K/mTOR, succinylation

## Abstract

G3BP1, GTPase activating protein (SH3 domain) binding protein 1, is a core component of stress granules. Homozygous null mutations in the G3bp1 gene result in embryonic lethality, underscoring its essential role in development. While various post‐translational modifications regulate G3BP1 activity, here we first report that G3BP1 undergoes succinylation (Suc) at Lys (K)411 in mouse hearts (corresponding to human K413). G3BP1‐Suc level was diminished in Myosin binding protein C3 (Mybpc3) knockout and transverse aortic constriction (TAC) operated mice, which developed heart failure (HF). Site‐directed mutagenesis confirmed that the K413R mutation compromised the overall Suc level of G3BP1 in vitro. Mice injected with AAV9‐G3BP1 (WT) developed typical phenotypes of dilated cardiomyopathy (DCM) and HF when compared to mice injected with AAV9‐Ctrl and ‐G3BP1 (K411R) mice, suggesting a possible loss of functional effect of de‐Suc at K411. Moreover, Homozygous knock‐in G3bp1 (K411R) mice exhibited compromised cardiac parameters compared to WT littermates. De novo G3BP1 mutation (p.E411G) from a DCM patient disrupts Suc at K413. Mechanistically, G3BP1 de‐Suc at K413 induced Rraga expression and impaired TSC1/2 and IDE binding, ultimately leading to excessive activation of the PI3K‐AKT‐mTOR signaling axis. We demonstrate a critical role for G3BP1 Suc at K413 in cardiac function by modulating the PI3K‐AKT‐mTOR pathway, providing new insights into the non‐canonical function of G3BP1 in cardiomyopathy and HF pathogenesis.

## Graphical abstract

Schematic illustrating the impact of G3BP1 succinylation at K413 on cardiac function. In the healthy human heart, G3BP1 succinylation maintains homeostatic mTOR signaling. In patients with dilated cardiomyopathy (DCM) and heart failure (HF), G3BP1 de‐succinylation induces RagA expression and disrupts the binding of the TSC1/2 complex, leading to the hyper‐activation of mTORC1 signaling.

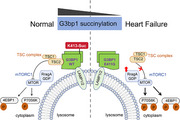

**Abbreviations**
ANP: atrial natriuretic peptide
Ctrl: control
DCM: dilated cardiomyopathy
DEPs: differentially expressed proteins
EF: ejection fraction
FS: fractional shortening
G3BP1: GTPase activating protein SH3 domain binding protein 1
GO: gene Ontology
HCM: hypertrophic cardiomyopathy
HF: heart failure
IDE: insulin‐degrading enzyme
KEGG: kyoto Encyclopedia of Genes and Genomes
KI: knock‐in
KO: knockout
LV: left ventricle
LVAW: left ventricular anterior wall thickness
LVPW: left ventricular posterior wall thickness
MS: mass spectrometer
mTOR: mammalian target of rapamycin
mybpc3: myosin binding protein C3
PASEF: parallel accumulation serial fragmentation
PTM: post‐translational modification
RNP: ribonucleoprotein
Rraga: ras‐related GTP binding A
SGs: stress granules
Suc: succinylation
TAC: transverse aortic constriction
TSC: tuberous sclerosis complex

## 1 Introduction

Dilated cardiomyopathy (DCM) is a prevalent etiology of non‐ischemic heart failure (HF) [1, 2], exhibiting a higher incidence in males compared to females [3]. Despite advances in HF therapeutics, DCM‐associated mortality remains substantial [4]. The pathogenesis of cardiomyopathy encompasses both genetic and non‐genetic mechanisms [5, 6]. Succinylation (Suc), a post‐translational modification (PTM) with demonstrated involvement in cardiac function [7, 8, 9], is mediated by the transfer of a succinyl moiety from succinyl‐CoA to lysine residues [10]. Studies have reported decreased Suc levels in myofibrillar and mitochondrial proteins in patients with ischemic cardiomyopathy and chronic HF [11, 12]. Nevertheless, the precise role of succinylation changes in the development of HF and cardiomyopathy remains largely elusive.

Here, we reported that Ras GTPase‐activating protein‐binding protein 1 (G3BP1) undergoes Suc at K413 that is critical for maintaining cardiac function. G3BP1 is a core component of stress granules (SGs), dynamic assemblies of RNA and protein that form in response to cellular stress and disassemble upon stress resolution [13]. Increased cellular free RNA concentration can trigger RNA‐dependent liquid–liquid phase separation (LLPS) [13, 14]. Two recent studies reported that G3BP1 promotes intermolecular RNA‐RNA interactions and reinforces the stress translation program by prioritizing the translation of its resident mRNAs [15, 16]. G3BPs function not only as core components of SGs but also execute diverse biological roles. G3bp1 was initially identified as an element of the Ras signaling pathway, interacting with the Ras GTPase activating protein via its SH3 domain [17]. There is mounting evidence that G3BP1 serves as a crucial regulator in multiple signaling pathways such as TSC‐mTORC1 [18], Cul3^SPOP^‐AR [19], Rac1‐PAK1 [20], NF‐κB [21], and STAT3 [22]. Furthermore, G3BP1 enhances cGAS binding to DNA by facilitating the formation of larger cGAS complexes [23]. Especially, G3BPs localized to the cytoplasmic surface of lysosomes anchor the tuberous sclerosis complex (TSC), leading to suppression of mTOR complex 1 (mTORC1) activation [18]. These findings suggest that G3BPs function both as structural components of SGs and as key regulators within the lysosomal TSC–mTORC1 signaling axis.

Various epigenetics and post‐translational modifications (PTMs) regulate G3BP1 activity and contribute to its diverse biological functions. For example, arginine demethylation in the RGG domain of G3BP1 promotes stress granule assembly and attenuates ferroptosis in diabetic myocardial ischemia‐ reperfusion injury [24, 25]. Phosphorylation‐mediated conformational changes in G3BP1 can reduce the driving force of phase separation, thus regulating SGs assembly via condensation [26, 27]. Ubiquitination of G3BP1, mediated by its interaction with the endoplasmic reticulum‐associated protein, promotes SG disassembly [28, 29]. Acetylation of G3BP1 at lysine (K) residue K376 modulates RNA binding and SG dynamics [30].

Homozygous null mutations of the G3bp1 gene in 129/Sv mice result in embryonic lethality [31], highlighting the essential role of G3bp1 in development. G3BP1 deficiency in neurons leads to abnormal synaptic plasticity and impaired calcium homeostasis through generating viable homozygous knockout (KO) mice with a mixed genetic background [32]. This finding is further supported by the identification of a significant enrichment of de novo G3BP1/2 variants in patients with neurodevelopmental disorders [33]. Independent genetic study identified eight highly penetrant, unique, or de novo G3BP1 variants in patients with unexplained multiple systemic disorders [18]. These findings suggest G3BP1 variants generally contribute to human disease.

Although currently there is no direct genetic evidence to support the correlation between G3bp1 and HF, dysregulated ribonucleoprotein (RNP) granules have been shown to promote DCM [34]. Stress granules are a prominent type of RNP granule, with G3BP1 serving as a central node in this network [35]. Rbm20^R636S^ and Rbm20^S637G^ mutations can promote the assembly of RNP granules and lead to severe DCM, reinforcing the concept that RBM20 cardiomyopathy is an RNP‐based granule disease [36, 37]. Furthermore, some evidence from animal models suggests that G3bp1 expression increases during cardiac hypertrophy [38], and knockdown of endogenous G3bp1 leads to downregulation of genes involved in calcium handling and cardiac muscle contraction [39], suggesting a possible contribution to cardiomyopathy development.

In this study, we present the first evidence that succinylation of G3BP1 at K413. Using two transgenic models, we demonstrate that G3BP1 succinylation at this site is crucial for cardiac function. De‐succinylation of G3BP1 at K413 promotes Raga expression and impairs its binding to insulin‐degrading enzyme (IDE) and TSC1/2, ultimately leading to hyper‐activation of the insulin‐mediated PI3K/AKT/mTOR signaling pathway. Moreover, a human G3BP1 de novo mutation (p.E411G) may induce dilated cardiomyopathy (DCM) by abrogating succinylation at K413.

## 2 Results

### 2.1 Suc Levels on G3BP1 were Decreased in Mybpc3 KO and TAC‐Operated Mice

**FIGURE 1:**
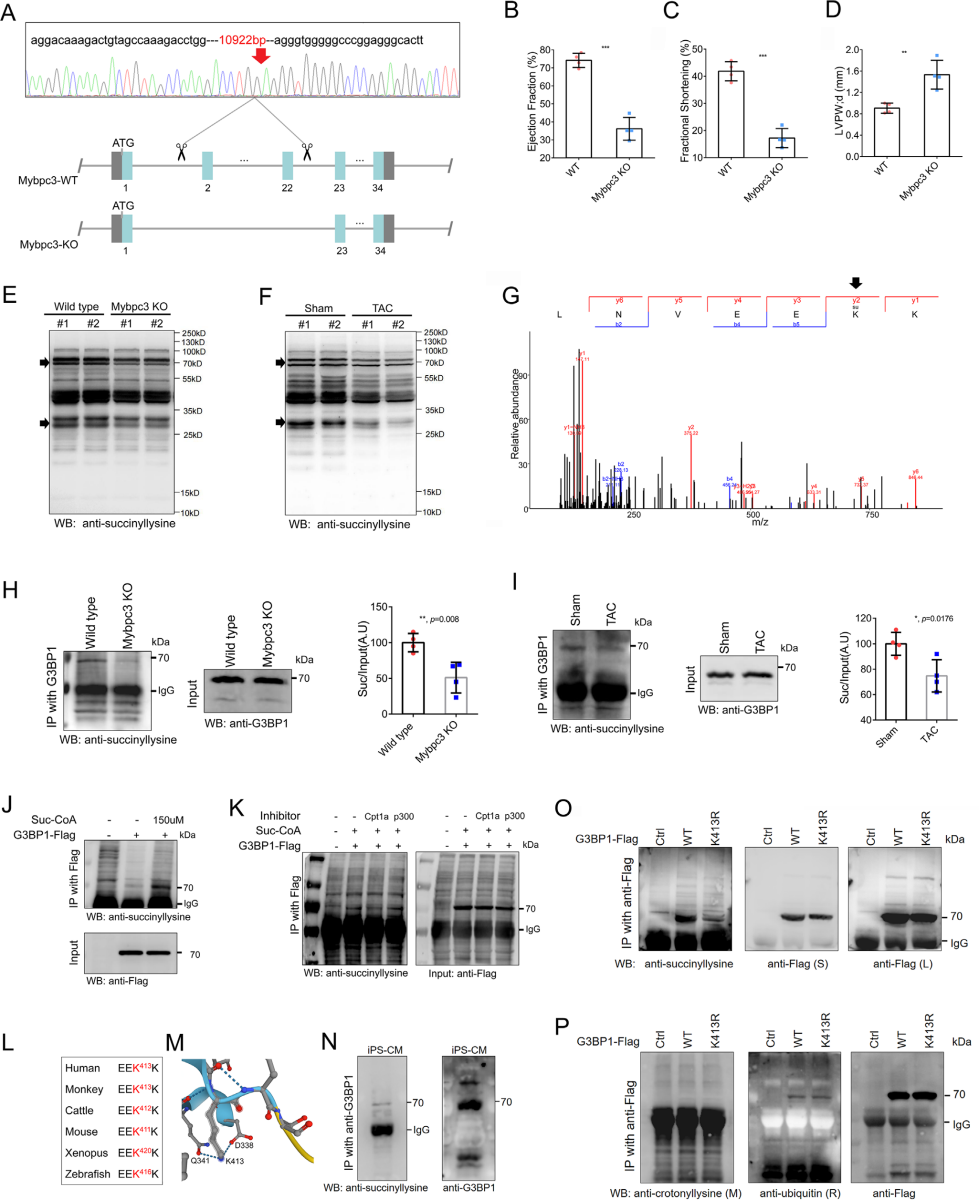
Decreased Suc levels on G3bp1 in two mice models developed cardiomyopathy and HF. (A) Schematic representation of Mybpc3 knockout (KO) mice generation using the CRISPR/Cas9 system. Sanger sequencing confirmed a 10922‐bp deletion spanning from exon 2 to exon 22. (B–D) Echocardiography data from Mybpc3 KO and wild‐type (WT) mice, showing core cardiac parameters including ejection fraction (EF), fractional shortening (FS), and left ventricular posterior wall thickness (LVPW). (*n* = 4 mice per group, each mouse assayed in triplicate). The data are presented as the mean ± SEM. Two‐tailed Student's *t*‐test: ns, not significant, ^**^ *p* < 0.01, ^***^ *p* < 0.001. s, systolic; d, diastolic. (E,F) Immunoblot analysis of anti‐succinyllysine in heart tissue of Mybpc3 KO (E) and TAC‐operated (F) mice using corresponding pan‐antibodies by western blots. WT and sham‐operated mice served as the control (Ctrl) group, respectively. Representative images from three independent experiments were displayed. (G) The MS/MS spectrum of the K411 Suc modified site in G3bp1. (H,I) Suc levels on G3bp1 in heart tissues of Mybpc3 KO and TAC‐operated mice, when compared with WT and sham mice, respectively, through IP assay with anti‐G3bp1 endogenous antibody. Quantification data of Suc levels when normalized against G3BP1 input. The data are presented as the mean ± SEM, *n* = 4. Two‐tailed Student's *t*‐test: ^*^ *p* < 0.05, ^**^ *p* < 0.01. (J) Suc‐CoA treatment with 150 µm efficiently enhances the Suc levels of Flag‐tagged recombinant G3BP1 in 293T cells. (K) G3BP1 Suc levels is reduced by CPT1A inhibitor treatment with 5 µm for 12 h whereas p300 inhibitor treatment with 5 µm has no obvious effect on Suc signals when referring to G3bp1 inputs in 293T cells. (L) Sequence alignment of Suc sites in K413 of hG3BP1 in different species. (M) AlphaFold protein structure prediction showing the possible altered net charge of lysine Suc at G3BP1 K413. (N) Suc signals on G3BP1 were validated in human cardiomyocytes derived from hiPSCs. A representative image from three independent experiments is displayed. (O,P) Replacing lysine with arginine at K413 sites led to decreased G3BP1 Suc levels (upper), but had no effect on G3BP1 ubiquitination (bottom) level in 293T cells. Cells were transfected with Flag‐tagged WT and point mutants of G3BP1, and cell lysates were then immunoprecipitated with an anti‐Flag antibody, followed by immunoblotting with a pan antibody as indicated. Cells transfected with the empty vector served as Ctrl group. S, short exposure, L, long exposure. R, rabbit; M, mouse. For each western blot, representative images from three independent experiments were displayed.

Succinylation levels are reported to be lower in patients and mice models with ischemic cardiomyopathy and chronic HF [11, 12]. Here, we initially construct two classical hypertrophic cardiomyopathy (HCM) and heart failure (HF) animal models, Mybpc3 (Myosin binding protein C3) knockout (Figure 1A) and transverse aortic constriction (TAC) (Figure S1A). Mybpc3 is one of the most common pathogenic genes for HCM, reportedly carried by about 40% of HCM patients, often with heart failure with preserved ejection fraction [40]. However, homozygous mice with mutations in the Mybpc3 gene have a progressive DCM with prominent histopathology of myocyte hypertrophy [41]. Echocardiography analysis validated decreased ejection fractions (EF) (Figure 1B; Figure S1B) and fractional shortening (FS) (Figure 1C), but increased LV posterior wall (LVPW) thicknesses in Mybpc3 KO mice (Figure 1D) and TAC‐operated mice before we evaluate the Suc changes in heart tissue. By performing antibody screening, Suc levels on non‐histone proteins were significantly reduced in the hearts of Mybpc3‐KO (Figure 1E; Figure S1C), and TAC‐operated mice (Figure 1F) when compared with wild‐type (WT) control or Sham group at the age of 12 weeks, respectively. Notably, two mainly decreased WB bands were located at near 70 and 30 KDa approximately. We next perform in‐gel digestion combined with LC‐MS/MS to precisely determine Suc changes in two mouse models, and a total of 17 significantly decreased succinylation sites were identified. Notably, G3bp1 K411^suc^ (fold change = 0.248) was the most significantly down‐regulated Suc site in Mybpc3 KO mice (Figure 1G), indicating a possible site‐specific effect of G3bp1 K411^suc^ on biological processes in the heart.

### 2.2 Validation of G3bp1 Suc at K411 In Vitro and In Vivo

We next validated the changes of G3BP1 succinylation in two models that displayed HF. Immunoprecipitation (IP) with an anti‐G3BP1 antibody demonstrated significantly reduced G3BP1‐Suc levels in heart tissues from both Mybpc3‐KO (Figure 1H) and TAC‐operated mice compared to their respective controls (Figure 1I). We also demonstrated that treatment with Suc‐CoA enhanced Suc levels of Flag‐tagged recombinant G3BP1 in 293T cells (Figure 1J). Since CPT1A and P300 have been reported to possess lysine succinyltransferase activity [42, 43], we examined their roles in G3BP1 succinylation. The CPT1A inhibitor, but not the P300 inhibitors, reduced G3BP1 Suc levels in 293T cells in vitro (Figure 1K).

Sequence alignments indicated that human G3bp1 K413 (correspond to mouse K411) was highly conserved across species, including Zebrafish and Xenopus (Figure 1L). According to the AlphaFold Protein Structure Database, changes in the net charge of G3bp1 K413 induced by Suc may destroy hydrogen bonds with D338 and Q341, which can potentially exert a direct influence on the overall protein conformation (Figure 1M). We then validated the Suc signals on G3BP1 in human cardiomyocytes derived from hiPSCs (Figure 1N). G3BP1 Suc levels were detected in RAA tissues from tetralogy of fallot (TOF) patients (Figure S2A); however, the absence of matched healthy controls prevented us from evaluating changes in G3BP1‐Suc levels.

To mimic de‐Suc, we performed site‐directed mutagenesis to mutate K413 in human G3BP1 to R413, which removed the Suc site. Potential changes in PTMs were then evaluated by performing immunoprecipitation assays with HEK‐293T cells overexpressing recombinant Flag‐tagged G3BP1 proteins. To obtain an overview of G3BP1 PTMs, we studied acetylation, Suc, ubiquitination, and crotonylation via western blot analysis using corresponding pan antibodies. We detected ubiquitination, acetylation, and Suc in recombinant G3BP1 proteins, but not crotonylation. However, mutating K to arginine (R) at the G3bp1‐Suc site to generate the K413R markedly reduced Suc levels in vitro (Figure 1O), whereas acetylation and ubiquitination were not significantly different between WT G3BP1 and the K413R mutant (Figure 1P; Figure S2B). Our findings support a potential role for G3BP1‐Suc in the development of cardiomyopathy and HF in mice models.

### 2.3 Reduced G3BP1 Succinylation is Driven by the Decreased of the Succinyl Donor Pool in Mybpc3 KO and TAC‐Operated Mice

To investigate the potential mechanisms underlying the reduction of G3BP1 succinylation in Mybpc3 KO mouse hearts, we performed global 4D label‐free quantitative (LFQ) proteomic analysis on heart tissues from Mybpc3 KO mice (*n* = 4) and age‐matched WT littermates (*n* = 4) at 3 months of age (Figure S3A). Principal component analysis (PCA) effectively separated the 8 heart samples into two distinct groups (Figure S3B). This analysis identified 42 403 peptides corresponding to 5322 proteins (Figure S3C). We identified 63 significantly up‐regulated and 104 down‐regulated proteins (fold‐change > 1.5; *p* < 0.05) in Mybpc3 KO mice (Figure S3D). As expected, Mybpc3 was the most down‐regulated protein among the 5322 quantified proteins (fold‐change = 0.0029) (Figure S3E). Notably, the majority of down‐regulated proteins were localized to the mitochondria, and GO enrichment analysis further confirmed the significant enrichment of mitochondria‐related terms (Figure S3F). Interestingly, the protein levels of the succinyltransferase CPT1A, as well as the de‐succinylases SIRT5 and HDAC1, remained unchanged in Mybpc3 KO mouse hearts (Figure S4). Therefore, we hypothesized that the decreased succinylation levels were caused by a reduction in succinyl‐CoA, the primary donor for protein succinylation, in Mybpc3 KO hearts. Consistently, the protein levels of key mitochondrial enzymes directly involved in succinyl‐CoA production, such as SUCLA2 and SDHA, were down‐regulated, while OGDH remained unchanged in Mybpc3 KO hearts (Figure S4). We subsequently employed LC‐MS‐based targeted analysis to evaluate succinyl‐CoA levels in the plasma and heart tissues of both Mybpc3 KO and TAC‐operated mice (Figure S5A,B), which revealed a significant reduction of succinyl‐CoA in heart samples (Figure S5C–F). These findings suggest that a reduced availability of the succinyl donor pool occurs in pathological cardiac states in Mybpc3 KO and TAC‐operated mice.

### 2.4 Suc at K411 is a Critical G3bp1 Activity Regulatory Site in Mice Hearts Through AAV9 Injection

**FIGURE 2:**
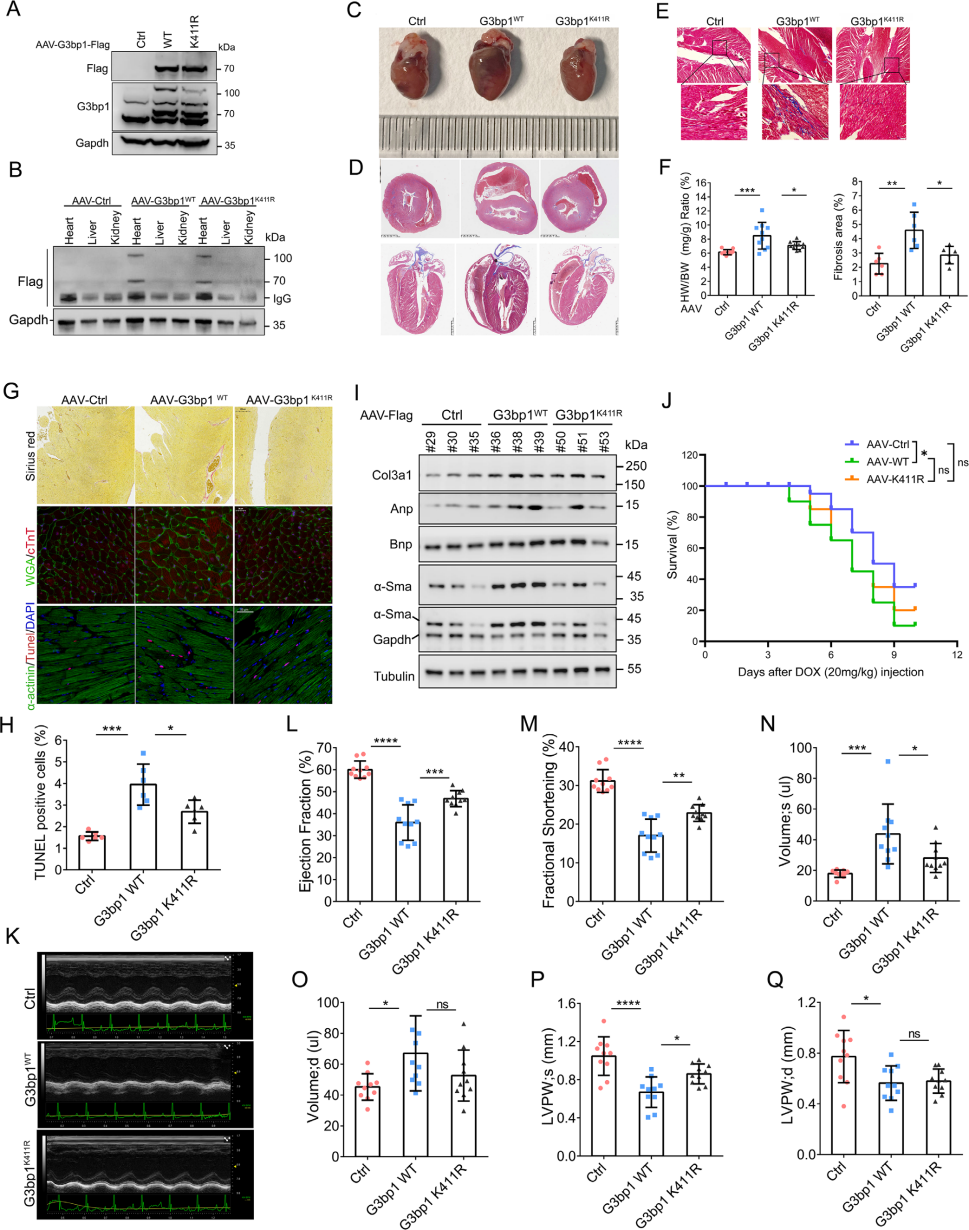
Suc at K411 is a critical regulatory site for G3BP1 activity in mice hearts. (A,B) The abundance (A) and specificity (B) of AAV9‐mediated G3bp1 transgene expression were evaluated using western blot analysis of mouse hearts 4 weeks after injection. For each western blot, representative images from three independent experiments were displayed. (C,D) Morphological observations (C) and Masson‐stained sections (vertical and horizontal) (D) of mouse heart injected with AAV9‐Ctrl, ‐G3bp1^WT^, and ‐G3bp1^K411R^. Scale bar, 1.25 mm. (E) Representative image of myocardial fibrosis was observed in mice injected with AAV‐G3bp1^WT^ when compared with AAV‐G3bp1^K411R^ and Ctrl. Fibrosis areas are boxed to show the enlarged region. Scale bar, 200 µm (upper), 50 µm (bottom). (F) The ratio of heart weight to body weight (HW/BW) (*n* = 10 per group) and quantitative analysis of fibrotic scar from Masson's trichrome staining (*n* = 6 per group). Mice injected with AAV‐empty vector served as Ctrl group. The data are presented as the mean ± SEM; one‐way ANOVA test, ^*^ *p* < 0.05, ^**^ *p* < 0.01,^***^ *p* < 0.001. (G) Representative images of Sirius red, WGA, and TUNEL staining in mice heart injected with AAV‐Ctrl, ‐G3bp1 WT, and K411R. Cardiomyocyte specificity was confirmed by co‐staining with cTnT and α‐actinin, respectively. Scale bar, 200, 20, and 50 µm from up to bottom. (H) Quantification of TUNEL‐positive cardiomyocytes in (G). Data are presented as the mean ± SEM; one‐way ANOVA test, *n* = 6. ^*^ *p* < 0.05, ^***^ *p* < 0.001. (I) Immunoblot analysis of classical biomarkers for cardiac hypertrophy and fibrosis, including Col3a1, Anp, Bnp, and α‐Sma, in these mice models. Gapdh and Tubulin served as loading controls. A representative image from three independent experiments was displayed. (J) Kaplan–Meier survival curves for AAV‐Ctrl and ‐G3bp1^WT^ and ‐G3bp1^K411R^ mice subjected to DOX treatment. (*n* = 20 per group; log‐rank test ^*^p = 0.0311, ns, not significant). (K) Representative images of echocardiography in mice injected with AAV‐Ctrl, ‐G3bp1 WT, and ‐K411R. (L–Q) Core parameters of echocardiography analysis, including ejection fraction, fractional shortening, volume, and LVPW. s, systolic; d, diastolic. Mice injected with AAV‐empty vector served as Ctrl group. The data are presented as the mean ± SEM; each mouse was assayed in triplicate, one‐way ANOVA test, *n* = 10. ns, not significant, ^*^ *p* < 0.05, ^**^ *p* < 0.01, ^***^ *p* < 0.001, ^****^ *p* < 0.0001.

To clarify the site‐specific effects of G3bp1 K411^Suc^ in vivo, we constructed WT and K411R (correspond to human K413) G3bp1‐eGFP‐AAV9 expression vectors. The K411R G3bp1‐eGFP‐AAV9 vector was generated to mimic the de‐succinylated state of overexpressed G3bp1. Control (Ctrl)‐, G3bp1^WT^‐, and G3bp1^K411R^‐AAV9 viruses were injected intravenously into male 6‐week‐old C57BL6 mice (*n* = 10/group). The abundance and specificity of AAV9‐mediated transgene expression were evaluated using western blot analysis of mouse hearts 4 weeks after injection (Figure 2A,B). Recombinant Flag‐tagged G3bp1 protein was specifically expressed in heart tissues but not in the liver or kidney tissues. At 12 weeks of age, the gross heart volume in the G3bp1^WT^‐AAV9 group was larger than that in the G3bp1^K411R^‐AAV9 and Ctrl‐AAV9 groups, as determined through anatomical sampling (Figure 2C), immunohistochemical staining (Figure 2D,E), and heart weight to body weight (HW/BW) ratio (Figure 2F). Haematoxylin and eosin‐stained heart‐tissue sections did not show obvious differences between the groups, but Masson's trichrome, Sirius Red, wheat germ agglutinin (WGA), and cardiac troponin T (cTnT) staining provided evidence of cardiomyocyte hypertrophy and myocardial fibrosis in mice injected with G3bp1^WT^‐AAV9 (Figure 2F–H). TUNEL staining indicated enhanced myocardial DNA damage (Figure 2G,H; Figure S6), whereas Ki67 staining showed no significant changes for cardiomyocyte proliferation in G3bp1‐WT AAV9 injected mice when compared with Ctrl‐AAV and G3bp1‐K411R mice (Figure S7). Western blot analysis further demonstrated that classical biomarkers of cardiac hypertrophy and fibrosis, including α‐SMA, Col3a1, and Anp were up‐regulated in the G3bp1^WT^‐AAV9 group (Figure 2I). Subsequently, mice transduced with AAV‐Ctrl and AAV‐G3BP1 were subjected to a Doxorubicin (DOX)‐induced cardiac injury model (20 mg/kg, intraperitoneal injection). Survival rates were monitored over a 10 day period (*n* = 20 per group). Our results revealed that mice receiving AAV‐G3BP1^WT^ exhibited significantly higher mortality following DOX challenge, whereas AAV‐G3bp1^K411R^ showed no significant alteration in survival rate when compared to AAV‐Ctrl‐treated mice (Figure 2J). Echocardiography analysis (Figure 2K) indicated that the ejection fraction (Figure 2L) and shortening fraction (Figure 2M) were significantly lower in mice administered G3bp1^WT^‐AAV9 than in mice administered Ctrl‐AAV9 or G3bp1^K411R^‐AAV9 at 12 weeks of age, whereas the volume (systolic) (Figure 2N,O) was significantly higher, reflecting typical symptoms of cardiac dysfunction in mice administered G3bp1^WT^‐AAV9. We did not observe significant changes in the LVAW (systolic) and LVAW (diastolic), but LVPW (systolic) (Figure 2P) and LVPW (diastolic) (Figure 2Q) were decreased in mice administered G3bp1^WT^‐AAV9. These findings demonstrate that G3bp1 succinylation at K411 is a critical regulatory site for cardiac function, and de‐succinylation(Suc) at K411 would induce a loss of function effect for G3bp1 activity.

### 2.5 G3bp1 Point Mutation Knock‐in (p.K411R) Mice Displayed Reduced Cardiac Function

**FIGURE 3:**
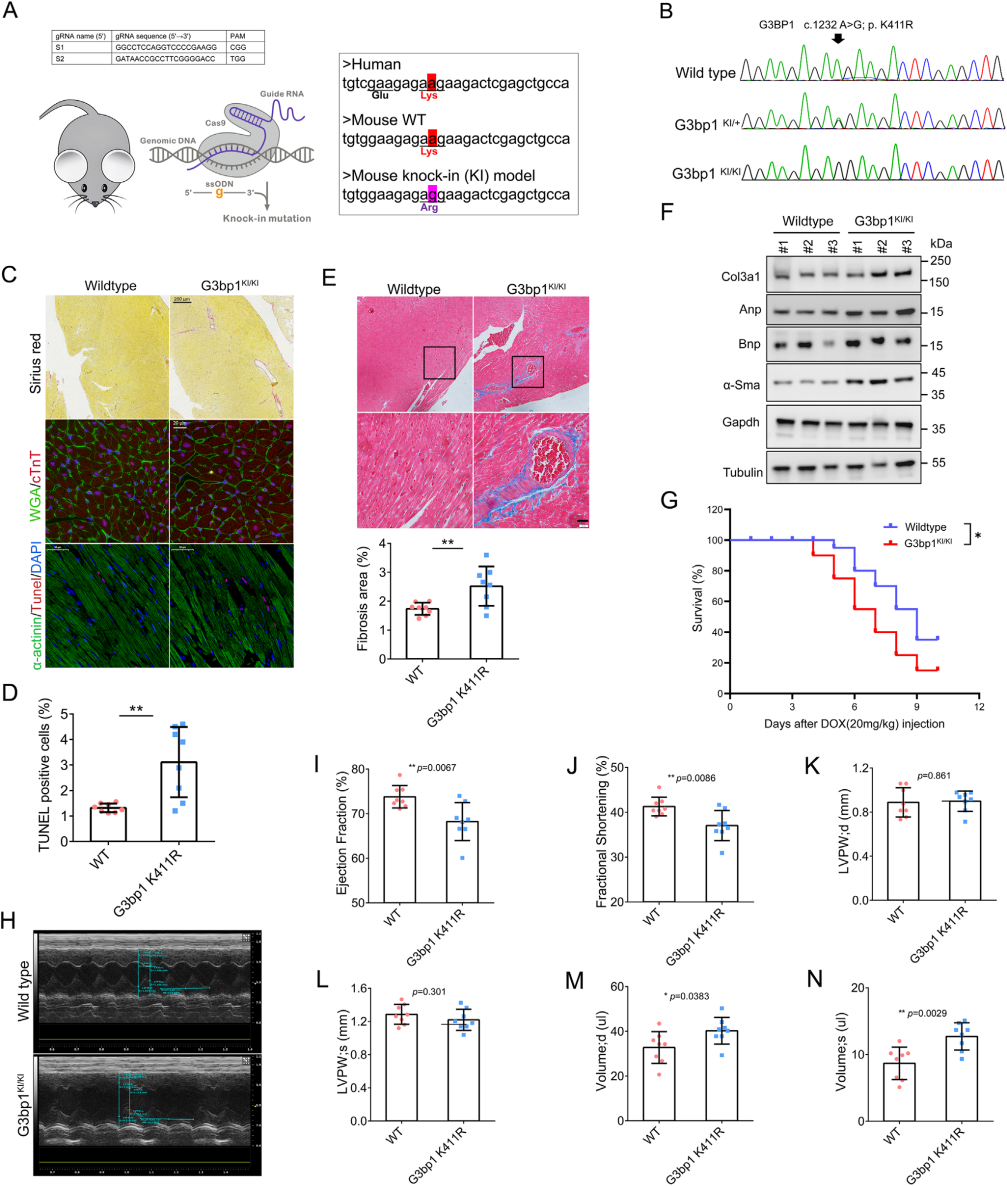
G3bp1 KI (p.E411G) mice displayed reduced cardiac function. (A) Schematic of G3bp1 A‐to‐G single base KI mouse model generated using CRISPR/Cas9 sgRNA. (B) The mutation in G3bp1 (c.1232A>G, mouse K411 corresponds to human K413) in the KI mouse was confirmed by Sanger sequencing. The c.1232 site is indicated by a black arrow. (C) Representative images of Sirius red, WGA, and TUNEL staining in WT and G3bp1 ^KI/KI^ mice hearts. Cardiomyocyte specificity was confirmed by co‐staining with cTnT and α‐actinin, respectively. Scale bar, 200, 20, and 50 µm from up to bottom. (D) Quantification of TUNEL‐positive cardiomyocytes in (C). Data are presented as the mean ± SEM; two‐tailed Student's *t* test, *n* = 8. ^**^ *p* < 0.01. (E) Masson's trichrome staining identified fibrosis in G3bp1^KI/KI^ mice heart when compared with WT and quantitative analysis of fibrotic scar (*n* = 8 per group). The data are presented as the mean ± SEM; two‐tailed Student's *t*‐test, ^**^ *p* < 0.01. (F) Immunoblot analysis of classical biomarkers for cardiac hypertrophy and fibrosis, including Col3a1, Anp, Bnp, and α‐Sma in WT and G3bp1 ^KI/KI^ mice models. Gpadh and Tubulin served as loading controls. A representative image from three independent experiments was displayed. (G) Kaplan–Meier survival curves for WT and G3bp1^KI/KI^ mice subjected to DOX treatment. (*n* = 20 per group; log‐rank test ^*^p = 0.0392). (H‐N) Echocardiography analysis revealed a significant decrease in LV fractional shortening and ejection fractions and enlarged LV volume in G3bp1^KI/KI^ mice when compared to those in WT littermates at 16 weeks of age. WT littermates served as Ctrl group. Each mouse was assayed in triplicate. s, systolic; d, diastolic. The data are presented as the mean ± SEM; two‐tailed Student's *t* test, *n* = 8. ^*^ *p* < 0.05, ^**^ *p* < 0.01.

To further investigate the importance of G3bp1 K411 Suc for cardiac function, we next generated a knock‐in (KI) mouse model carrying a point mutation at this site (mouse c.1232A>G, p.K411R, corresponding to human c.1236A>G, p.K413R) using CRISPR/Cas9 technology (Figure 3A). The homozygous genotype was then confirmed by PCR‐based Sanger sequencing (Figure 3B). Homozygous null mutations of the G3bp1 gene in mice result in embryonic lethality. However, the percentages of wild‐type, heterozygous, and homozygous G3bp1 (K411R) mice were close to the expected Mendelian rate on E17.5 days. Anatomical analysis of G3bp1^KI/KI^ mice did not reveal typical phenotypes of congenital heart disease at E17.5 days. Sirius Red, wheat germ agglutinin (WGA), and cardiac troponin T (cTnT), and Masson's trichrome staining also provided evidence of cardiomyocyte hypertrophy and myocardial fibrosis in G3bp1 ^KI/KI^ mice (Figure 3C–E). Consistent with the findings in the AAV models, TUNEL and Ki67 staining exhibited similar alterations in G3bp1^KI/KI^ mice when compared with WT mice (Figure 3C,D; Figures S6 and S7). Western blot analysis further confirmed the elevation of cardiac hypertrophy and fibrosis biomarkers (Figure 3F). G3bp1^KI/KI^ mice exhibited significantly higher mortality following DOX challenge when compared to wild‐type (WT) mice (Figure 3G). Echocardiography analysis (Figure 3H) further revealed a moderate but significant decrease in LV fractional shortening and ejection fractions, and enlarged LV volume in G3bp1^KI/KI^ mice when compared to those in WT littermates at 16 weeks of age, as aforementioned observation in mice injected with AAV‐G3bp1 WT (Figure 3I–N). These findings suggested de‐suc of G3bp1 p.K411R significantly compromised cardiac function in mice.

### 2.6 G3BP1 Interacts With Insulin‐Degrading Enzyme (IDE) in Human Heart

**FIGURE 4:**
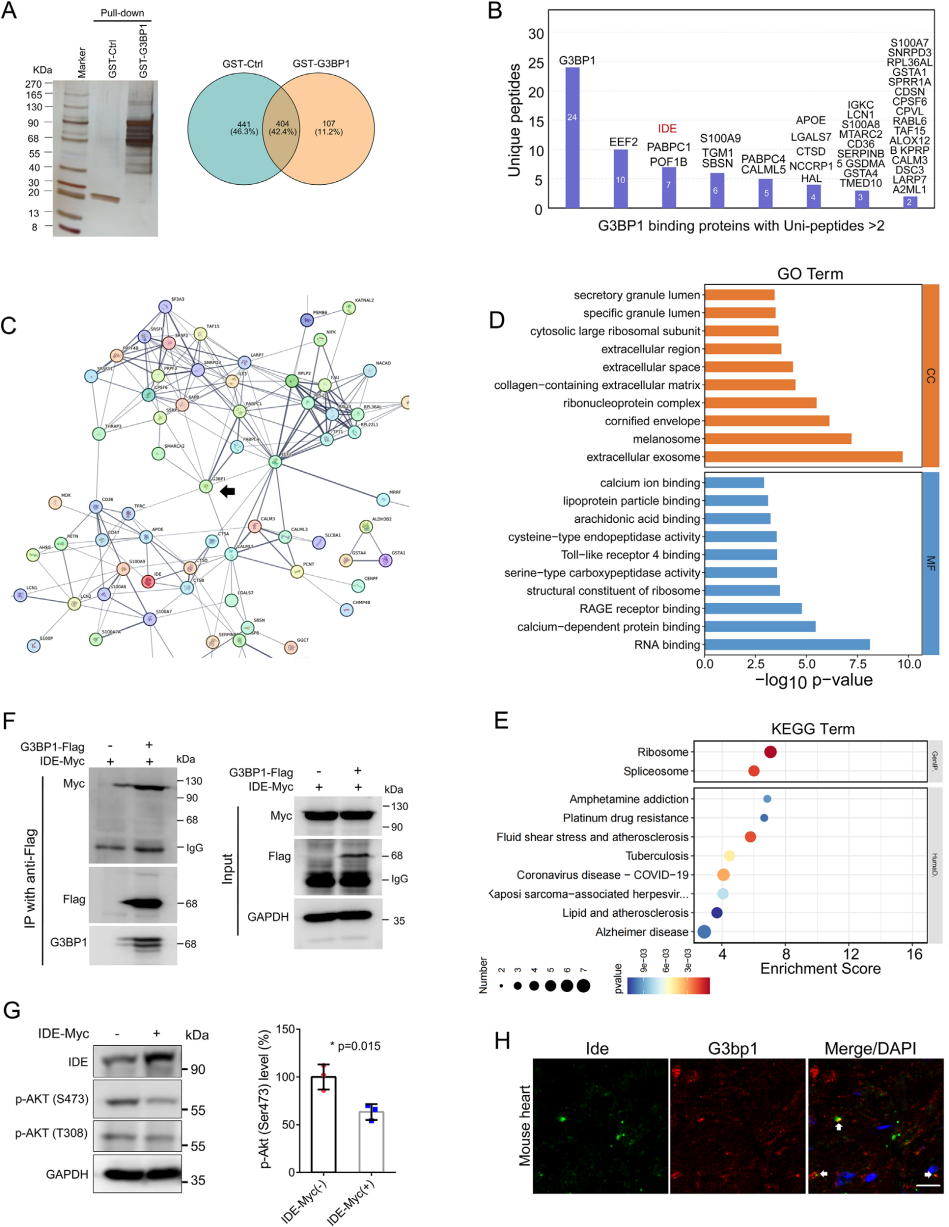
G3BP1 interacts with insulin‐degrading enzyme (IDE) in the human heart. (A) Interactome mapping of G3BP1 in the human heart using GST pull‐down coupled with mass spectrometry (MS). Purified recombinant GST‐G3BP1 was incubated with human embryonic heart tissue lysates to perform GST pull‐down and then visualized by Silver staining before MS analysis. (B) 41 Interacting proteins with unique peptides >2. (C) Protein–protein interaction (PPI) network of all 106 interacting proteins visualized using the STRING database and networkD3 R package. The central nodes of network were displayed. (D)Gene Ontology (GO) enrichment analysis on 106 interacting proteins for G3BP1 (Fisher's exact test; *p* < 0.05). (E) KEGG enrichment analysis on 106 interacting proteins for G3BP1 (Fisher's exact test; *p* < 0.05). (F) Representative images of Co‐IP validation for G3BP1 and IDE in vitro. Anti‐Flag immunoprecipitation assays were performed in HEK293T cells, followed by immunoblotting with the indicated antibodies. Representative images from three independent experiments were displayed. (G) Overexpression of IDE decreases phosphorylation of Akt at Ser473 in 293T cells. Quantification data of pAKT(S473) when normalized against GAPDH. The data are presented as the mean ± SEM, Two‐tailed Student's *t*‐test, *n* = 3, ^*^ *p* < 0.05. For each Western blot, a representative image from three independent experiments was displayed. (H) Immunofluorescence assay indicated that G3BP1 was co‐localized with IDE in mouse atrial tissues. Scale bar, 15 µm.

To explore the potential mechanisms by which G3BP1 regulates cardiac function, we next conducted GST‐pull down coupled with mass spectrometry (MS) analysis to determine the interactome of G3BP1 and its functions. GST‐tagged recombinant G3BP1 were purified and then incubated with human embryonic heart tissue lysates (26 gestational weeks). Compared with the empty‐GST group, we identified 106 proteins have specific interact with G3BP1‐GST by MS analysis (Figure 4A). G3BP1 was identified as a bait protein, which strengthens the data reliability. 38.6% (41/106) candidate proteins possess beyond 2 unique peptides (unip), including G3BP1 (unip = 23), EEF2 (unip = 10), PABPC1 (unip = 7), POF1B (unip = 7), IDE (unip = 7), S100A9 (unip = 6), SBSN (unip = 6), TGM1 (unip = 6), PABPC4 (unip = 5), CALML5 (unip = 5) (Figure 4B). Protein‐protein interaction (PPI) networks (Figure 4C) and Gene Ontology (GO) analysis revealed G3BP1 still plays a classical function as a core component of SG in the human heart, and 106 interacting proteins were significantly enriched for RNA‐binding, calcium ion binding, extracellular exosome, and ribonucleoprotein (RNP) complexes (Figure 4D). KEGG further revealed significant enrichment on Ribosome, Spliceosome, and Fluid shear stress and atherosclerosis (Figure 4E). Besides continuing to play classical functions, MS analysis provides evidence supporting that G3BP1 has binding with insulin‐degrading enzyme (IDE), a major enzyme responsible for insulin degradation. The interaction between G3BP1 and IDE was further confirmed by Co‐IP (Figure 4F). Overexpression of IDE could decrease phosphorylation of AKT at Ser473 in 293T cells (Figure 4G). Immunofluorescence assay indicated that G3bp1 was co‐localized with IDE in mouse atrial tissues (Figure 4H). These findings suggested that G3BP1 might modulate multiple physiological processes in the heart, especially insulin‐mediated PI3K/Akt/mTOR activation and glucose metabolism.

### 2.7 G3bp1 de‐Suc at K411 Induces Ras‐Related GTP Binding A (Rraga) Expression

**FIGURE 5:**
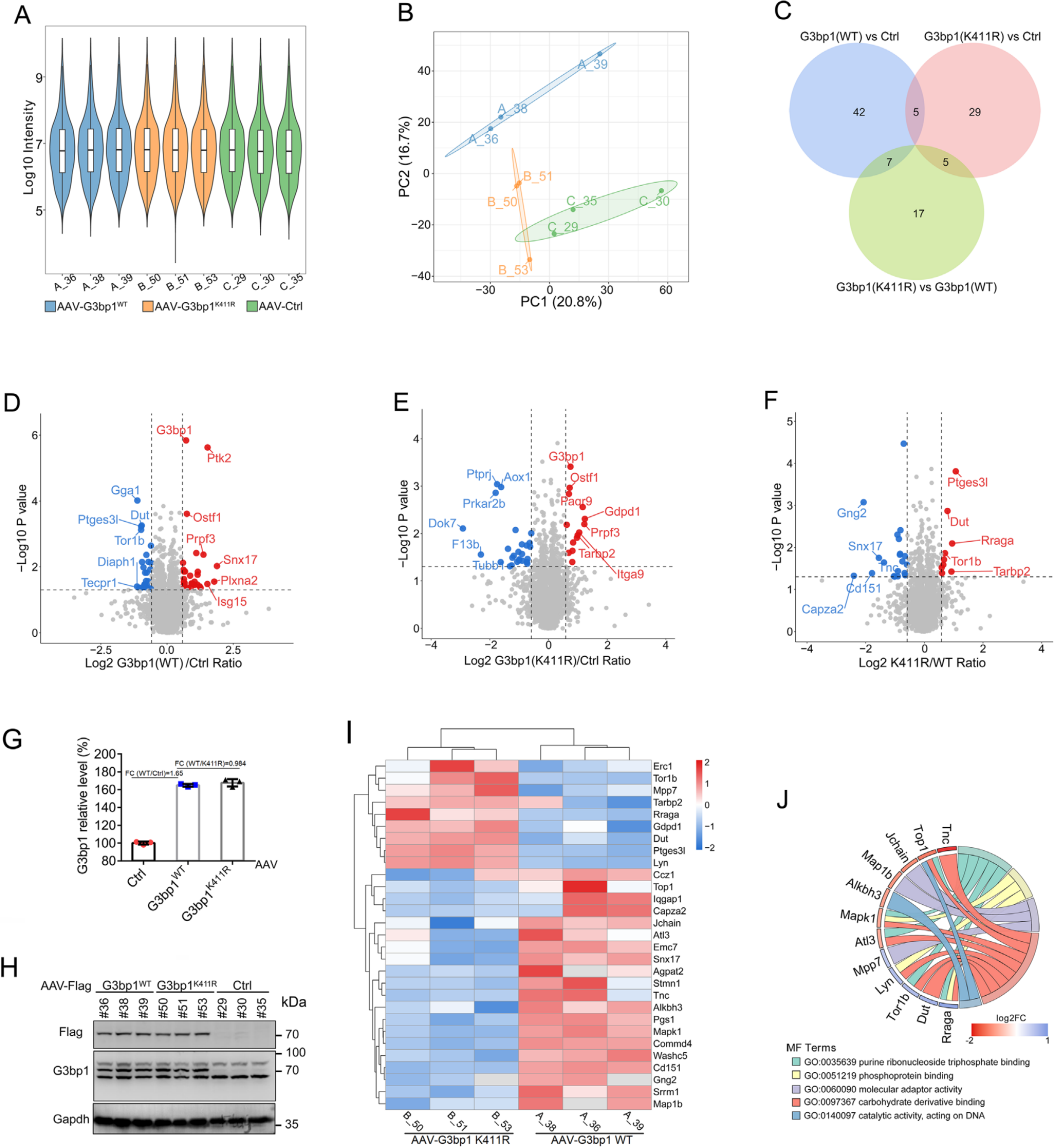
G3bp1 de‐Suc at K411 induces Ras‐related GTP binding A (Rraga) expression. (A) Summary of 4D‐LFQ (label‐free quantitation) intensity of global quantitative proteomic analysis of nine mouse heart tissues (*n* = 3 per group) injected with the three corresponding AAV viruses as indicated. (B) Score plots of principal component analysis (PCA) of AAV‐Ctrl, ‐G3bp1^WT^, and ‐G3bp1^K411R^ proteome. (C) Overview of significantly differentially expressed proteins (DEPs) in three comparison groups (fold‐change>1.5, *p* < 0.05). (D–F) Volcano plots showing the DEPs in three comparison groups, including AAV‐G3bp1^WT^ versus AAV‐Ctrl (D); AAV‐G3bp1^K411R^ versus AAV‐Ctrl (E); AAV‐G3bp1^WT^ versus AAV‐G3bp1^K411R^ (F). Up‐ and down‐regulated proteins are highlighted in red and blue, respectively. (G) G3bp1 expression was induced by 1.65‐ and 1.67‐folds in mice heart injected with AAV‐G3bp1^WT^ and ‐G3bp1^K411R^, respectively, when compared with AAV‐Ctrl according to proteomic analysis. Fold‐change = 0.984 (G3BP1 WT vs K411R). The data are presented as the mean±SEM, *n* = 3. (H) AAV injection‐mediated G3bp1 expression in mice heart was validated by western blot (*n* = 3). For each Western blot, a representative image from three independent experiments was displayed. Mice injected with AAV‐empty vector served as Ctrl group. (I) Heatmap of 29 DEPs from the comparison‌ between G3bp1^K411R^ and G3bp1^WT^ group. (J) Chord diagram showing the correlation between the enriched GO terms and DEPs from the comparison‌ between G3bp1^K411R^ and G3bp1^WT^ group.

To investigate the mechanism by which G3bp1 de‐succinylation at K411 contributes to DCM, we performed global 4D label‐free quantitative proteomic analysis of mouse heart tissues injected with AAV‐G3bp1^WT^, AAV‐G3bp1^K411R^, or AAV‐Ctrl (*n* = 3), using mouse samples collected at 12 weeks of age (Figure 5A). This analysis revealed 41 053 peptides mapping to 4,529 proteins. Principal‐component analysis effectively separated each of the nine heart samples into three distinct categories (Figure 5B). We then compare the significantly differentially expressed proteins (DEPs) across the three groups (fold‐change> 1.5, *p* < 0.05) (Figure 5C). Generally, we identified 24 downregulated DEPs and 30 upregulated DEPs in the G3bp1^WT^‐AAV_vs_Ctrl‐AAV group (Figure 5D); we detected 26 downregulated DEPs and 13 upregulated DEPs in the G3bp1^K411R^‐AAV_vs_Ctrl‐AAV group (Figure 5E), and we also identified 9 down‐regulated DEPs and 20 up‐regulated DEPs in the G3bp1^WT^‐AAV vs_G3bp1^K411R^‐AAV group (Figure 5F). Proteomic analysis indicated that G3bp1 expression was successfully induced by 1.65‐ and 1.67‐folds in mice heart injected with AAV‐G3bp1^WT^ and ‐G3bp1^K411R^, respectively, when compared with AAV‐Ctrl (Figure 5G), followed by western blot validation (Figure 5H). G3bp1 expression levels were similar between AAV‐G3bp1^WT^ and AAV‐G3bp1^K411R^ groups (fold‐change = 0.984).

Focusing on the site‐specific effects of G3bp1 K411‐Suc, we visualized 29 DEPs from the G3bp1 WT‐AAV vs G3bp1 K411R‐AAV comparison as a heatmap (Figure 5I). Notably, Rraga, a key regulator of mTORC1 activation, was significantly up‐regulated in mice heart injected with AAV‐G3bp1^K411R^. GO enrichment analysis revealed that “phosphoprotein binding,” “carbohydrate derivative binding,” and “catalytic activity, acting on DNA” were the most significantly enriched terms related to DEPs (Figure 5J), suggesting that G3bp1^K411R^ played site‐specific regulatory roles in phosphorylation‐dependent signal transduction.

### 2.8 G3bp1 de‐Suc at K411 Promotes mTORC1 Activation and Disrupt TSC2 Binding as Observed in G3bp1 Deficient Cells

**FIGURE 6:**
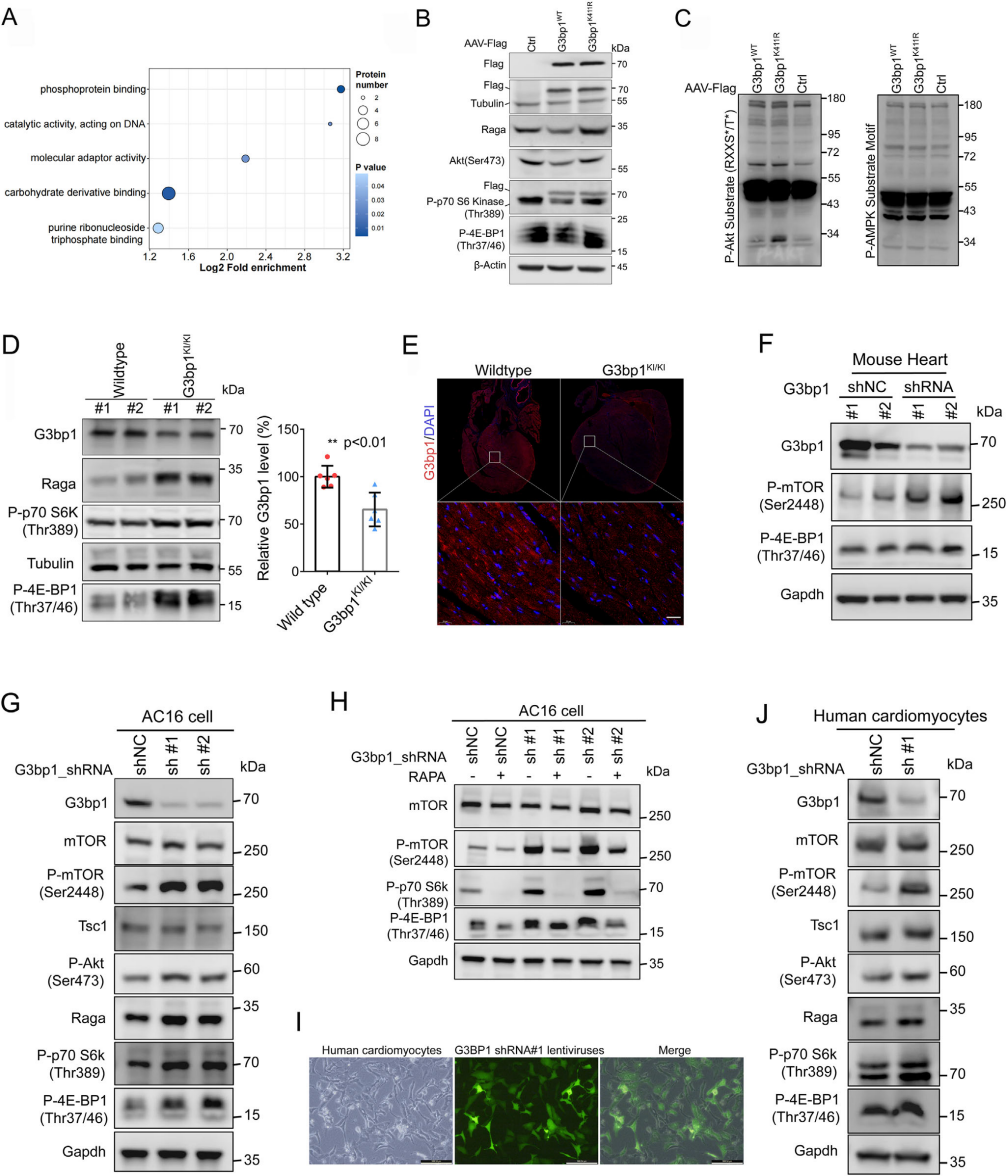
G3bp1 de‐Suc at K411 and G3bp1 knockdown promote Raga expression and mTORC1 activation. (A) Bubble plot of GO enrichment analysis on 29 DEPs from the comparison‌ between G3bp1^K411R^ and G3bp1^WT^ group. (B) Western blot confirmed the elevated expression of RragA and activation of AKT (Ser473) and mTOR downstream targets 4E‐BP1 and p70‐S6K in mice hearts injected with AAV‐ G3bp1^K411R^ when compared with AAV‐ G3bp1^WT^, a representative image from three independent experiments was displayed. (C) The downstream activities of serine/threonine kinases including AKT and AMPK, were evaluated using phospho‐(Ser/Thr) kinase substrate antibody kit. A representative image from three independent experiments is displayed. (D) Protein levels of G3BP1, Raga, and phosphorylated p70‐S6K‐pT389 and 4EBP1‐pT37/46 were analyzed by western blot in G3bp1^KI/KI^ and WT mice heart. A representative image from three independent experiments was displayed (left). Quantification data of G3BP1 expression when normalized against beta‐Tubulin (right). ^**^ *p* < 0.01 (two‐tailed Student's *t* test, *n* = 6). (E) Representative image showing decreased protein level of G3bp1 in G3bp1^KI/KI^ mice by immunofluorescence assay. Scale bar, 20 µm. (F) Immunoblot analysis of G3bp1, mTOR‐pS2448 and 4EBP1‐pT37/46 in mice injected with AAV‐NC‐shRNA and AAV‐G3bp1‐shRNA. Representative images from three independent experiments were displayed. (G) Evaluation the phosphorylation of PI3K‐AKT‐mTOR signaling pathway in G3bp1‐silenced AC16 cells by western blot as above. Gapdh served as a loading control. Representative images from three independent experiments were displayed. (H) Treatment of AC16 cells with the mTOR inhibitor Rapamycin (RAPA) with a concentration of 50 nm reversed the hyper‐activation mTOR pathway induced by G3BP1 knockdown. Gapdh served as a loading control. Representative images from three independent experiments were displayed. (I) Lentiviral‐mediated knockdown of G3BP1 shRNA in human iPSC‐derived cardiomyocytes, visualized by eGFP fluorescence from lentiviral particles. Scale bar, 388 µm. (J) Western blot analysis evaluating the phosphorylation status of the PI3K‐AKT‐mTOR signaling pathway in G3bp1‐silenced cardiomyocytes. Gapdh served as a loading control. Representative images from three independent experiments are shown.

Given the significant enrichment in “phosphoprotein binding” by DEPs (Figure 6A) and the essential role of RagA in mTORC1 activation [44, 45], we next assessed the activity of mTORC1 downstream targets in mice injected with AAV‐G3bp1. Western blotting confirmed that RragA expression was elevated in mice injected with G3bp1^K411R^‐AAV9 (Figure 6B). Moreover, AKT‐pS473, P70‐S6K‐pT389, and 4EBP1‐pT37/46 levels were markedly up‐regulated in mice administered the G3bp1^K411R^‐AAV9 vector (Figure 6B). We next use the phospho‐ (Ser/Thr) kinase substrate antibody sampler kit to investigate the downstream activities of select serine/threonine kinases in mouse hearts, including AKT, AMPK, ATM/ATR, PKA, and PKC (Figure 6C; Figure S8). The G3bp1 K411R mutant showed increased phosphorylation of AKT than WT, as determined with the corresponding phospho‐motif antibodies (Figure 6C). These findings support that K411‐Suc exerts a site‐specific regulatory role in the PI3K/AKT/mTORC1 phosphorylation signaling pathway.

We observed elevated levels of Raga expression, and phosphorylated P70‐S6K‐pT389 and 4EBP1‐pT37/46 in G3bp1^KI/KI^ mouse heart when compared with WT (Figure 6D). Notably, the protein level of G3bp1 was decreased in G3bp1^KI/KI^ mice heat by WB and immunofluorescence assay (Figure 6E), suggesting Suc at K411 might affect G3bp1 itself protein stability since the mRNA level was not significantly changed. These findings provide additional evidence to support the key role of G3bp1 K411^Suc^ on cardiac function.

To further validate our findings that G3BP1 de‐succinylation at K411 leads to a loss of function and promotes mTOR pathway activation, we first employed an AAV9‐shRNA approach to specifically silence G3bp1 expression in the hearts of male C57BL/6 mice, as performed for the aforementioned AAV‐mediated overexpression studies (Figure 6F). As expected, activation of the mTOR signaling pathway was observed. We next established stable G3BP1 knockdown in HEK‐293T cells and AC16 human cardiomyocytes using lentivirus‐delivered shRNA (Figure 6G; Figure S9A). We observed elevated RragA expression in both G3BP1‐silenced cell lines. AKT‐pS473, mTOR‐pS2448, P70‐S6K‐pT389, and 4EBP1‐pT37/46 levels were markedly up‐regulated by G3BP1 knockdown. Moreover, from the cellular level, treatment with the mTOR inhibitor Rapamycin significantly reversed the mTOR activation induced by G3BP1 knockdown in the AC16 human cardiomyocyte cell line (Figure 6H), which provides, at least in part, support for conducting future in vivo animal models with DCM and HF that carrying hyper‐activation of the mTORC1 signaling pathway. We subsequently performed lentiviral‐mediated G3BP1 knockdown in human iPSC‐derived cardiomyocytes (CMs). The efficiency of G3BP1 shRNA was assessed through eGFP fluorescence (Figure 6I; Figure S9C) and Western blotting (Figure 6J). Our data confirm that G3BP1 deficiency augments mTORC1 signaling in human iPSC‐CMs (Figure 6J), thus enhancing the clinical relevance of our findings.

### 2.9 G3BP1 de novo Mutation (p.E411G) was Identified in Patient with DCM

**FIGURE 7:**
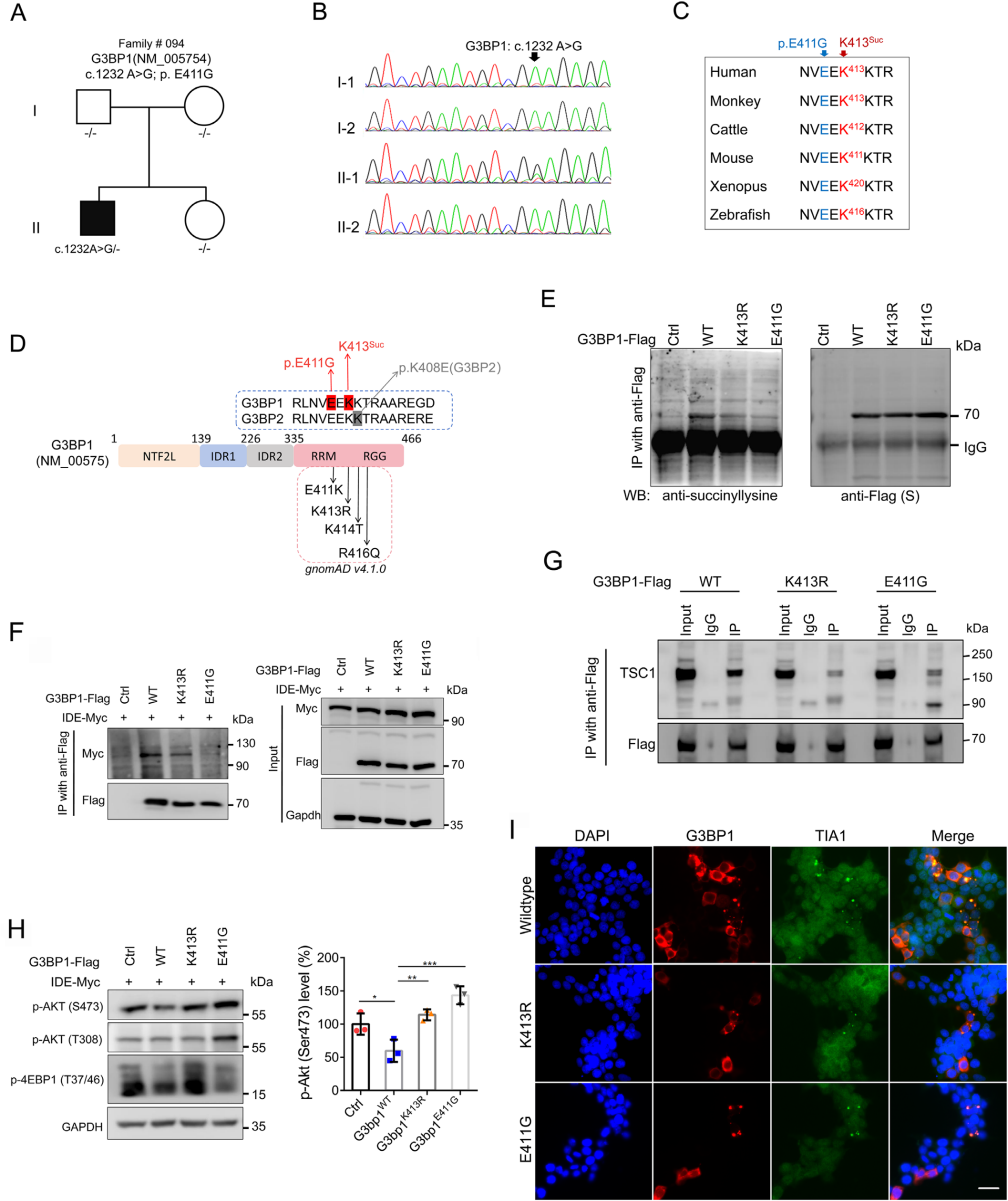
G3BP1 de novo mutation (p.E411G) from DCM patient reduces G3BP1 Suc level and IDE binding. (A) Pedigrees of the family (#094), illustrating the affected individuals and the segregation of the G3BP1 c.1232A>G mutation. (B) Sanger sequencing confirmed de novo missense mutations in G3BP1. (C) Sequence alignment of G3BP1 E411G in different species. (D) Diagram of multiple nonsynonymous mutations that locates or adjacent to the G3BP1 K413suc site from the GnomAD database v4.1.1 and literature. (E) Exogenous expression of G3BP1 E411G and K413R led to decreased G3BP1 Suc levels when compared with G3BP1 WT. 293T cells were transfected with Flag‐tagged WT and point mutants of G3BP1, and empty vector (Ctrl), and cell lysates were then immunoprecipitated with an anti‐Flag antibody, followed by immunoblotting with a pan Suc antibody. For each Western blot, a representative image from three independent experiments was displayed. (F) Co‐IP assay indicated that G3BP1 K413R and E411G interacted significantly less with IDE than did WT in 293T cells. (G) Co‐IP assay indicated that G3BP1 K413R and E411G interacted significantly less with TSC1 than did WT in 293T cells. A representative image from three independent experiments is displayed. (H) Phosphorylation of Akt was promoted in cells expressing G3bp1 E411G when compared with WT. Quantification data of p‐AKT (Ser473) when normalized against GAPDH (right). ^*^ *p* < 0.05 ^**^ *p* < 0.01^***^ *p* < 0.001 (one‐way ANOVA test, *n* = 3). A representative image from three independent experiments is displayed. (I) Representative images showing SG particle formation was not significantly disrupted in 293T cells overexpressing K413R and E411G by performing anti‐Flag and ‐TIA double immunofluorescence assay. Scale bar, 20 µm.

Previous studies have demonstrated that genetic variations play an important role in the occurrence of DCM and HF. To explore the potential genetic evidence to support the correlation between DCM and G3BP1 from population, we analyzed the data of whole exome sequencing on 43 family trios with early onset DCM and 78 sporadic cases. The ages of patients ranged from 9 to 25 years old. In two patients, we identified two missense mutations in G3BP1 (c.958C>T; p. R320C and c.1232 A>G; p. E411G; NP_005745.1) that met the filter strategy (frequency < 0.01 in gnomAD, protein‐altering variants) (Figure 7A). The p.E411G is identified in a 19‐year‐old female that appears substantial left ventricular enlargement with wall thinning, diffuse hypokinesis, and compromised cardiac contractile function, indicative of dilated cardiomyopathy. Segregation analysis did not detect p.E411G in both parents by WES and Sanger sequencing, suggesting p.E411G is a de novo mutation in the Patient (#094) (Figure 7B). p.R320C is identified as a sporadic case, and parental samples are not available for sequencing. MAFs were 0.000 for p.E411G and 16/1611904 for the p.R320C in GnomAD v4.1.0. G3BP1 E411G was highly conserved in different species (Figure 7C) and was predicted to be detrimental to protein function when evaluated by Poly‐Phen2 and MutationTaster. Since the location of p.E411G was close to its flanking Lysine (K) 413, motif‐analysis suggested that substitution at E411 might effect the Suc of K413. Moreover, multiple nonsynonymous mutations that locates or adjacent to the K413^suc^ site, including E411K, K413R, and K414Thr, have been recorded in the GnomAD database (Figure 7D). G3bp2 K408E (correspond to G3BP1 K414E, abut against K413) was identified in a patient with neurodevelopmental disorders [33].

We next overexpress the recombinant Flag‐tagged human G3BP1 protein in 293T cells. Notably, we found that the Suc level on the recombinant G3BP1 (E411G) was markedly reduced as G3BP1 (K413R) when compared with G3BP1 (WT) (Figure 7E). G3BP1 K413R and E411G interacted significantly less with IDE than G3BP1^WT^ in 293T cells (Figure 7F). Recent data indicated that G3BPs tether the TSC complex to lysosomes and suppress mTORC1 signaling [18]. Thus, we also evaluated protein interactions between G3BP1 and TS1 and TSC2 through co‐IP analysis (Figure 7G; Figure S9B). G3BP1 E411G and K413R interacted significantly less with TSC1 than G3BP1 WT. Phosphorylation of Akt at Ser473 and mTORC1 downstream target 4E‐BP1 was elevated in cells expressing G3bp1 K413R and E411G when compared with WT (Figure 7H). These findings suggest that the G3BP1 genetic mutation might take affect through disrupting the nearby lysine Suc level. We also study the effect of G3BP1 de‐Suc on classical function as a core component of SG. However, double‐immunofluorescence assay did not identify obvious changes on SG particles in cells overexpressing K413R and E411G (Figure 7I). These finding suggested that G3BP1 p.E411G might induce DCM by abrogating succinylation at K413 that then promotes mTOR activation.

## 3 Discussion

In this study, we observed entirely downregulated Suc in Mybpc3 KO and TAC‐operated mice models exhibiting cardiomyopathy and heart failure (HF), in condencen with two previous studies [11, 12], these evidences underscore a strong correlation between decreased Suc levels and impaired cardiac function. Given this inspiration from animal models, enhancing succinyl‐CoA levels represents a potential therapeutic strategy to improve cardiac function in patients.

We provide the first report that mouse G3bp1 undergoes succinylation and demonstrate that G3bp1 succinylation at K411 (corresponding to human G3BP1 K413) is required for maintaining normal cardiac function. We take steps to validate its presence in different human systems, including 293T cells, iPSC‐derived cardiomyocytes, and heart tissue samples from CHD patients, providing a potential clinical implication for G3BP1 Suc modification. Since the expression levels of the enzymes directly responsible for adding or removing succinyl modifications showed no significant change, suggesting the observed decrease in G3BP1 Suc was primarily driven by an insufficient availability of the succinyl donor pool (Suc‐CoA), rather than by altered activity of the modifying enzymes themselves in pathological cardiac states.

Through comparing the cardiac phenotypes and parameters in mice injected with AAV‐G3bp1 WT and K411R under similar levels of transgene expression, we demonstrated that K411R, mimics G3bp1 de‐succinylation status, might induce a loss‐of‐function effect on protein activity. Although existing potentially systemic toxicity analysis and off‐target interference in the liver, the majority of literature suggests that AAV safety studies tracking these endpoints typically do not reveal significant adverse effects on the heart [46, 47]. AAV‐mediated transgene expression has been utilized to improve cardiac function in animal models [48, 49]. Notably, the first clinical trials for AAV‐mediated gene therapy (designated TN‐201) for MYBPC3‐associated HCM have already been initiated [50]. According to the first Stage reports, a single administration of TN‐201 was well‐tolerated, and immunogenicity was successfully managed, with decreases observed in circulating biomarkers and a reduction in LV hypertrophy during follow‐up, highlighting that AAV‐mediated gene therapy holds immense potential for improving cardiac function in patients with HF in the future.

In contrast to the AAV‐G3bp1 injected mice, we only observed moderately significant changes on cardiac parameters in G3bp1^KI/KI^ mice, therefore, a significant impact on long‐term survival was not anticipated for the G3bp1^KI/KI^ mice alone. This discrepancy may be due to differing requirements for G3bp1 Suc signals in the heart under normal versus stress‐induced conditions. When mice are exposed to a stressful environment (such as drug treatment or dietary intervention), Suc at G3bp1 may be more required for cardiac maintenance, and de‐Suc would induce a more severe phenotype. Conversely, our preliminary data suggest that doxorubicin induction significantly accelerates the progression of heart failure in the G3bp1 KI mutant mice compared to WT controls. However, using the G3bp1 KI model, we could not differentiate between embryonic developmental effects versus post‐adult onset effects on cardiac function, since G3bp1 is also essential for embryonic development. Therefore, generating an inducible, cardiomyocyte specific knockout in adult mice represents the optimal technical strategy for definitively resolving this issue.

Proteomic analysis of heart samples from the AAV‐mediated model revealed that the de‐Suc of G3BP1 induces RRAGA expression, which in turn leads to the hyper‐activation of the PI3K/mTOR pathway. This finding was further validated in G3BP1 KI (K411R) mice and G3BP1‐silenced AC16 and 293T cell lines. Activating mutations in Rag GTPases, well‐known activators of mTOR, have been strongly linked to DCM [51, 52], and persistent mTORC1 activation is frequently observed in both animal models and patients with DCM and HF [53, 54]. Importantly, we demonstrated that Rapamycin treatment significantly reversed the mTOR activation induced by G3BP1 deficiency in human cardiomyocytes, suggesting a promising therapeutic strategy for manipulating G3BP1 activity in vivo in DCM/HF mice models characterized by mTORC1 hyper‐activation. By determining the G3BP1 interactome in the heart, we found that G3BP1 de‐Suc impairs its interaction with IDE, a highly conserved Zn^2^‐metalloprotease responsible for insulin degradation [55, 56, 57], indicating a potential regulatory role for G3BP1 in insulin‐mediated physiological processes. Although the functional consequences of this interaction warrant further investigation, our findings suggest that G3BP1 binding may promote IDE stability if mTOR pathway activity is concurrently suppressed by G3BP1. Furthermore, our data revealed that the interaction between G3BP1 and the TSC1/TSC2 complex is impaired by the K411R mutation, consistent with previous reports that G3BPs anchor the TSC2 complex to lysosomes [18]. Overall, our study provides new mechanistic insights into the regulatory role of G3BP1 in the cardiac mTORC1 signaling pathway, operating in a stress granule (SG)‐independent manner.

Previous research indicates that the net charge of G3BP1 influences its structural compactness [27, 58]. For instance, dephosphorylated wild‐type (WT) G3BP1 and the S149A/S232A mutant (located in the dimerization domain) adopt more expanded conformations in solution compared to phosphorylated WT G3BP1 [27]. Such expanded conformations facilitate protein‐RNA and protein–protein interactions, thereby promoting cooperative RNA binding and condensate assembly [27]. In our study, Suc at K413 introduces a negatively charged moiety to G3BP1; consequently, de‐Suc may similarly induce conformational changes. However, unlike the S149A/S232A mutant, the K413R mutation does not significantly alter the number of SGs under basal conditions. This discrepancy may be attributed to the location of the K413 Suc site within the C‐terminal RNA‐binding domain, whereas S149/S232 reside in the N‐terminal dimerization domain, which is essential for SG formation. Nevertheless, further investigation is warranted to elucidate the role of G3BP1 Suc in SG dynamic assembly and dissolution under stress. Additionally, the observation that the G3BP1 K411R mutation only partially reduced total succinylation levels suggests the existence of additional Suc sites, which represents a limitation of our study.

Prior to our study, no direct clinical association had been established between G3BP1 mutations and DCM. Here, we identified two G3BP1 variants, including a de nov*o* mutation (E411G), in a cohort of 43 family trios and 78 sporadic DCM cases. Notably, a mutation in the adjacent residue, G3BP2 K408E (homologous to G3BP1 K414), has been implicated in neurodevelopmental disorders [33], underscoring the pathological relevance of this structural domain. Previous studies have indeed reported a significant enrichment of de novo G3BP1 mutations in various human diseases. For instance, eight highly penetrant or de novo G3BP1 variants have been identified in patients with unexplained systemic disorders [18], and an excess of de novo G3BP1 mutations has been linked to neurodevelopmental syndromes [33, 59]. By generating a knock‐in (K411R, corresponding to human K413R) model and conducting functional analyses, we provide a robust mechanistic foundation for the role of G3BP1 in cardiac function, representing the first genetic evidence of its involvement in DCM. Nonetheless, larger‐scale sequencing cohorts are required to further validate this association at the population level.

In the heart, atrial secretory granules (ASGs) have been identified as storage sites for the cardiac‐specific hormones ANP and BNP [60, 61]. While G3BP1 is typically reported to be located in the cytosol and not secreted extracellularly, Gene Ontology (GO) analysis of G3bp1‐interacting proteins revealed the potential involvement in extracellular secretion. Intriguingly, we observed that G3BP1 co‐localizes with ANP in both human cardiomyocytes derived from hiPSCs and mouse right atrial tissues (Figure S10). Given that ANP protein levels were elevated in both G3bp1 KI and AAV mouse hearts, further investigation is needed to compare the structural properties of SGs and ASGs in the heart and to determine whether G3BP1 is involved in ANP secretion and atrial secretory granule formation.

In summary, we provide the first evidence that G3BP1 undergoes succinylation at K413 and that this modification plays a critical role in cardiac function. We report new genetic evidence to support the causative role of G3BP1 mutation in the development of DCM and HF. Mechanistically, we show that de‐Suc of G3BP1 promotes RagA expression and disrupts IDE and TSC1/2 binding, collectively leading to hyperactivation of the mTOR signaling axis. G3bp1 succinylation at the K413 site represents a promising interventional target for precisely controlling mTOR activity in patients with DCM and HF. Our results also expand our current understanding of an SG‐independent function of G3BP1 in DCM pathogenesis.

## 4 Methods

The data that support the findings of this study are available from the corresponding authors upon reasonable request.

### 4.1 Animal Models

All animal procedures were performed in accordance with the Fudan University Institutional Animal Care and Use Committee through Ethic Vote 201902010S, and were approved by the Laboratory Animal Center, Fudan University (Protocol No. 2024‐FCKYY‐172). Generation of Mybpc3‐KO and G3bp1 (K411R)‐KI mice models were supported by Gempharmatech Co., Ltd. (Nanjing, China) using the CRISPR‐Cas9 technique. Briefly, fertilized embryos (zygotes) were collected from their oviducts. Cas9 mRNA (100 ng/µL), sgRNA (50 ng/µL), and targeting ssODNs (10–20 ng/µL) were mixed and injected into the cytoplasm of fertilized eggs with both pronuclei visible in CZB (Chatot–Ziomek–Bavister) medium. The injected zygotes were then cultured in Quinn's Advantage cleavage medium (In Vitro Fertilization, Inc.) containing SCR7 (50 µm, TOCRIS) for about 24 h, and 18–20 2‐cell stage embryos were transferred into the oviduct of a pseudo‐pregnant ICR female mouse at 0.5 dpc. All mice had access to food and water. Genomic DNA was extracted from tail tips. Sanger sequencing‐based genotyping was performed for the KI model. PCR‐based genotyping was performed for Mybpc3 KO models. Homozygous mutant mice (derived from heterozygous parents) and wild‐type (WT) littermates were used in all studies. Sequences of sgRNA and primers are provided in Supporting data.

For the TAC model, 8‐week C57BL/6JGpt male mice were randomly assigned to groups TAC and Sham. Mice with an enrollment weight > 20 g were selected to avoid high surgical mortality. At day 1, mice were anesthetized with a combination of 0.1% ketamine and xylazine. After anesthesia, the hair on the neck and chest was shaved, and the area was disinfected. The trachea was isolated, and 2–3 ribs were cut open, starting at the sternum below the trachea. A minimally invasive approach was used to expose the aortic arch (using 4‐0 sutures for retraction if needed). Aortic banding was performed on the transverse aorta between the left common carotid artery and the brachiocephalic artery using a 28G needle and a 6‐0 non‐absorbable suture. The intercostal muscles and skin were sutured closed. The wound was cleaned with povidone‐iodine, and the mice were placed on a heating pad until they recovered from anesthesia. After surgery, mice received a subcutaneous injection of meloxicam and antibiotics in the nape of the neck for 3 consecutive days. The Sham‐operated group (Ctrl) underwent a median sternotomy and blunt dissection posterior to the thymus, followed by layered closure.

### 4.2 Echocardiography

Transthoracic echocardiography was performed in male mice using a Vevo3100 system (FUJIFILM VisualSonics Inc.) with an MS400 transducer following anaesthesia with 2% isoflurane gas and 0.8 L/min oxygen. Echocardiography was used to assess left ventricle (LV) systolic and diastolic functions with a two‐dimensional guided M‐mode ultrasound system (Acuson Sequoia, Siemens Medical Solutions, Mountain View, CA, USA). Two‐dimensional images from a short‐axis view of the LV were obtained at the level of the papillary muscles using a 15 MHz linear transducer at a paper speed of 100 mm/s. Echocardiography was performed before opening the chest (baseline reading) and during the course of the study for all groups. Three consecutive cardiac cycles were analyzed, and the average values were used for data analysis. Measurements were performed by independent, third‐party professional staff who were blinded to the experimental groups. Animals selected for cardiac ultrasound analysis were chosen randomly from the cohort to ensure unbiased imaging assessment without prior grouping.

### 4.3 Human Sample Collection

This study utilized human right atrial appendage (RAA) tissues for the clinical validation of G3BP1 succinylation. Since RAA incision was a prerequisite for cannulation during standard extracorporeal circulation, tissue acquisition poses no incremental risk to the patient and can be conveniently harvested during routine cardiac procedures. Our cohort comprised five patients diagnosed with Tetralogy of Fallot (ToF) who underwent elective cardiac surgery at the Children's Hospital of Fudan University (Shanghai, China) between May 6, 2024, and July 18, 2024. Congenital heart disease (CHD) phenotypes were initially identified via prenatal ultrasound at the 22nd week of gestation at the Obstetrics and Gynecology Hospital of Fudan University and were subsequently confirmed postnatally via color Doppler echocardiography.

In addition, fetal heart samples were collected to generate lysates for GST pull‐down assays. Heart samples were obtained from three human fetuses at 26 gestational weeks (GWs) following prostaglandin‐induced termination of pregnancy. All tissue samples were immediately snap‐frozen in liquid nitrogen in the operating room and stored at −80°C for subsequent protein extraction and western blot analysis. Written informed consent was obtained from all participants or their legal guardians prior to the study. All procedures were conducted in accordance with the Declaration of Helsinki and were approved by the Ethics Committee of the Obstetrics and Gynecology Hospital of Fudan University (KYY2024‐154; Shanghai, China).

### 4.4 Histopathology Staining

Heart tissues were fixed in 4% paraformaldehyde, embedded in paraffin, cut into 5 µm‐thick sections, and placed on glass slides. Tissue sections were subjected to haematoxylin and eosin (H&E), Sirius Red, and wheat germ agglutinin (WGA) or Masson's trichrome staining according to standard procedures. For Sirius Red staining, fibrosis was visualized using a Picrosirius Red Staining Kit (Polysciences, Inc.). Fibrosis was visualized using a Masson's Trichrome Staining Kit (Polysciences, Inc.).

### 4.5 Human iPSC Culture and Cardiomyocyte Differentiation

A CardioEasy Cardiomyocyte Differentiation Kit (Cellapy, China) was used to induce hiPSC differentiation into cardiomyocytes following standard procedures for cardiomyocyte‐differentiation methods. Briefly, 70%–80% confluent hiPSCs were cultured in basal differentiation medium comprising RPMI 1640 medium (C11875500BT, Thermo Fisher Scientific) and B27 supplement, minus insulin (A1895601, Thermo Fisher Scientific). hiPSCs were incubated in basal differentiation medium supplemented with CHIR‐99021 (HY‐10182, MCE) for 1 d and with Wnt‐C59 (S7037, Selleck Chemicals) for 2 days. Finally, the medium was aspirated, and the cells were subsequently fed every 1–2 days in basal RPMI 1640 medium containing B27 (17504044, Thermo Fisher Scientific). Beating cells were observed after 8–9 days of differentiation.

### 4.6 RNA Interference

HEK293T and AC16 cells were purchased from the American Type Culture Collection (ATCC) and cultured in Dulbecco's modified Eagle's medium (DMEM)/F12 supplemented with 10% fetal bovine serum (FBS) and 1% penicillin‐streptomycin‐amphotericin at 37°C in a humidified atmosphere of 5% CO_2_. To generate stable G3BP1 knockdown cell lines, HEK293T cells were co‐transfected with pCMV‐VSV‐G, pCMV‐Gag‐Pol, and shRNA‐containing plasmids using the calcium phosphate precipitation method. The viruses were collected from the culture supernatant 48 h post‐transfection, concentrated by ultra‐centrifugation for 2 h at 25 000 rpm, and resuspended in PBS. Titers were determined by infecting 293T cells and counting eGFP‐positive cells after 48 h under fluorescent microscopy. Infected cell lines were subsequently selected using puromycin. G3BP1 knockdown efficiency was verified by western blot. The shRNA sequences targeting G3BP1 are listed in Table S1.

### 4.7 Immunofluorescence Staining

Paraffin‐embedded heart‐tissue sections were rehydrated, and antigens were retrieved using heat in the presence of citrate. The sections were then blocked with 10% goat serum and incubated overnight at 4°C with primary mouse anti‐atrial natriuretic peptide (Anp), rabbit anti‐G3bp1, rabbit anti‐Ki67, rabbit anti‐cTnT, rabbit anti‐α‐actinin or rabbit anti‐Flag antibodies. On the following day, the sections were washed in 1× Tris‐buffered saline and 0.1% Tween‐20 detergent and incubated with secondary Alexa Fluor 594‐conjugated goat anti‐rabbit (Invitrogen, A‐11012) or Alexa Fluor 488‐conjugated goat anti‐mouse antibodies (Invitrogen, A‐11001). 4’,6‐diamidino‐2‐phenylindole was subsequently added for nuclear staining. The slides were observed under a fluorescence microscope, and the images were obtained in multitracking mode using a confocal laser scanning microscope (LSM880‐Airyscan; Carl Zeiss).

Cultured cells were harvested and washed twice with PBS to remove the residual medium. The cells were fixed in PBS containing paraformaldehyde (4%) and incubated with 0.8% Triton X‐100 in PBS for 15 min at room‐temperature (25°C). The cells were incubated with 10% goat serum for 60 min. Then, the cells were stained with primary and secondary antibodies as described above.

### 4.8 TUNEL Assay

Apoptotic cells in heart tissue sections were detected using a commercial TUNEL Assay Kit (BrdU‐Red, ab66110; Abcam) following the manufacturer's protocol. Briefly, 5 µm paraffin‐embedded heart sections were deparaffinized and rehydrated. The sections were fixed in 4% paraformaldehyde (PFA) for 15 min, washed with PBS, and treated with proteinase K (20 µg/mL) for 5 min. Following additional fixation in 4% PFA for 5 min at room‐temperature and subsequent PBS washes, the sections were incubated with the DNA‐labeling solution in a 37°C incubator for 1 h. After washing to remove excess TdT enzyme, sections were incubated with an anti‐BrdU‐Red antibody for 30 min in the dark. Nuclei were counterstained with DAPI. Fluorescent images were captured using a fluorescence microscope, and the percentage of TUNEL‐positive cells was quantified.

### 4.9 Enrichment of Succinylated Peptides

To enrich for Suc‐modified peptides, tryptic peptides were dissolved in NETN buffer (100 mm NaCl, 1 mm EDTA, 50 mm Tris‐HCl, and 0.5% NP‐40 [pH 8.0]) and then incubated overnight at 4°C with antibody‐conjugated beads (PTM‐402, PTM Bio, Inc., Hangzhou) at a ratio of 15 µL beads/mg protein. The antibody‐conjugated beads were washed four times with NETN buffer and twice with ddH2O. Succinylated peptides were eluted with elution buffer containing 0.1% trifluoroacetic acid. For LC‐MS/MS analysis, the resulting peptides were desalted using C18 ZipTips (Millipore), according to the manufacturer's instructions.

### 4.10 LC‐MS/MS Analysis

Tryptic peptides were dissolved in solvent A (0.1% formic acid and 2% acetonitrile in water) and directly loaded onto a homemade reverse‐phase analytical column (25 cm length, 75/100 µm i.d.). Peptides were separated using a nanoElute UHPLC system (Bruker Daltonics) and a gradient of solvent B (0.1% formic acid in acetonitrile). The solvent B concentration increased from 6% to 24% in 70 min, from 24% to 35% in 14 min, and then up to 80% in 3 min. The solvent B concentration was held at 80% for the last 3 min. A constant flow rate of 450 nL/min was used.

The peptides were subjected to capillary source analysis, followed by analysis with a timeTOF Pro (Bruker Daltonics) mass spectrometer (MS). The applied electrospray voltage was 1.60 kV. The precursor and fragment ions were analyzed using a TOF detector with an MS/MS scan range of 100–1700 m/z. The timsTOF Pro instrument was operated in the parallel accumulation serial fragmentation (PASEF) mode. Precursor ions with charge states of 0–5 were selected for fragmentation, and 10 PASEF‐MS/MS scans were acquired per cycle. The dynamic‐exclusion feature was set at 30 s.

### 4.11 GO and KEGG Enrichment Analyses

We obtained an annotated Gene Ontology (GO) proteome from the UniProt‐GOA Database (http://www.ebi.ac.uk/GOA/). Proteins were classified by GO annotation into three categories: biological processes, cellular components, and molecular functions. For each category, a two‐tailed Fisher's exact test was used to test for the enrichment of differentially expressed proteins (DEPs) against all identified proteins. GO terms with a p‐value of < 0.05 were considered to be significantly enriched.

We used an online Kyoto Encyclopedia of Genes and Genomes (KEGG) service tool (KAAS) to annotate the protein descriptions found in the KEGG database. BLAST alignments (Blastp, E‐value < 1e−4) were performed with the identified proteins. The highest BLAST‐alignment score of each alignment was selected for annotation. Pathways with corrected p‐values of <0.05 were considered to be significantly enriched. These pathways were classified into hierarchical categories according to the KEGG website.

### 4.12 Site‐Directed Mutagenesis and Immunoprecipitation

pEnter‐G3BP1‐Flag‐His human ORF clones were obtained from WZ Biosciences, Inc. (Jinan, China). A QuickChange Site‐Directed Mutagenesis Kit (Stratagene, Agilent Technologies) was used to modify the K413 Suc site to encode R and generate the mutant G3BP1 K413R variant, which was confirmed by Sanger sequencing and comparison with a reference sequence (accession number: NM_005754).

HEK‐293T cells (ATCCACS‐4500) were grown in DMEM High Glucose medium (4.5 g glucose/L) (Gibco), supplemented with 10% FBS (Gibco), 100 IU/mL penicillin, and 100 µg/mL streptomycin at 37°C in a 5% CO2 atmosphere. Then, the HEK‐293T cells were transfected with expression constructs encoding Flag‐tagged G3BP1 variants using Lipofectamine 3000 (L3000‐008, Invitrogen). After 48 h, lysates were prepared, and 500 µg of each lysate was incubated with anti‐Flag tag‐conjugated agarose beads (M20018, Abmart) for immunoprecipitation experiments following a standard protocol.

### 4.13 Injection of Mice With AAV Vectors

Six‐week‐old male C57BL/6J mice were intravenously injected with an AAV9 vector: pcAAV‐CMV‐EGFP‐P2A‐G3bp1(WT)‐3FLAG‐WPRE, pcAAV‐CMV‐ EGFP‐P2A‐G3bp1(p.K411R)‐3FLAG‐WPRE, or pcAAV‐CMV‐EGFP‐P2A‐MCS ‐3FLAG‐WPRE; pscAV‐U6‐shRNA(mG3bp1)‐CMV‐ZSgreen, pscAV‐U6‐shRNA(NC)‐CMV‐ZSgreen at a dose of 2 × 10^11^ vg/mouse via the tail vein. Following echocardiographic analysis at 4 weeks after AAV9 injection, hearts were collected to test the specificity and expression efficiency of transgenes and the knockdown efficiency of the shRNAs.

### 4.14 Succinyl‐CoA Measurement

The identification and quantification of metabolites were performed using LC–MS/MS in multiple reaction monitoring (MRM) mode. For in vivo succinyl‐CoA (Suc‐CoA) measurement, heart tissues (50 mg) were collected and homogenized in 1 mL of pre‐cooled 80% methanol. Samples were lysed on a rotating shaker at −20°C overnight, followed by centrifugation at 12 000 × g for 30 min at 4°C. For serum Suc‐CoA measurement, 50 µL of serum was mixed with 450 µL of 80% methanol, vortexed for 10 s, and centrifuged at 12 000 × g for 10 min at 4°C. The supernatant was collected, evaporated, resuspended, and analyzed by LC–MS.

Samples (5 µL) were injected into a SHIMADZU LC‐30AB LC system coupled to a QTRAP 7500 mass spectrometer (SCIEX). Chromatographic separation was performed using a HILIC BEH column (1.7 µm, 2.1 mm × 100 mm; Waters). Mobile phase A consisted of 10 mm ammonium formate in water with 0.2% ammonia; mobile phase B was 100% acetonitrile. The flow rate was 0.2 mL min^−^^1^ with a 15 min gradient: 100% phase A for 1 min, a linear increase to 90% phase B over 5 min, holding at 90% phase B for 2 min, returning to 0% phase B over 0.1 min, and holding at 100% phase A for 6.9 min. The system was operated in negative ionization mode. The specific transition monitored for Suc‐CoA was m/z 865.7492 → 407.8342. MRM data were processed using Skyline software.

### 4.15 GST Pull Down Coupled With LC‐MS/MS

In this study, GST‐tagged human G3BP1 (full‐length) was cloned into pGEX‐6P‐1 vectors. Inoculate 5 mL of LB broth with ampicillin in a culture vial with a single colony of BL21 strain of *E.coli*. Cell lysate from BL21 expressing GST (Empty pGEX‐6P‐1) was used as a control to compare with the cell lysate expressing the GST fusion protein. Both samples were processed simultaneously. Prepare 50% slurry of Glutathione Sepharose 4B from 75% slurry and incubate the clarified crude cell lysate expressing GST or GST fusion protein with 50% slurry for 2–4 h with gentle shaking. Wash the column three times with ice‐cold PBS. Add the clarified heart tissue lysate to the Glutathione Sepharose 4B column with immobilized GST/ GST fusion protein and incubate for 2–4 h at 4°C. Wash the column three times with ice‐cold PBS. Elute the interacting protein complex by incubating with 1glutathione elution buffer for 15 min at RT. The elutes thus obtained, both from a column containing both immobilized GST and GST fusion proteins, are loaded adjacent to each other on a 10% SDS PAGE gel with an appropriate molecular weight marker. Complexes eluted from the beads are resolved by SDS‐PAGE and analyzed by silver/comassie staining. The protein bands excised from the gel is subjected to an in‐gel tryptic digestion protocol. The digested peptides mixture are analyzed by LC‐MS/MS to identify the interacting proteins.

### 4.16 Immunoblotting

After separating proteins via electrophoresis and transferring them to a membrane, they were probed with one of the following primary antibodies in either 5% non‐fat milk or 5% bovine serum albumin: anti‐pan K crotonylation (PTM, 501, 502), anti‐pan K acetylation (PTM, 101), anti‐pan K Suc (PTM, 401, 402), anti‐pan ubiquitin (PTM, 1107), anti‐Flag (CST, 8146, 14793), anti‐Myc (CST, 2276, 2278), anti‐beta‐Tubulin (CST, 2146), anti‐β‐actin (CST, 4970), anti‐GAPDH (CST, 8844), anti‐α‐SMA (CST, 19245), anti‐β‐tubulin (CST, 2128), anti‐G3BP1 (CST, 61559), anti‐G3bp1 (Abcam, ab181150), anti‐TSC1(CST, 6935), anti‐TSC2 (CST, 4308), anti‐RagA (CST, 4357), anti‐Col3a1 (Abcam, ab184993), anti‐alpha‐Actinin (CST, 3134), anti‐ANP (Santa Cruz, sc‐515701), anti‐BNP (Abmart, PS00154S), anti‐MYH7 (Abmart, T56635S). The proteins were also probed using Phospho‐Akt Pathway Antibody Sampler Kit (CST, 9916), the mTOR Substrates Antibody Sampler Kit (CST, 9862), the Phospho‐(Ser/Thr) Kinase Substrate Antibody Sampler Kit (CST, 9920), and the Phospho‐MAPK Family Antibody Sampler Kit (CST, 9910).

### 4.17 Statistical Analysis

Data were analyzed and plotted using GraphPad Prism software version 5.01 (GraphPad Software, Inc., La Jolla, CA, USA). The biological replicate or sample size (n) for each statistical analysis is shown in the figure legends. Survival curves were constructed using Kaplan–Meier analysis with the log‐rank (Mantel‐Cox) test. All summary Data are presented as the mean ± SEM and were evaluated using a two‐tailed unpaired Student's *t*‐test for two groups comparisons and one‐way ANOVA between multiple groups. Differences were considered statistically significant at *p* < 0.05.

## Author Contributions

X.Z. conceived and designed the study. Y.Z., C.Y., Y.C., Z.L., B.W., and Y.S. performed the experiments. X.Z., K.C., and Y.Y. interpreted the clinical data. X.Z., Y.Z., Y.C., and J.Z. analyzed the data. X.Z. drafted the manuscript. All authors reviewed and approved the final version of the manuscript.

## Ethics Statement

For studies of affected individuals and their families, written informed consents were obtained from all participants prior to the start of the study. All procedures in the study were approved by the Medical Ethics Committee of Obstetrics and Gynecology Hospital of Fudan University (kyy2024‐154) (Shanghai, China). For animal experiments, all experimental protocols were conducted in accordance with the institutional ethical guidelines and approved by the Institutional Animal Care and Use Committee of Laboratory Animal Center, Fudan University (2024‐FCKYY‐172).

## Conflicts of Interest

The authors declare no conflicts of interest.

## Supporting information




**Supporting file 1**: advs75617‐sup‐0001‐SuppMat.docx

**Supporting file 2**: advs75617‐sup‐0002‐Data_Raw WB gels.pdf

## Data Availability

The data that support the findings of this study are available from the corresponding author upon reasonable request.
