# Supplementary material for: G3BP1 Succinylation at K413 is Critical for Cardiac Function by Modulating PI3K‐AKT‐mTOR Signal Axis
Source: Adv Sci (Weinh). 2026 May 10;13(43):e19856. doi: 10.1002/advs.202519856 (PMC13335847; doi:10.1002/advs.202519856)
Supplement: Supplementary file 1 — Supporting File 1: advs75617‐sup‐0001‐SuppMat.docx. [file ADVS-13-e19856-s002.docx]

**Supplementary Figure And Table Legends**

**Figure S1.** Generation of TAC-operated mice model. (A) Schematic illustration of the transverse aortic constriction (TAC) procedure. The constriction was created between the left common carotid artery and the brachiocephalic artery using a 28G needle to induce pressure overload. (B) Echocardiographic analysis confirmed decreased ejection fraction (EF) and fractional shortening (FS) in TAC mice compared with the Sham group 4 weeks post-operation. Data are presented as mean ± SEM (n = 4 mice per group, with each sample analyzed in triplicate). Statistical significance was determined using a two-tailed Student’s t-test: ***p < 0.001. (C) Representative Coomassie blue staining of heart tissues from TAC-operated and Mybpc3 KO mice, compared with their respective Sham and WT control groups.

**Figure S2**. Validation of G3BP1 modifications by IP assay. (A) Assessment of G3BP1 succinylation (Suc) in RAA heart tissues from five ToF patients using endogenous anti-G3BP1 immunoprecipitation (IP). Succinylation signals were detected in three patients (indicated by red arrows: no. 1, 3, and 4). (B) Evaluation of G3BP1 acetylation by IP assay in HEK-293T cells overexpressing Flag-tagged G3BP1 wild-type (WT) and the K413R mutant.

**Figure S3.** Dysregulation of mitochondrial-related pathways in Mybpc3 KO mouse hearts. (A) Summary of 4D-LFQ (label-free quantification) intensities from global quantitative proteomic analysis of 8 mouse heart tissues (n = 4 per group). (B) Principal component analysis (PCA) score plots of the proteomes from WT and Mybpc3 KO mice. (C) Summary of peptide and protein identification statistics. (D) Overview of significantly differentially expressed proteins (DEPs) between comparison groups (fold-change > 1.5, p < 0.05). (E) Volcano plots displaying DEPs between Mybpc3 KO and WT mice. Up-regulated and down-regulated proteins are highlighted in red and blue, respectively. (F) Gene Ontology (GO) enrichment analysis of 167 DEPs identified between Mybpc3 KO and WT groups. Terms related to mitochondria were significantly enriched. The significance of pathway enrichment was assessed using Fisher’s exact test.

**Figure S4**. Expression of key enzymes involved in succinyl modification and Suc-CoA metabolism in Mybpc3 KO mice heart. Relative protein levels of key enzymes involved in succinyl modification and succinyl-CoA (Suc-CoA) metabolism—CPT1A, SIRT5, HDAC1, SUCLA2, SDHA, and OGDH—in WT and Mybpc3 KO mouse hearts, as derived from global proteomic analysis. Data are presented as mean ± SEM (n = 4 mice). Statistical significance was determined using a two-tailed Student’s t-test (***p < 0.001; ns, not significant).

**Figure S5**. Targeted quantification of Succinyl-CoA in heart and serum samples by LC-MS. (A) Representative mass spectrometry peaks of Succinyl-CoA standards at varying concentrations (1 μM, 10 μM, 0.1 mM, and 1 mM). (B) Representative mass spectrometry peaks showing Succinyl-CoA intensities in heart and serum samples from wildtype, Mybpc3 KO, Sham, and TAC mice. (C–F) Relative Succinyl-CoA levels in heart and serum samples of Mybpc3 KO and TAC-operated mice. Data are presented as mean ± SEM (n = 5 mice). Statistical significance was determined using a two-tailed Student’s t-test: *p < 0.05; **p < 0.01; ***p < 0.001.

**Figure S6.** Representative images of α-actinin and TUNEL staining in the hearts of mice injected with AAV-Ctrl, AAV-G3bp1 WT, AAV-G3bp1 K411R, and in wild-type and G3bp1^KI/KI^ mice. Scale bar, 50 µm.

**Figure S7**. Ki-67 staining in two G3bp1 transgenic mice models. (A) Representative Ki67 staining images in mouse hearts injected with AAV-Ctrl, AAV-G3bp1 WT, and AAV-G3bp1 K411R. Quantitative analysis of Ki67 staining (n = 6 mice per group). Data were normalized and are presented as mean ± SEM. Statistical significance was assessed by one-way ANOVA (ns, not significant). Scale bar, 50 µm. (B) Representative Ki67 staining images in hearts of G3bp1^KI/KI^ and WT mice. Quantitative analysis of Ki67 staining (n = 8 mice per group). Data were normalized and are presented as mean ± SEM. Statistical significance was assessed by one-way ANOVA (ns, not significant). Scale bar, 50 µm.

**‌Figure S8.** Assessment of downstream serine/threonine kinase activity in mouse hearts injected with AAV-G3BP1. Phospho-(Ser/Thr) kinase substrate antibody arrays were used to assess the activity of the ATM/ATR, PKA, and PKC pathways. Immunoblotting with anti-G3BP1 and anti-Flag antibodies confirmed AAV-mediated G3BP1 expression in mouse heart tissues, with β-tubulin serving as the loading control. Representative immunoblots from three independent experiments are shown.

**Figure S9**. (A) Western blot analysis of the phosphorylation status of the PI3K-AKT-mTOR signaling pathway in G3bp1-silenced human 293T cells. GAPDH served as the loading control. Representative blots from three independent experiments are shown. (B) Co-immunoprecipitation (Co-IP) assay to evaluate the interaction between G3BP1 and TSC2. HEK-293T cells were transfected with Flag-tagged G3BP1 wild-type (WT) or its point mutants. Cell lysates were subjected to immunoprecipitation with an anti-Flag antibody, followed by immunoblotting with an anti-TSC2 antibody to detect endogenous TSC2. (C) Determination of the titers of G3BP1_shRNA#1 lentivirus particles. 293T cells were infected with serial dilutions of concentrated lentivirus, and eGFP-positive cells were counted under fluorescent microscopy 48 hours post-infection.

**Figure S10**. G3BP1 is potentially involved in the assembly of atrial secretory granules. (A–B) Double immunofluorescence staining demonstrating the co-localization of G3BP1 and ANP in human induced pluripotent stem cell (hiPSC)-derived cardiomyocytes (A) and human right atrial tissue (B). Scale bars: 5 μm (upper panels) and 7.5 μm (lower panels).

**Table S1**. Sequences of sgRNAs for G3bp1 knock-in (KI) mouse model generation, and primers used for PCR amplification and shRNA-mediated gene silencing.

Figure S1


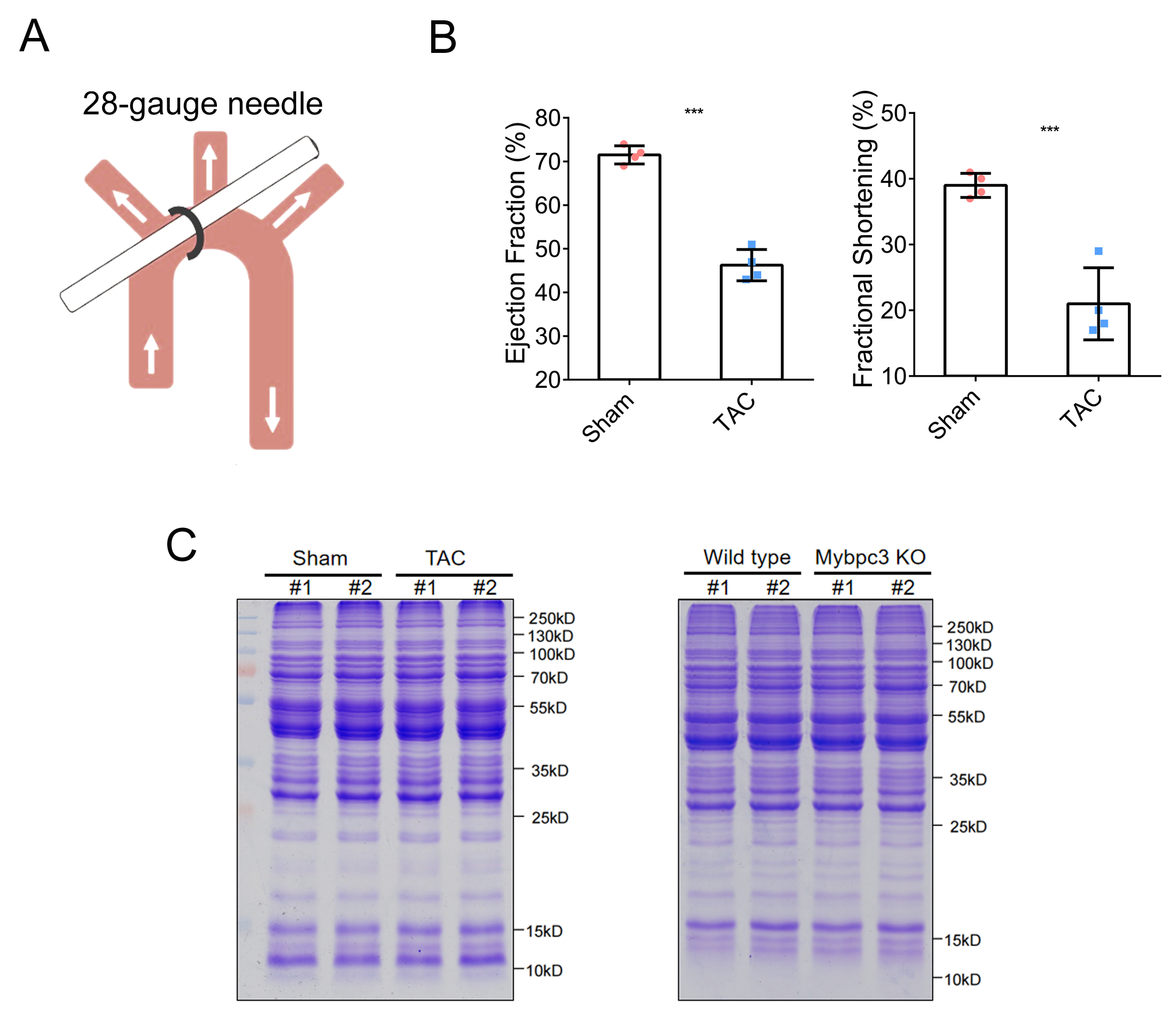


Figure S2


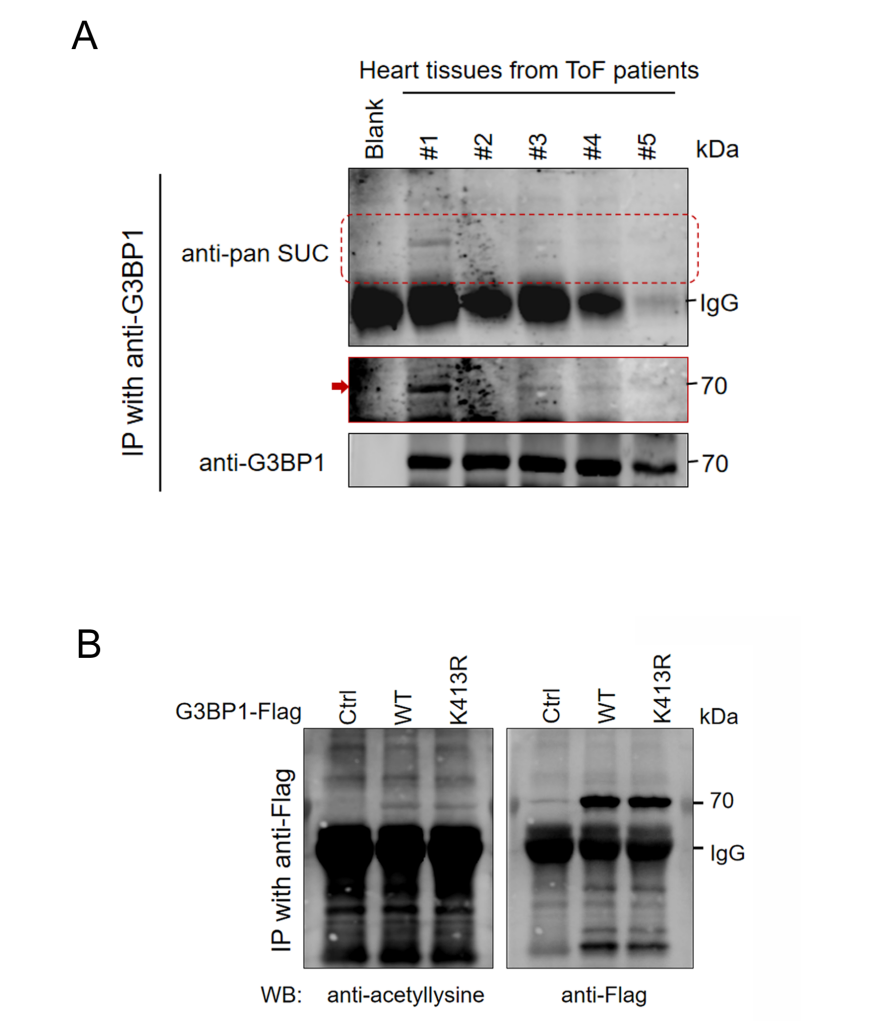


Figure S3


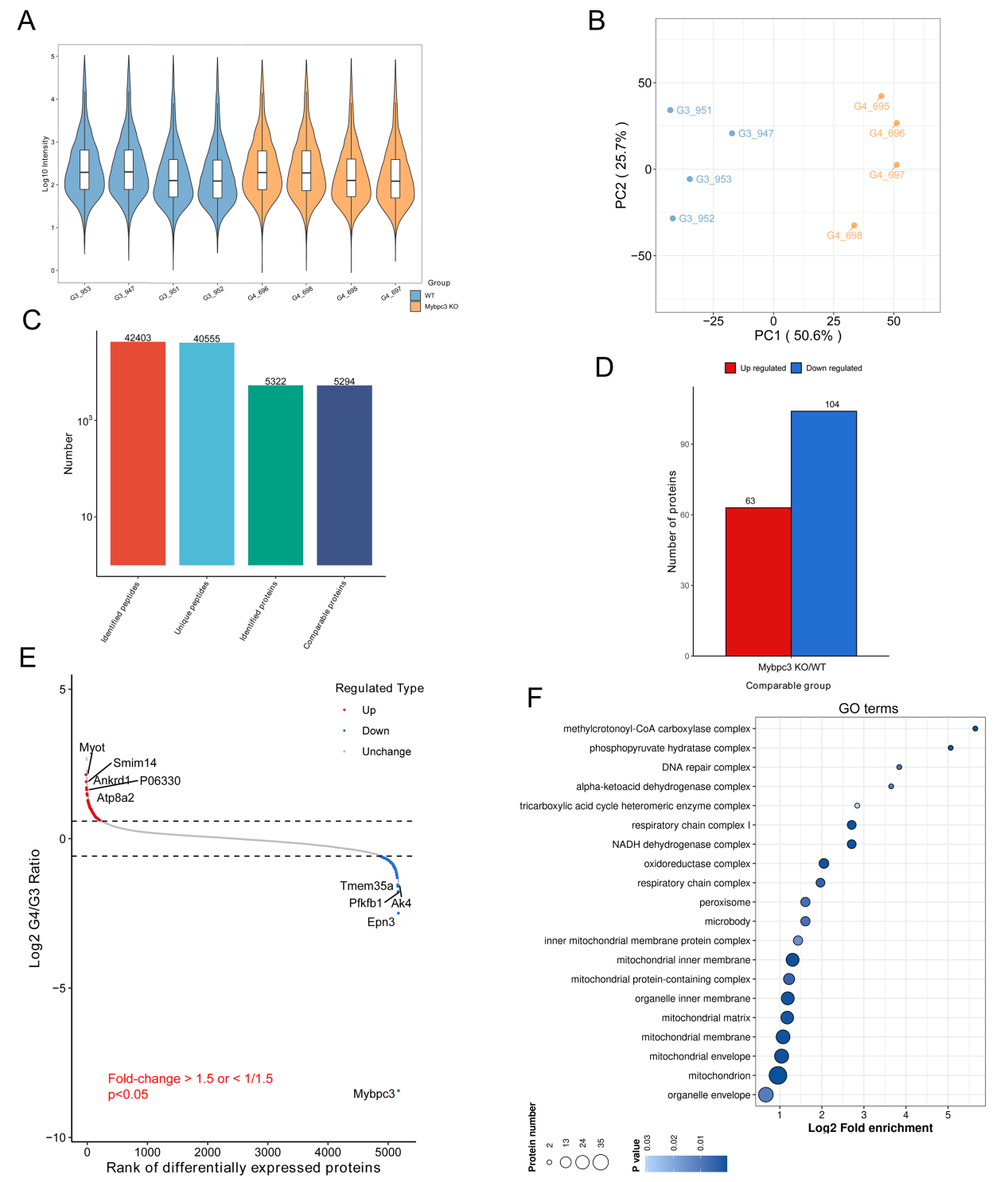


Figure S4


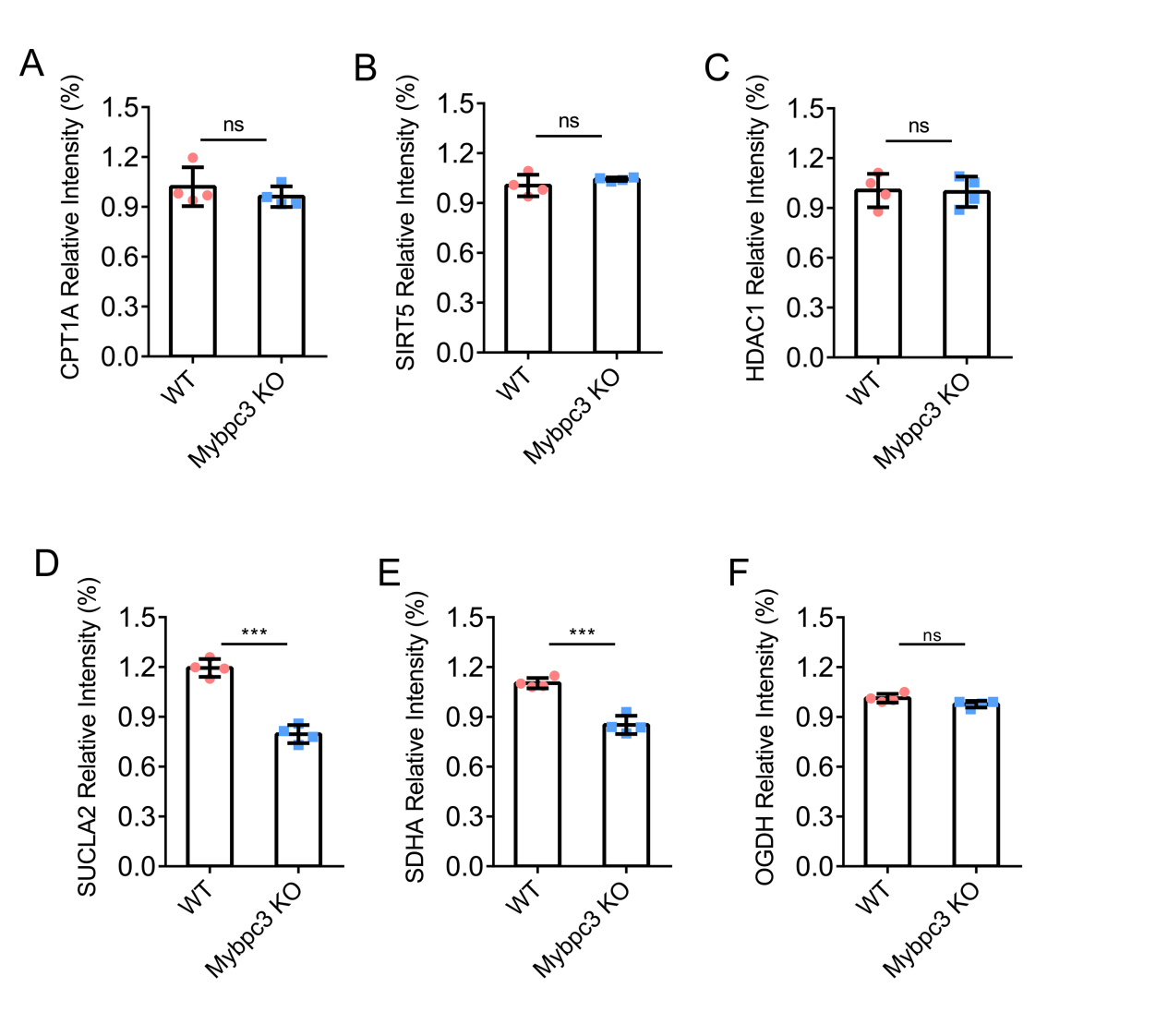


Figure S5


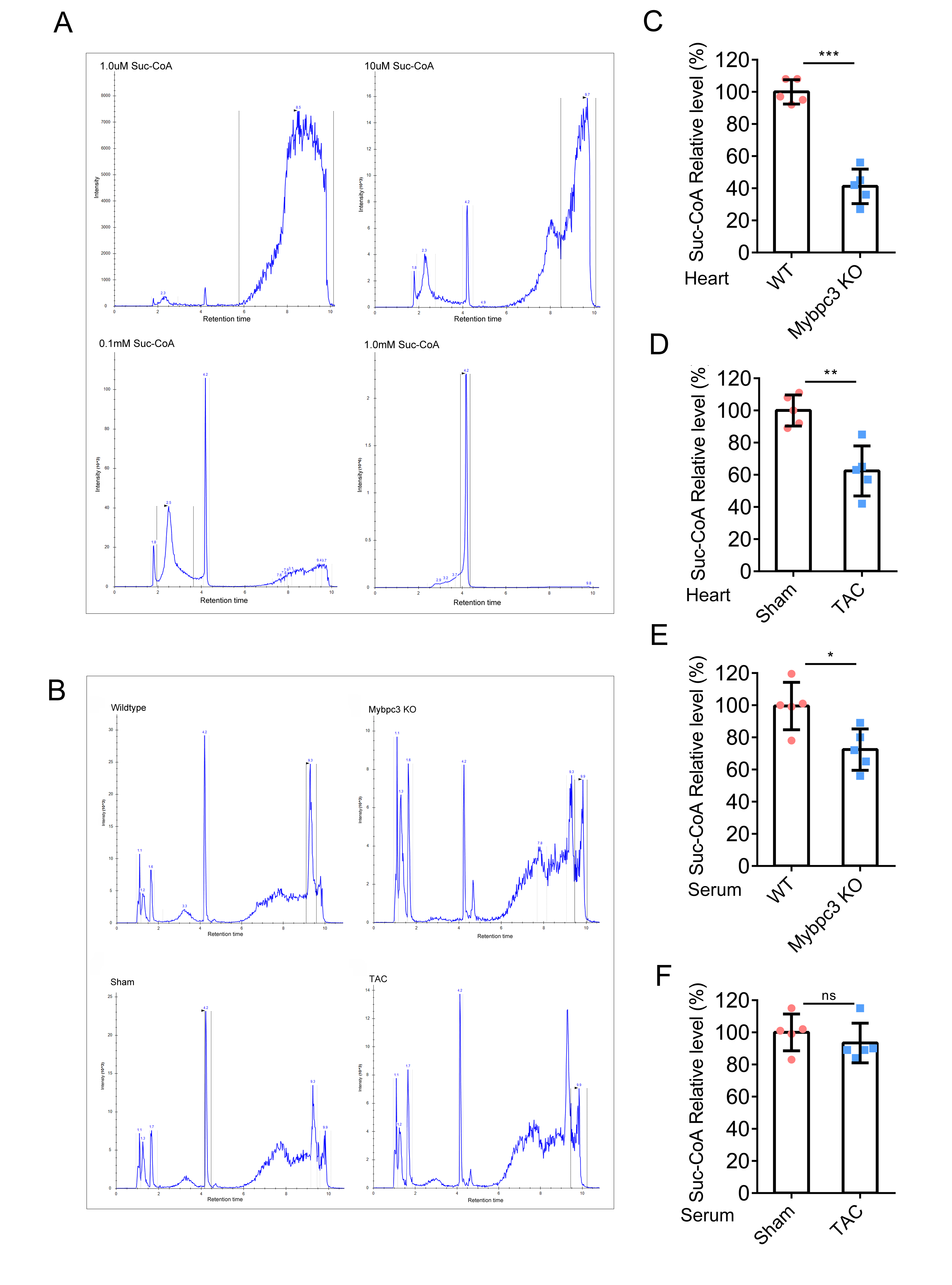


Figure S6


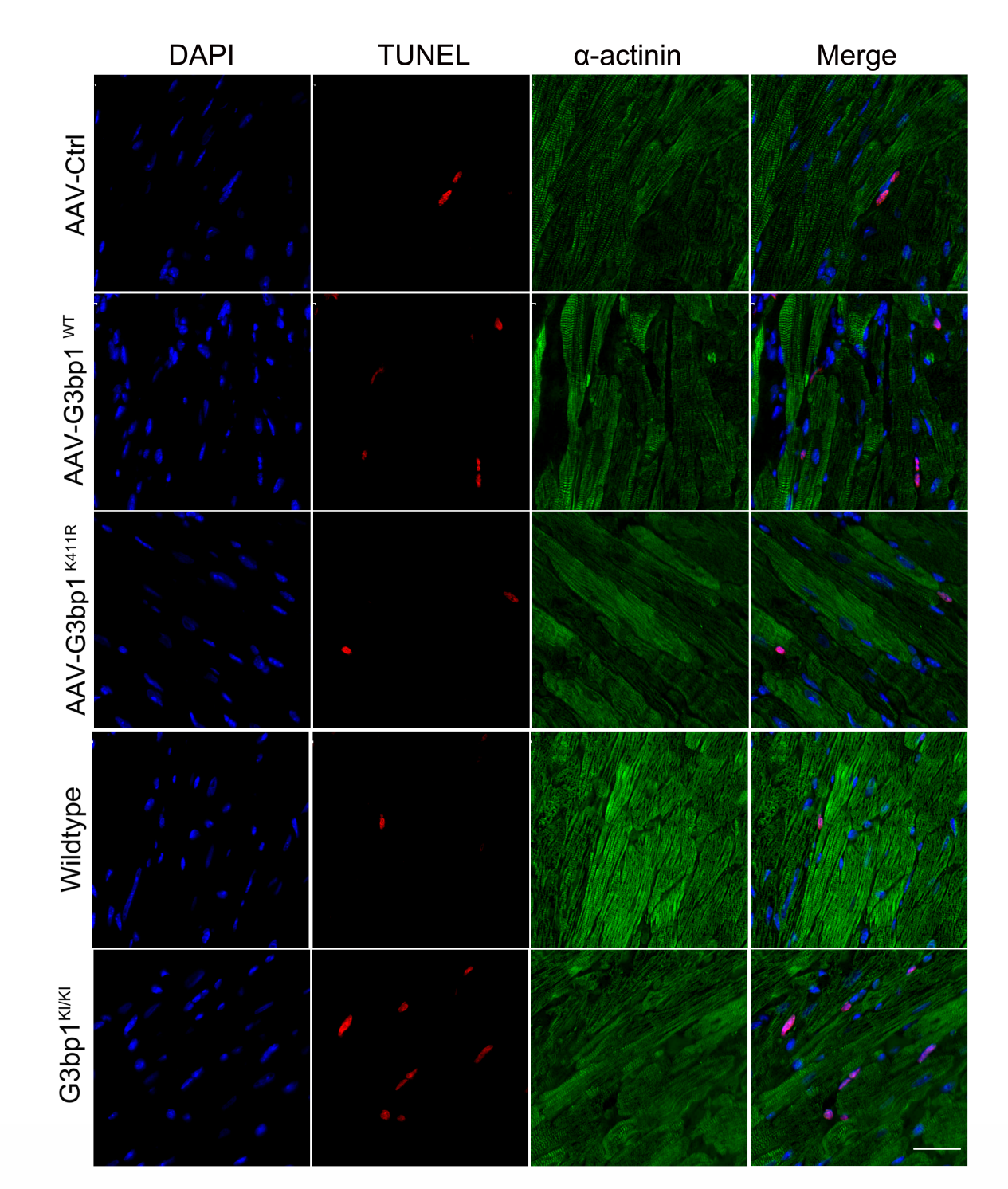


Figure 7


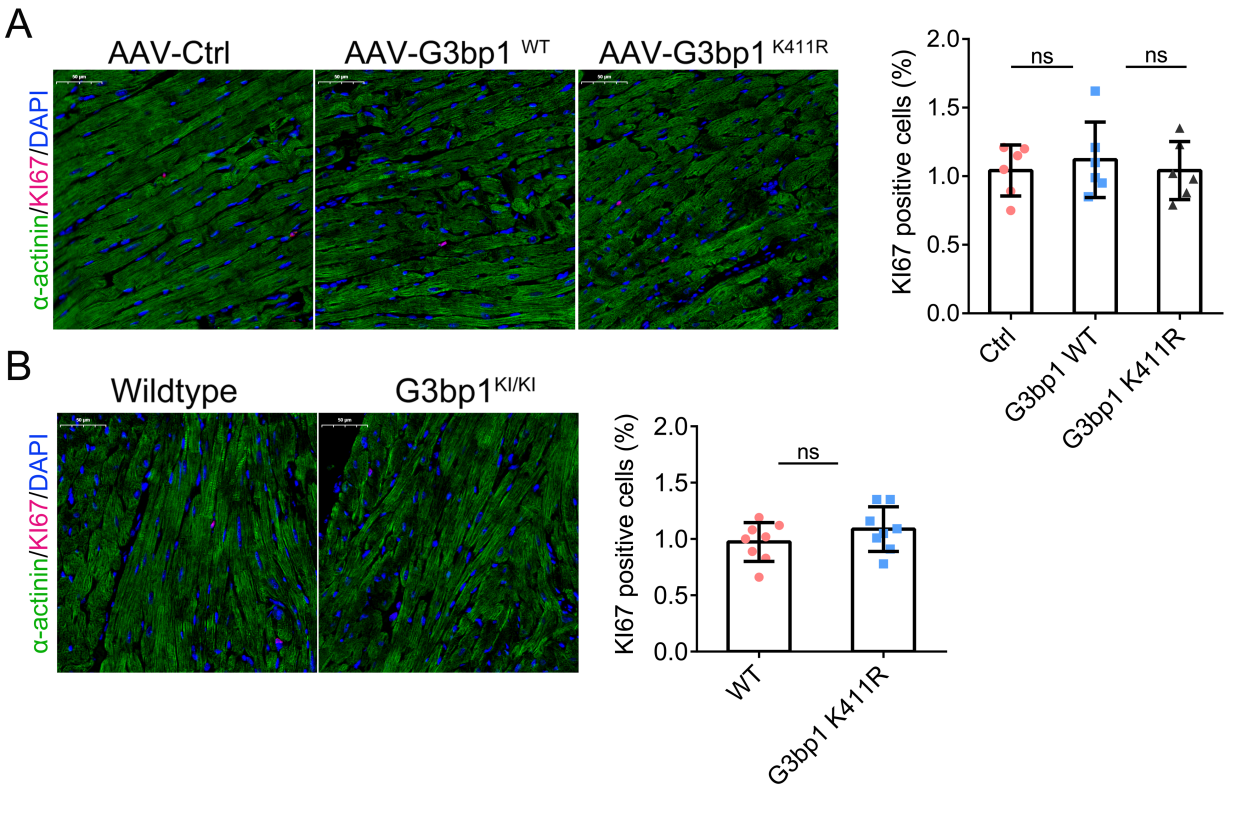


Figure S8


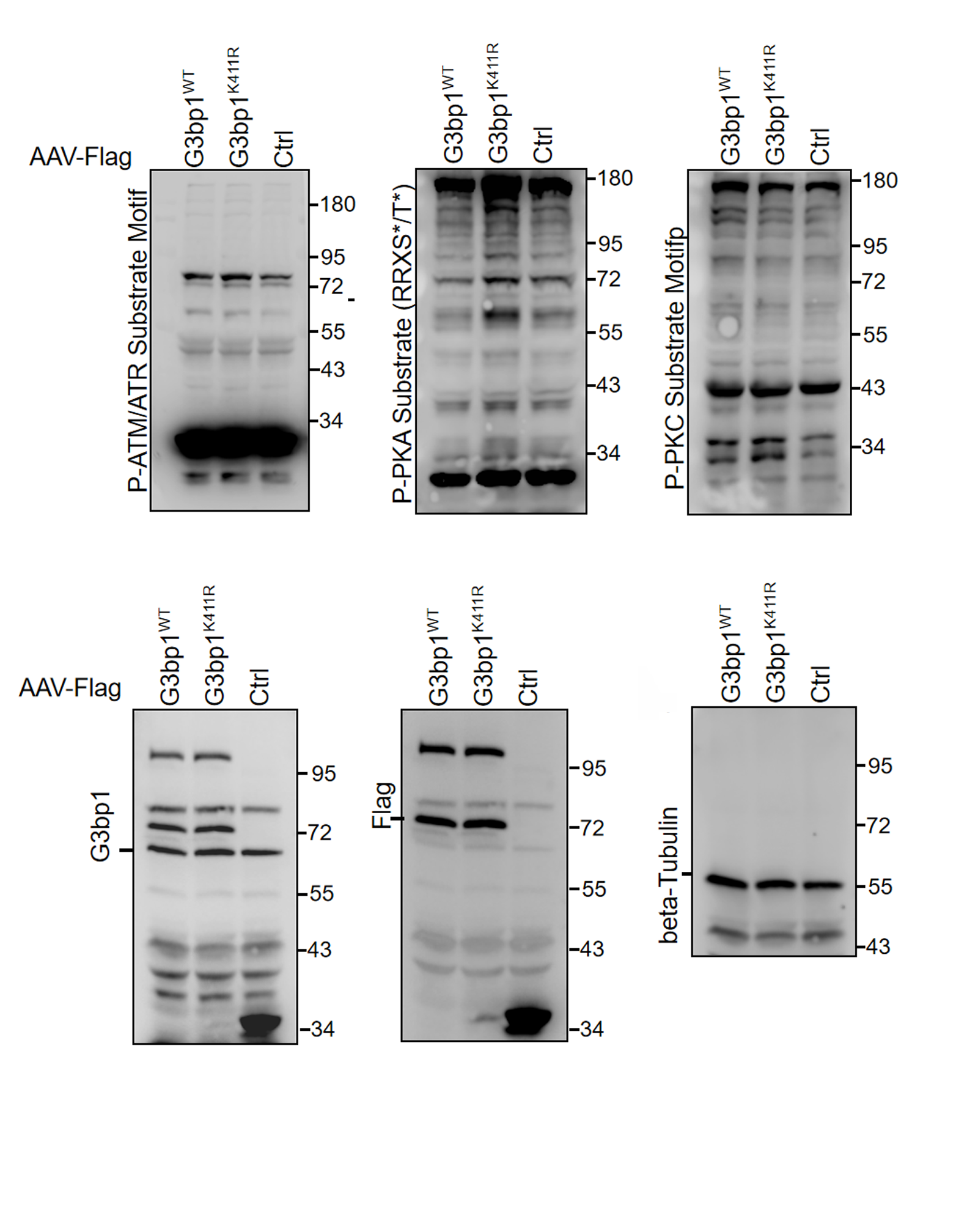


Figure S9


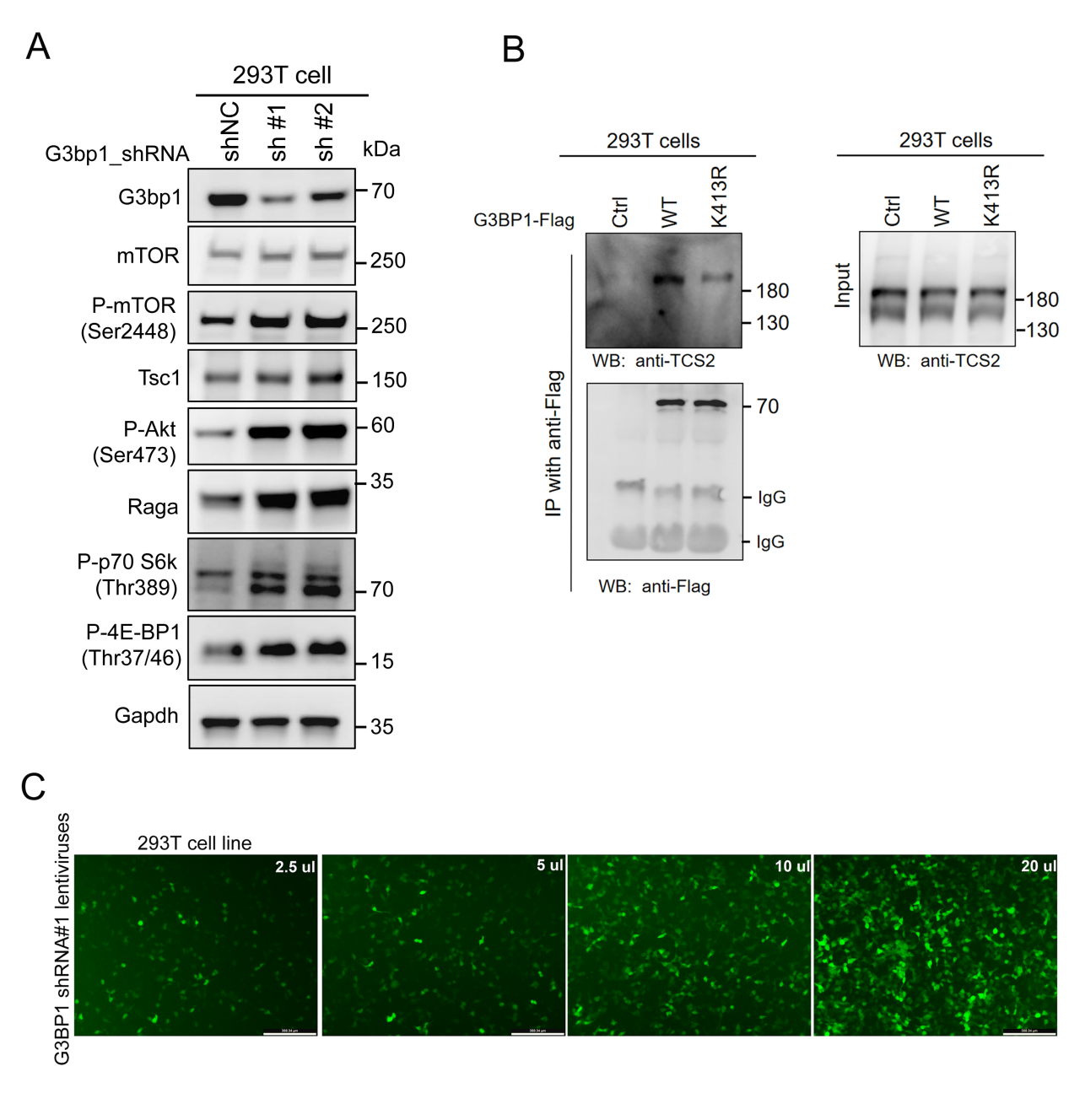


Figure S10


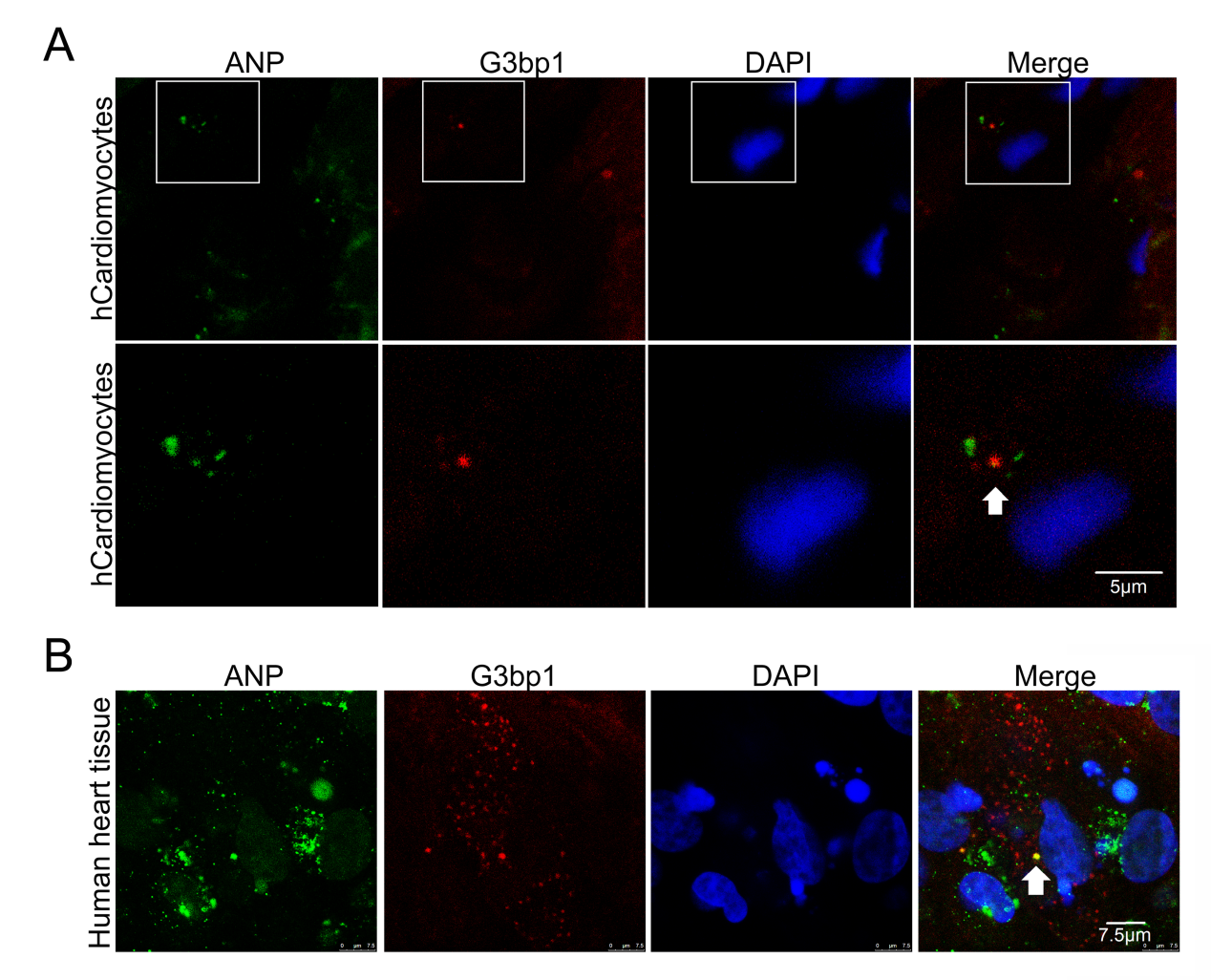


Table S1.

| gRNA name (5') | gRNA sequence (5'→3') for G3bp1 KI model | PAM |
| --- | --- | --- |
| S1 | GGCCTCCAGGTCCCCGAAGG | CGG |
| S2 | GATAACCGCCTTCGGGGACC | TGG |
| Primers name | Primers sequence (5'→3') |  |
| G3bp1(K411R)_F | CTTGCATGGTTCACCATATCTGC |  |
| G3bp1(K411R)_R | GTTGGGAGCACACACCATGA |  |
| Mybpc3(KO)_F | AGCTCTGAGTGTCTGCCACATGAG |  |
| Mybpc3(KO)_R | CCCTCAGCCACATATCTCTACCAA |  |
| Target G3bp1 shRNA | shRNA sequences |  |
| hG3BP1 shRNA #1 | AGTGCGAGAACAACGAATAAA |  |
| hG3BP1 shRNA #2 | CGGGAATTTGTGAGACAGTAT |  |
| mG3BP1 shRNA #1 | cctcagagagatcagagagtt |  |
| mG3BP1 shRNA #2 | gcctgatgattctggaacttt |  |
