# Supplementary material for: G3BP1 Succinylation at K413 is Critical for Cardiac Function by Modulating PI3K‐AKT‐mTOR Signal Axis
Source: Adv Sci (Weinh). 2026 May 10;13(43):e19856. doi: 10.1002/advs.202519856 (PMC13335847; doi:10.1002/advs.202519856)

Fig.1E and F

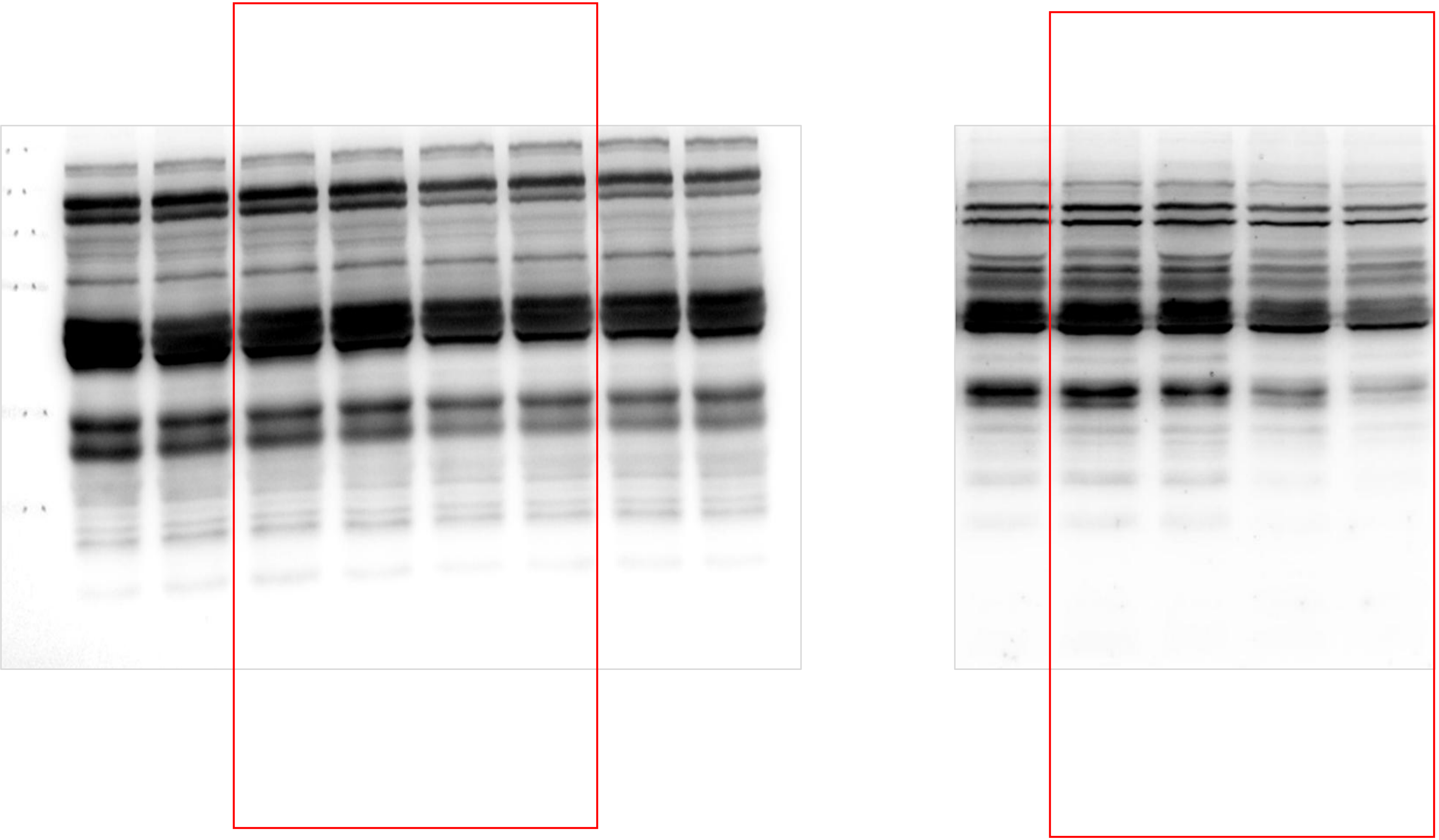

Fig.1G and H

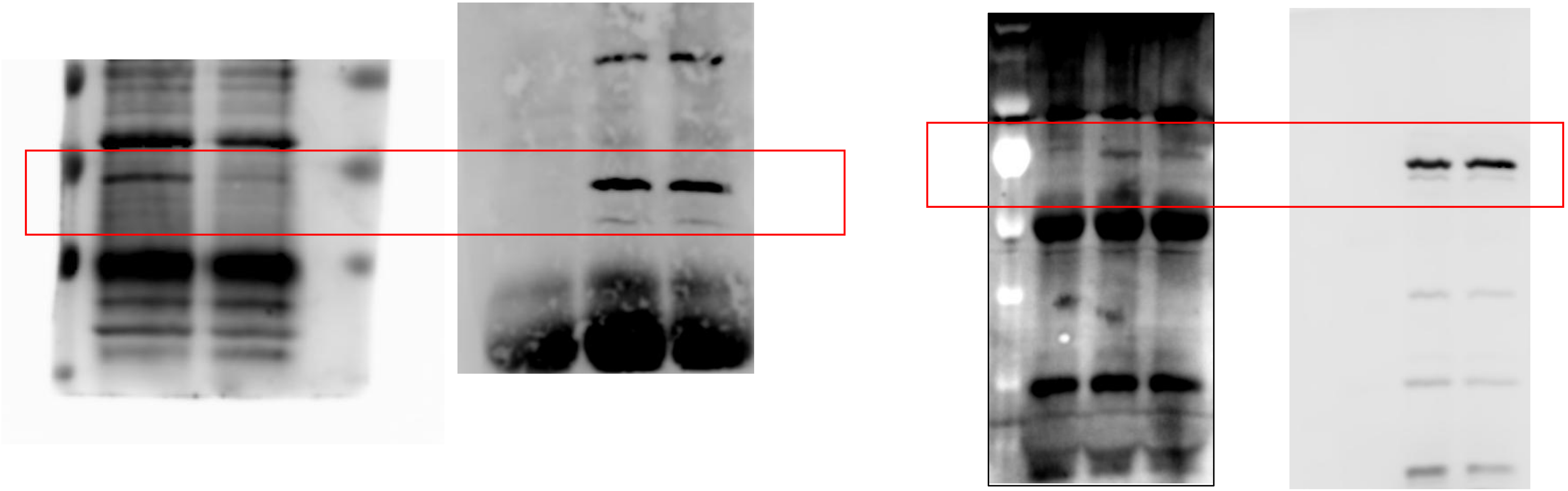

Fig.1J and N

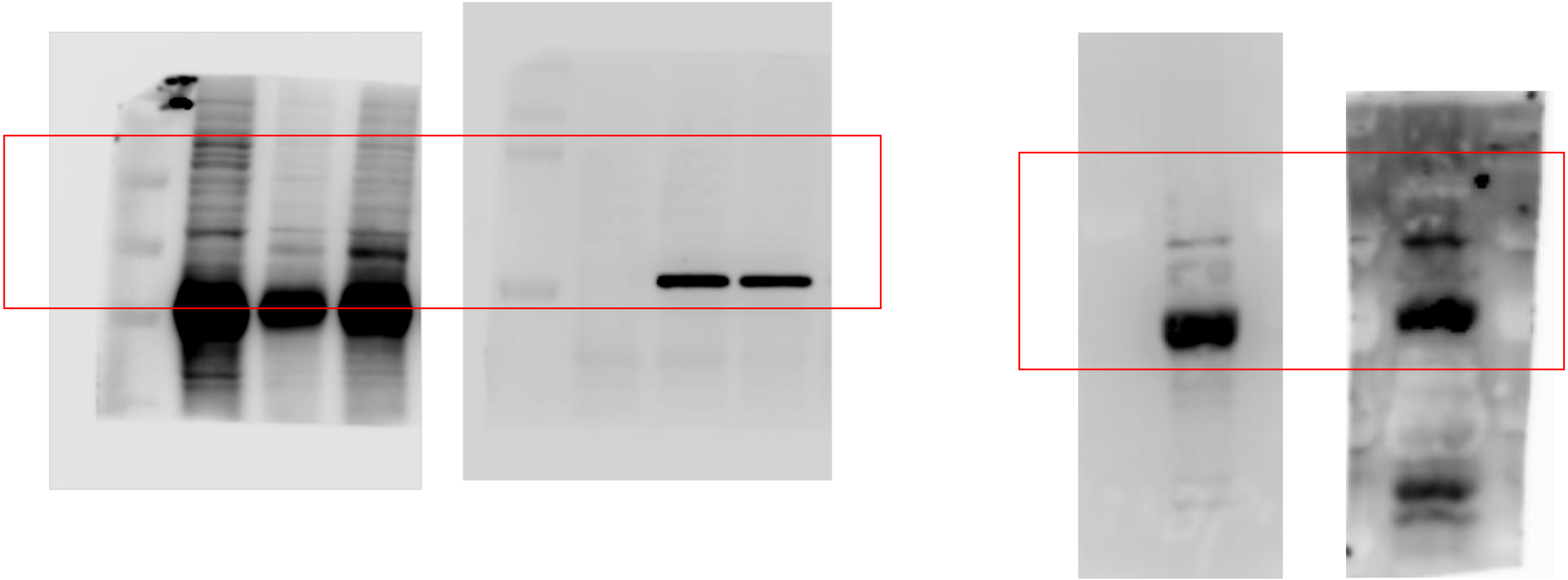

Fig.1K

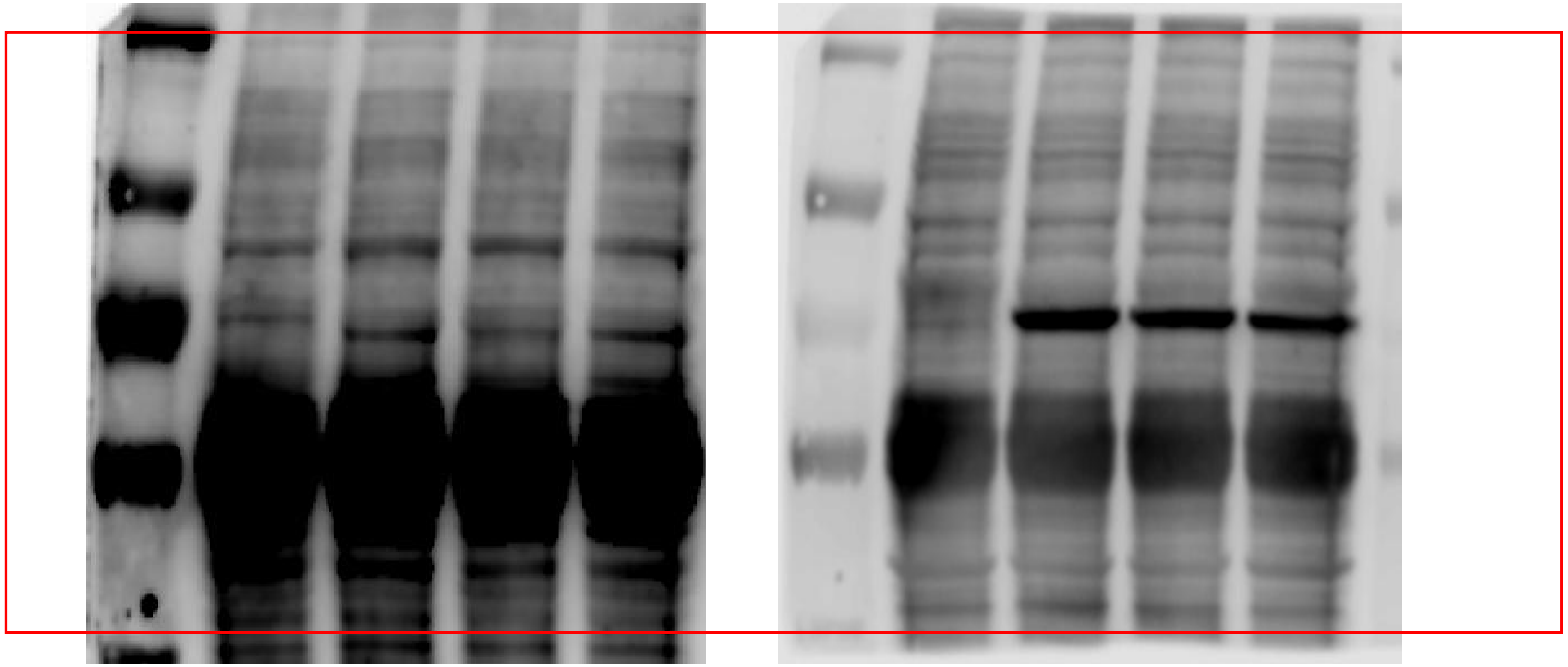

Fig.1O

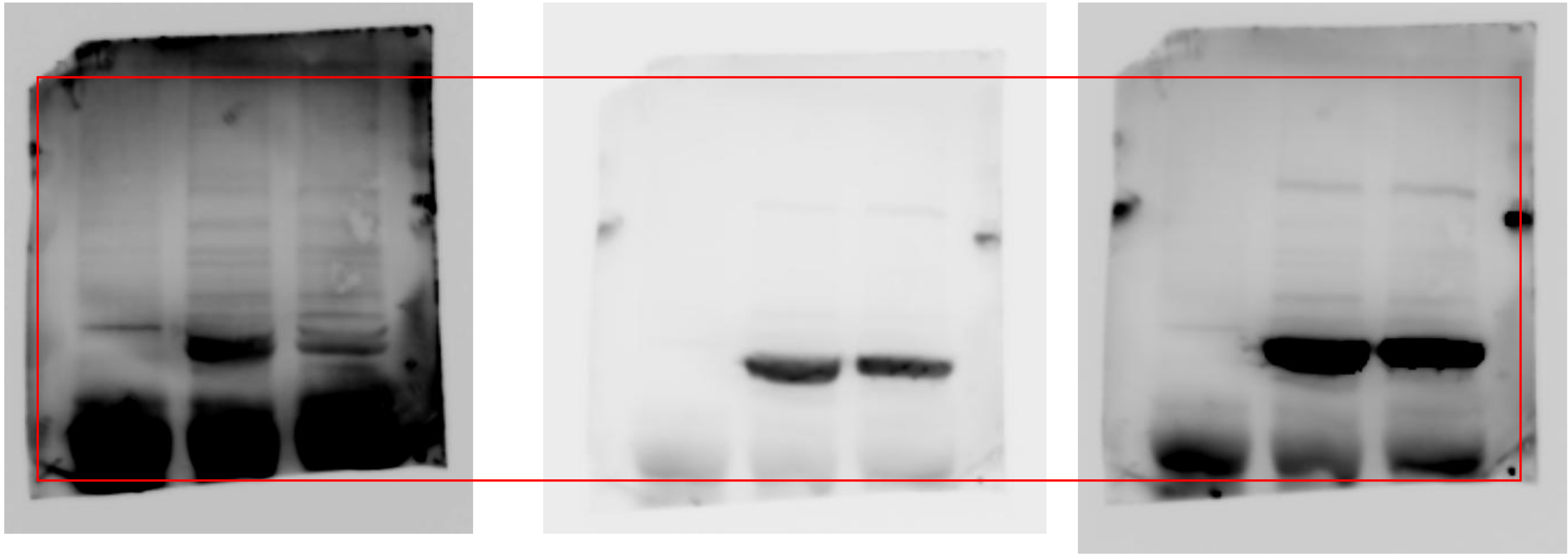

Fig.1P

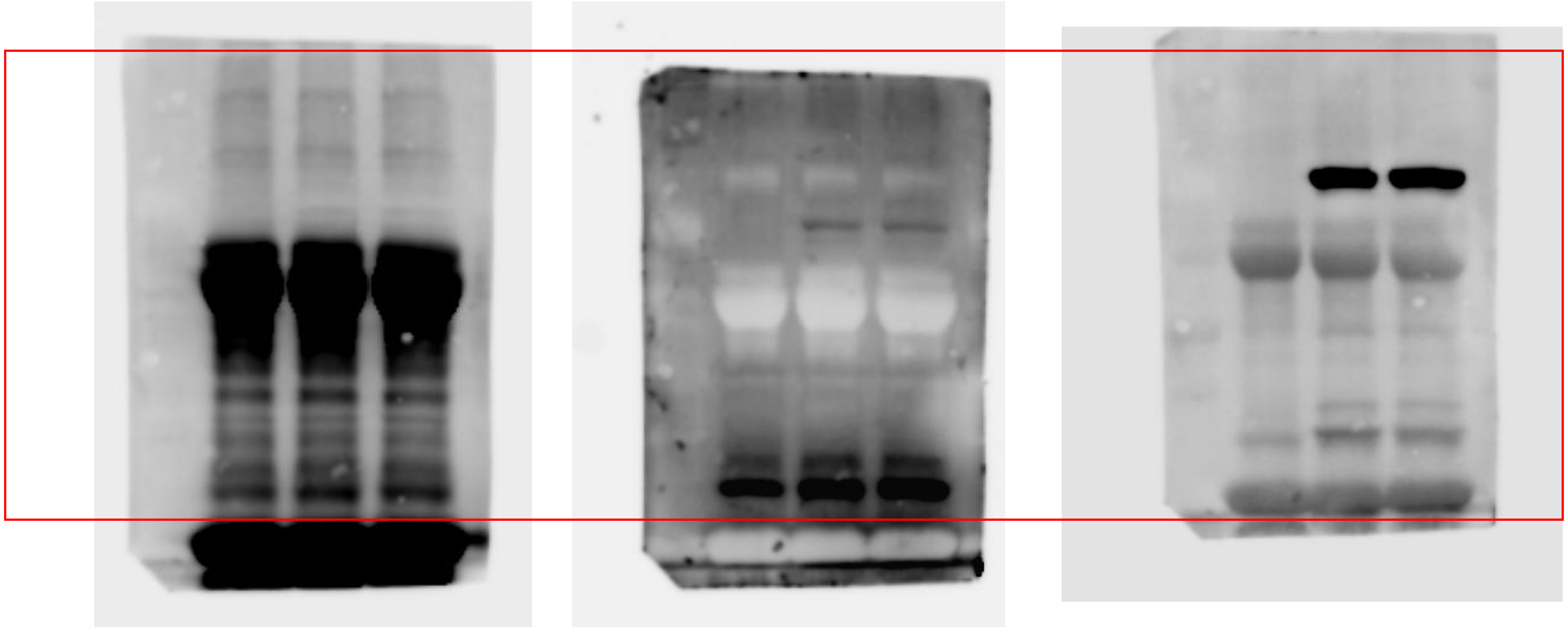

Fig.2A

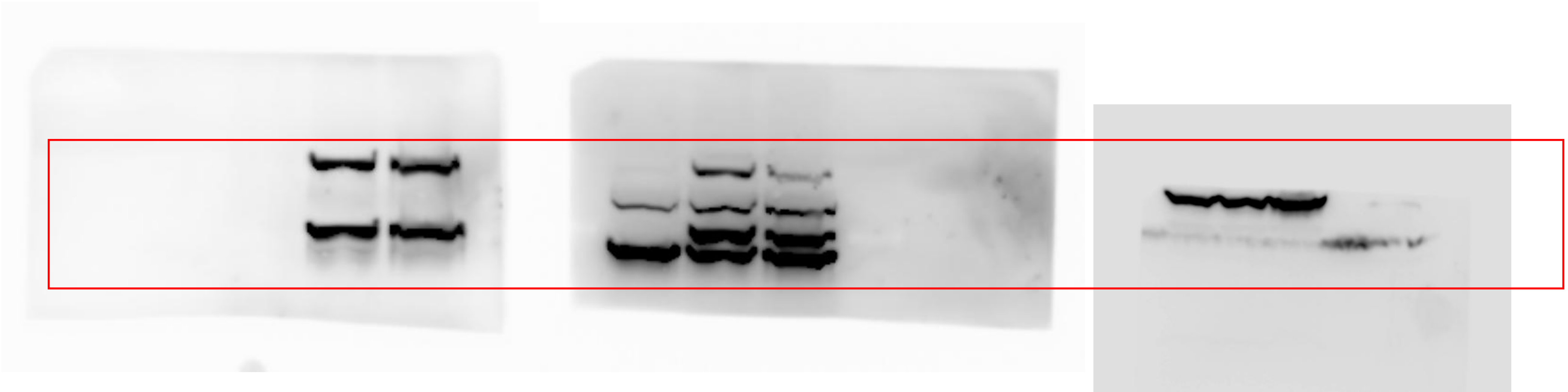

Fig.2B

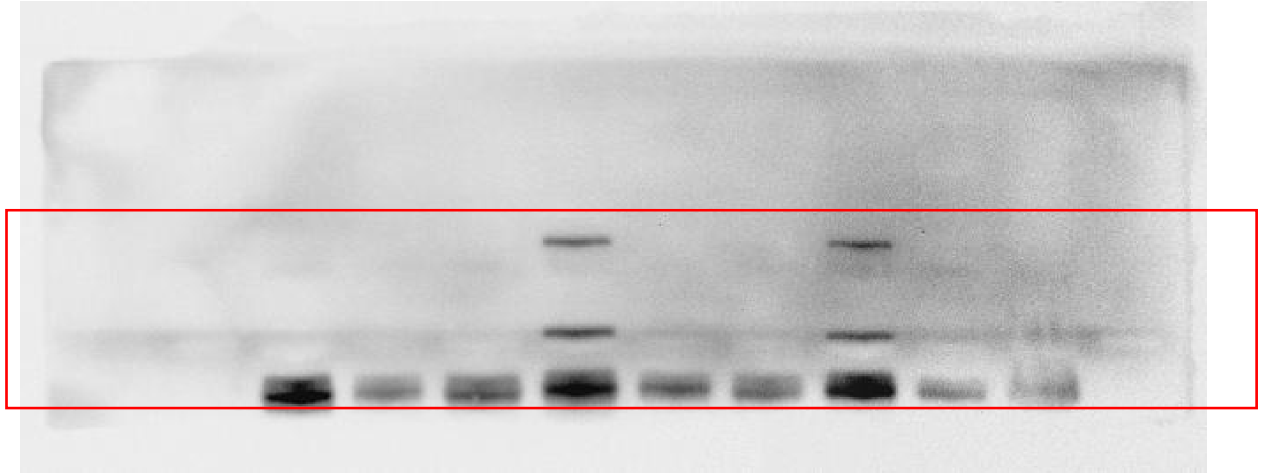

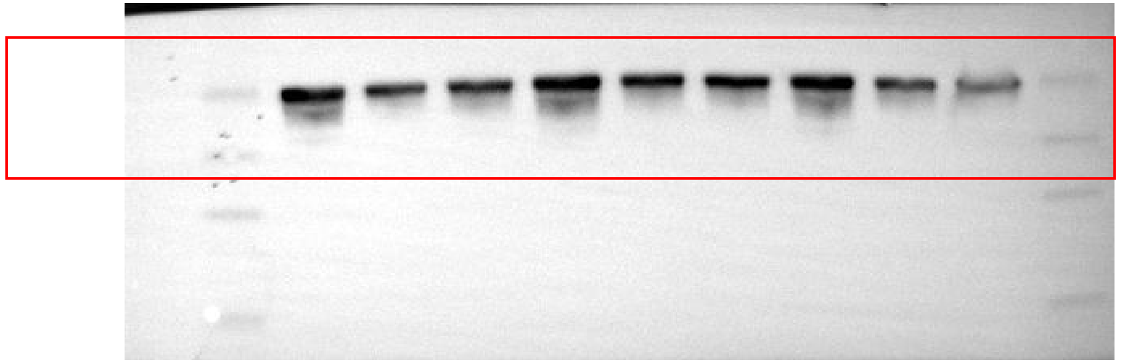

Fig.2I

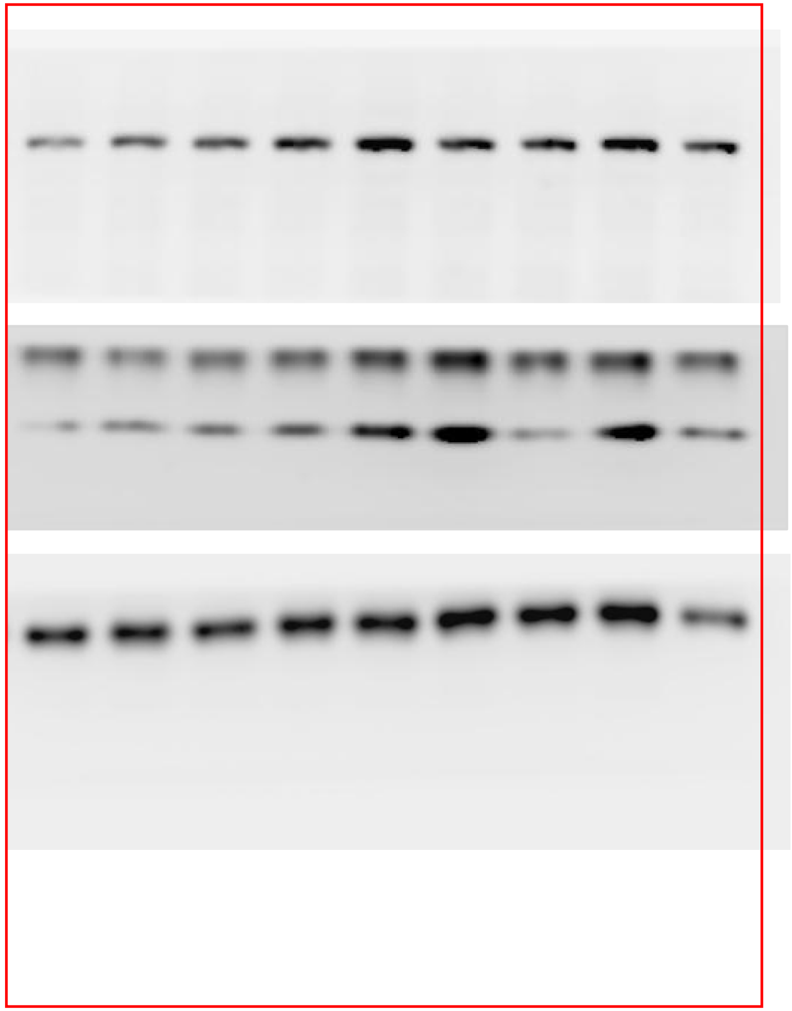

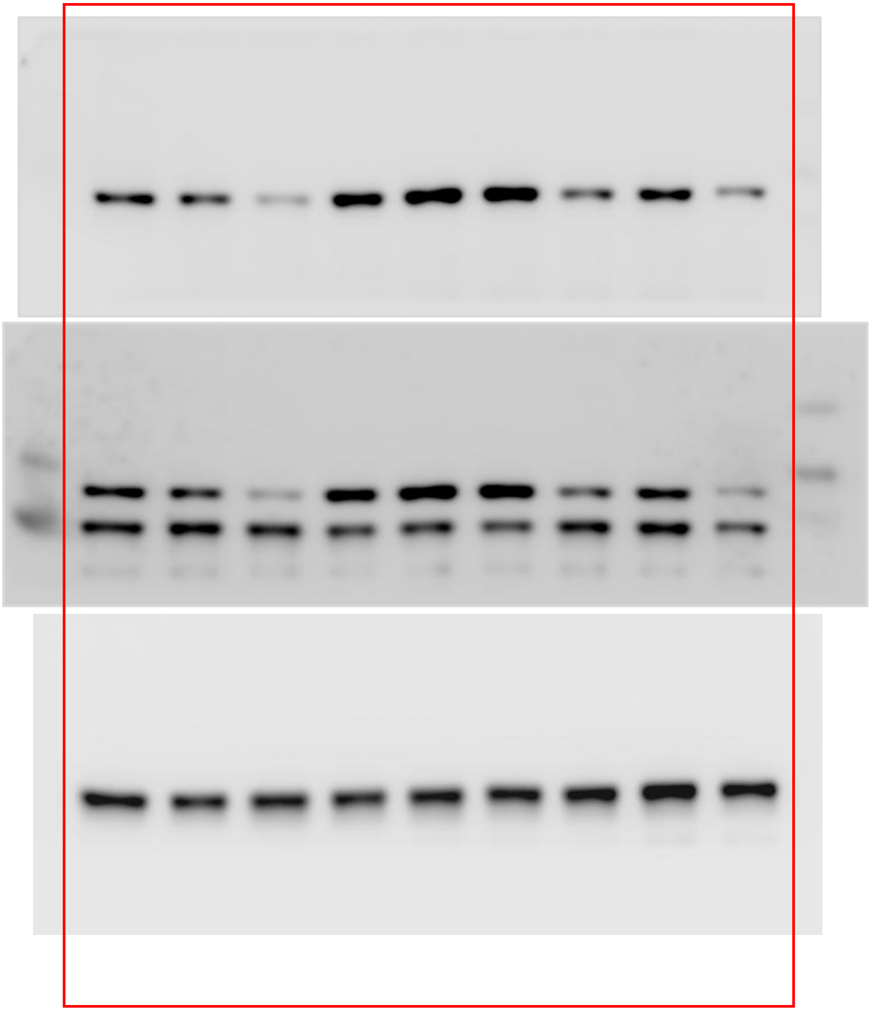

Fig.3F

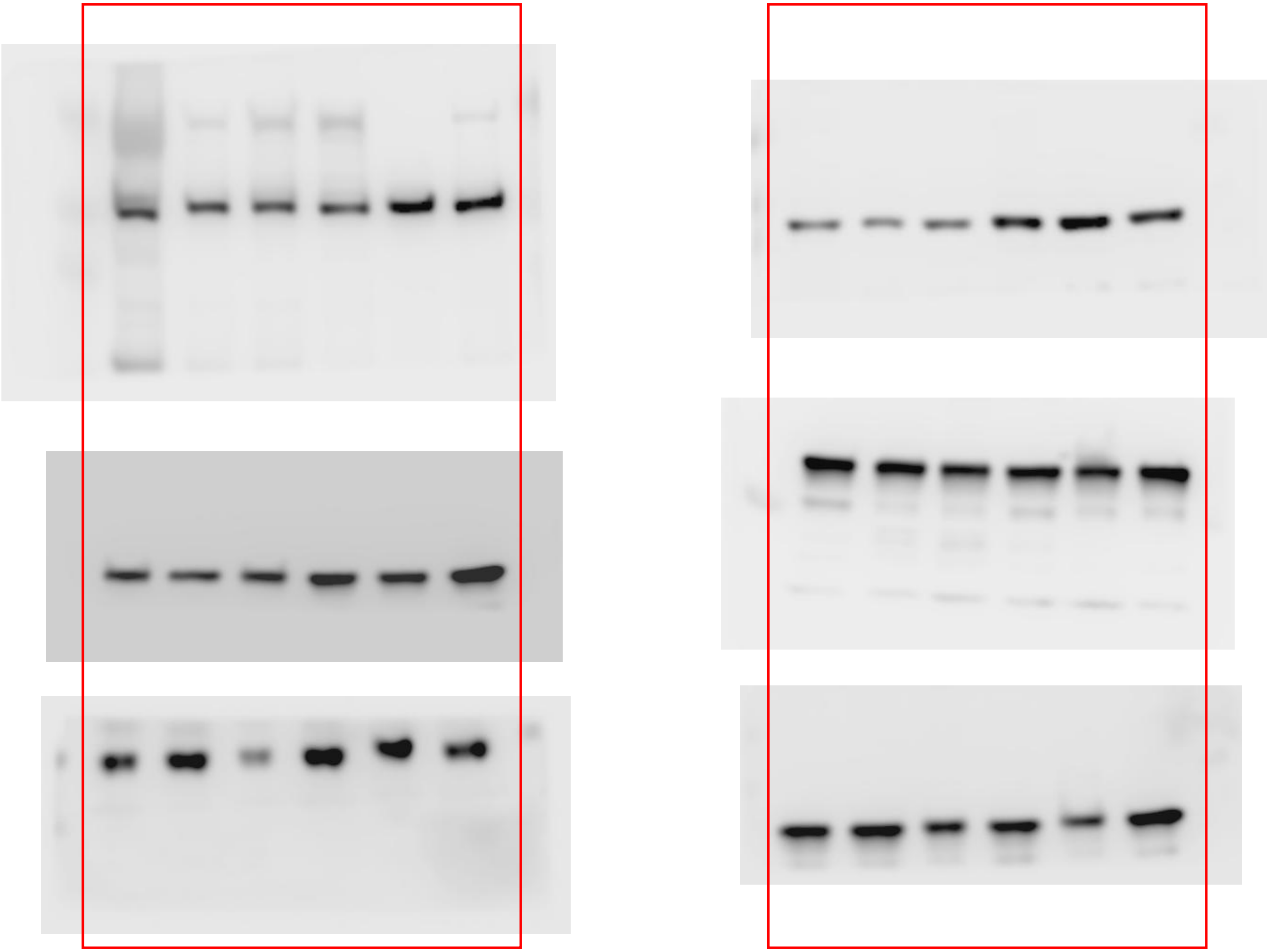

Fig.4A

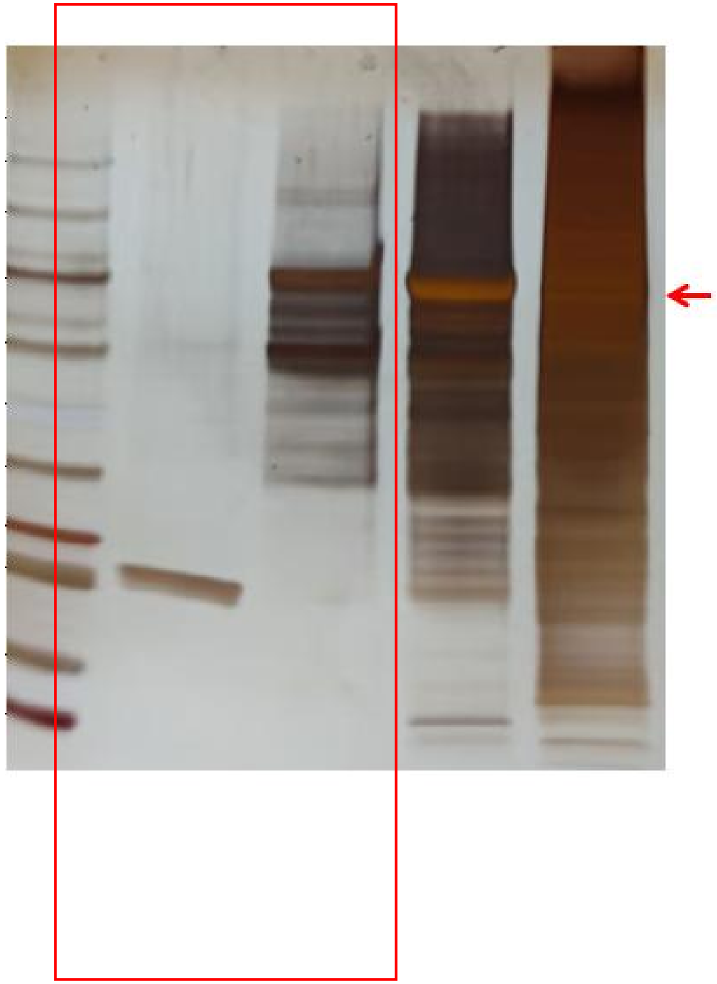

Fig.4F

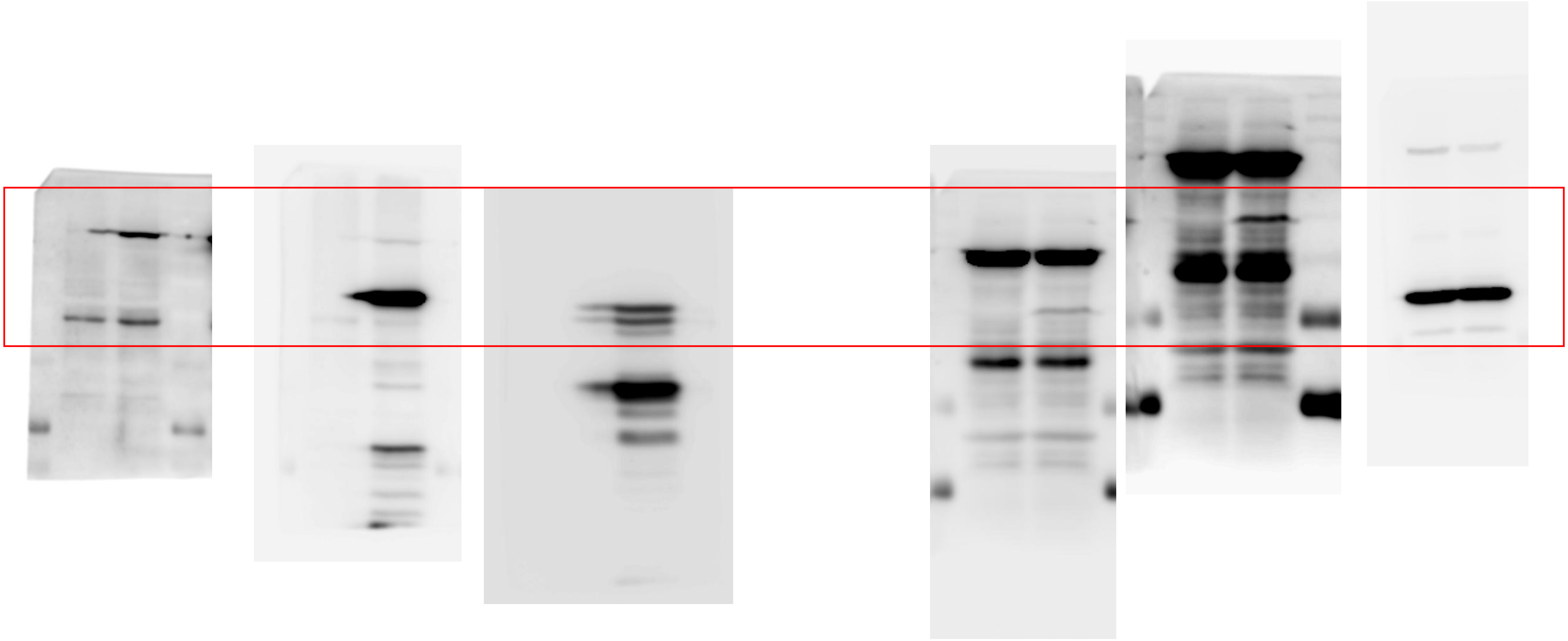

Fig.4G

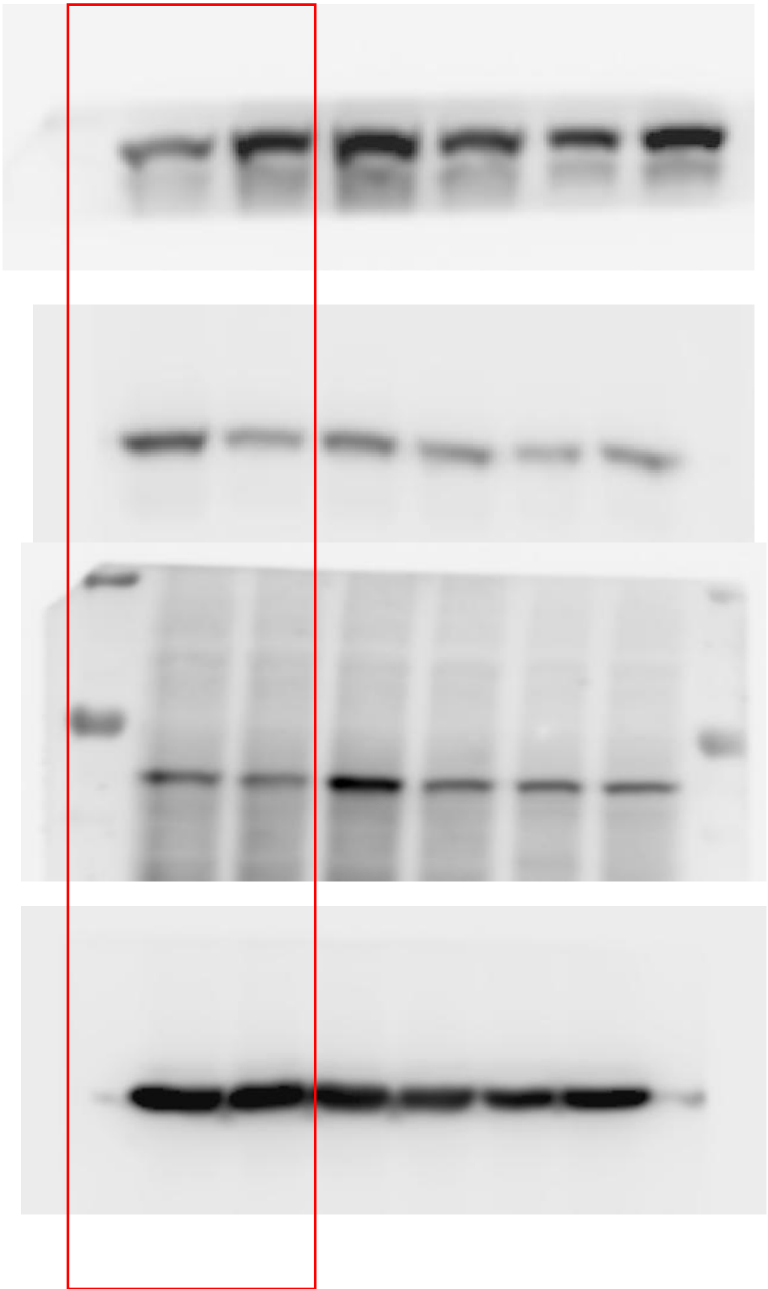

Fig.5H

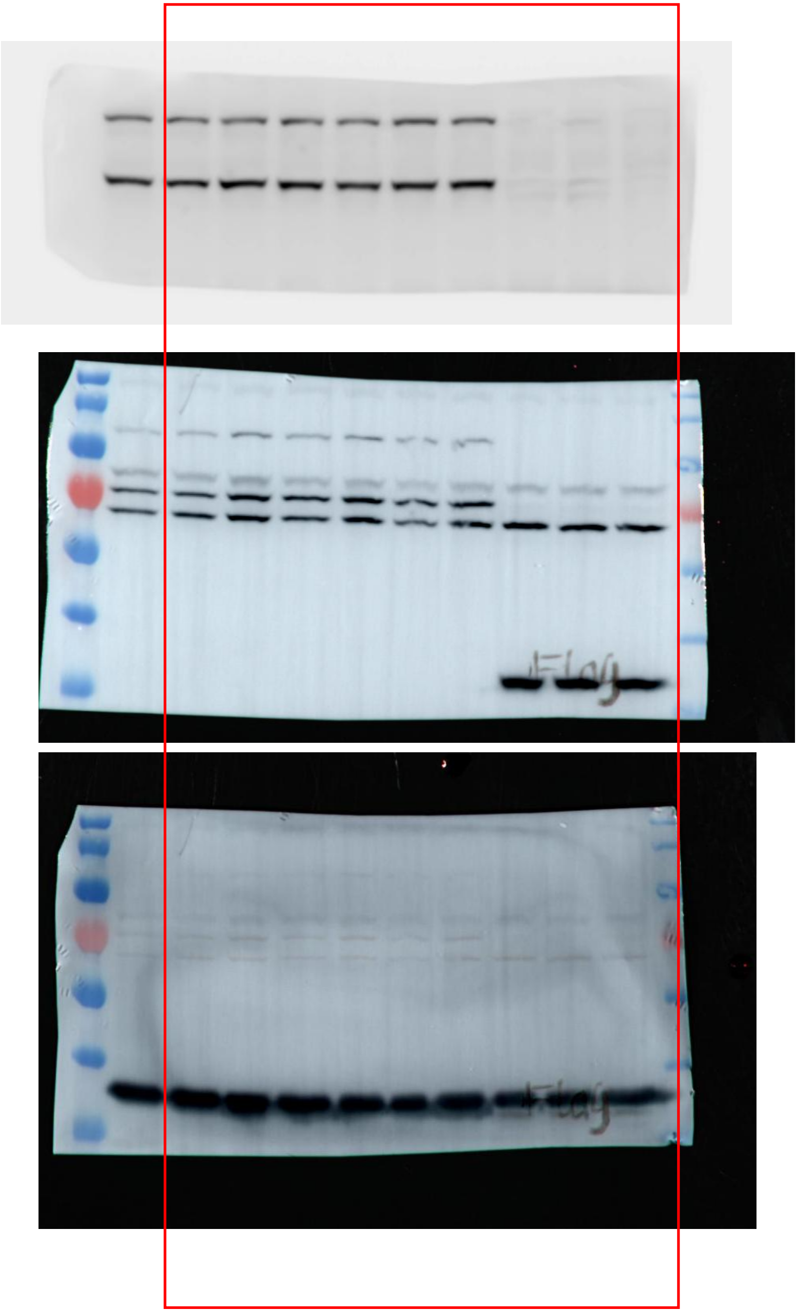

Fig.6B

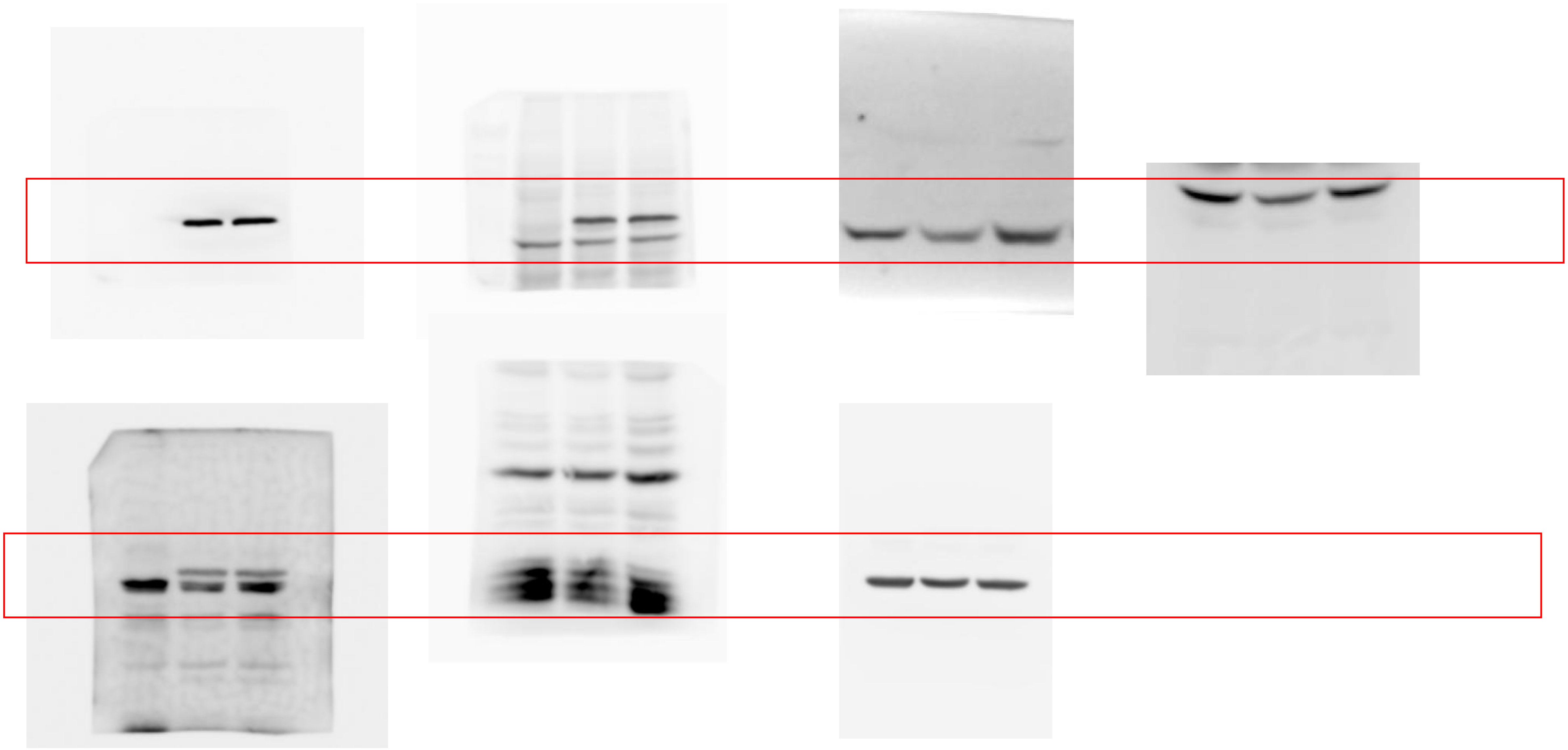

Fig.6C

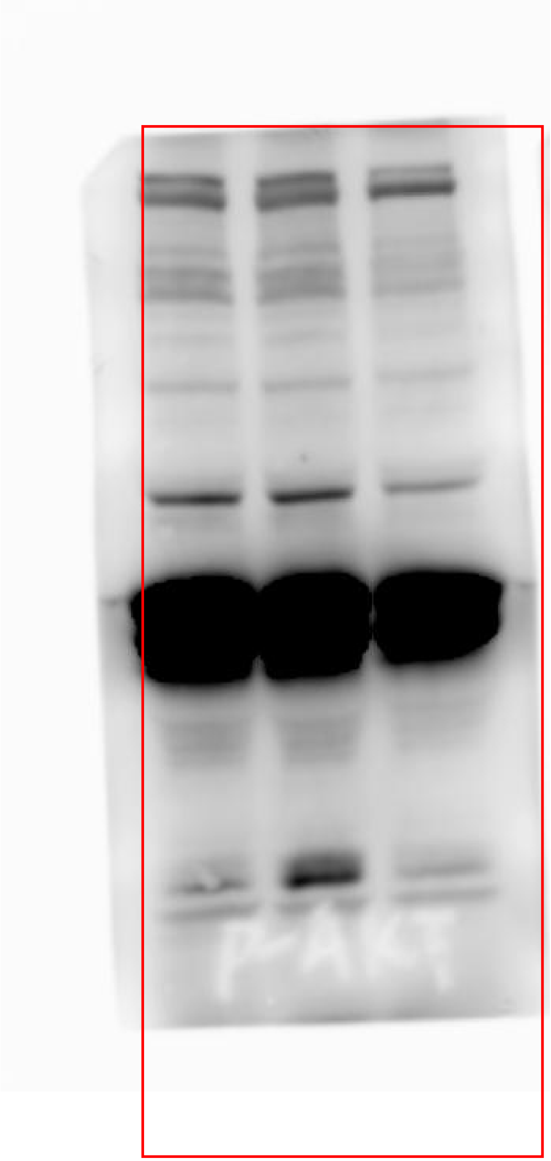

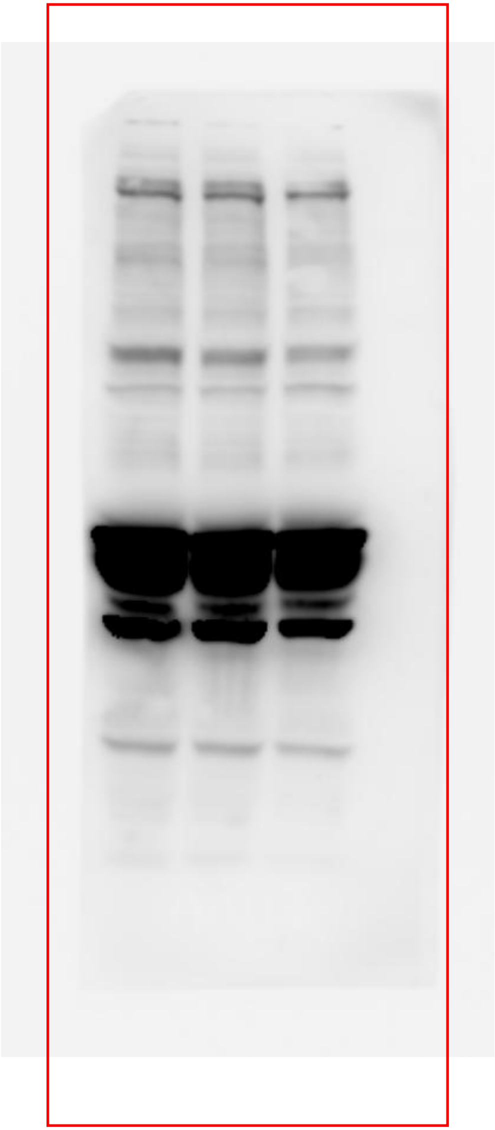

Fig.6D

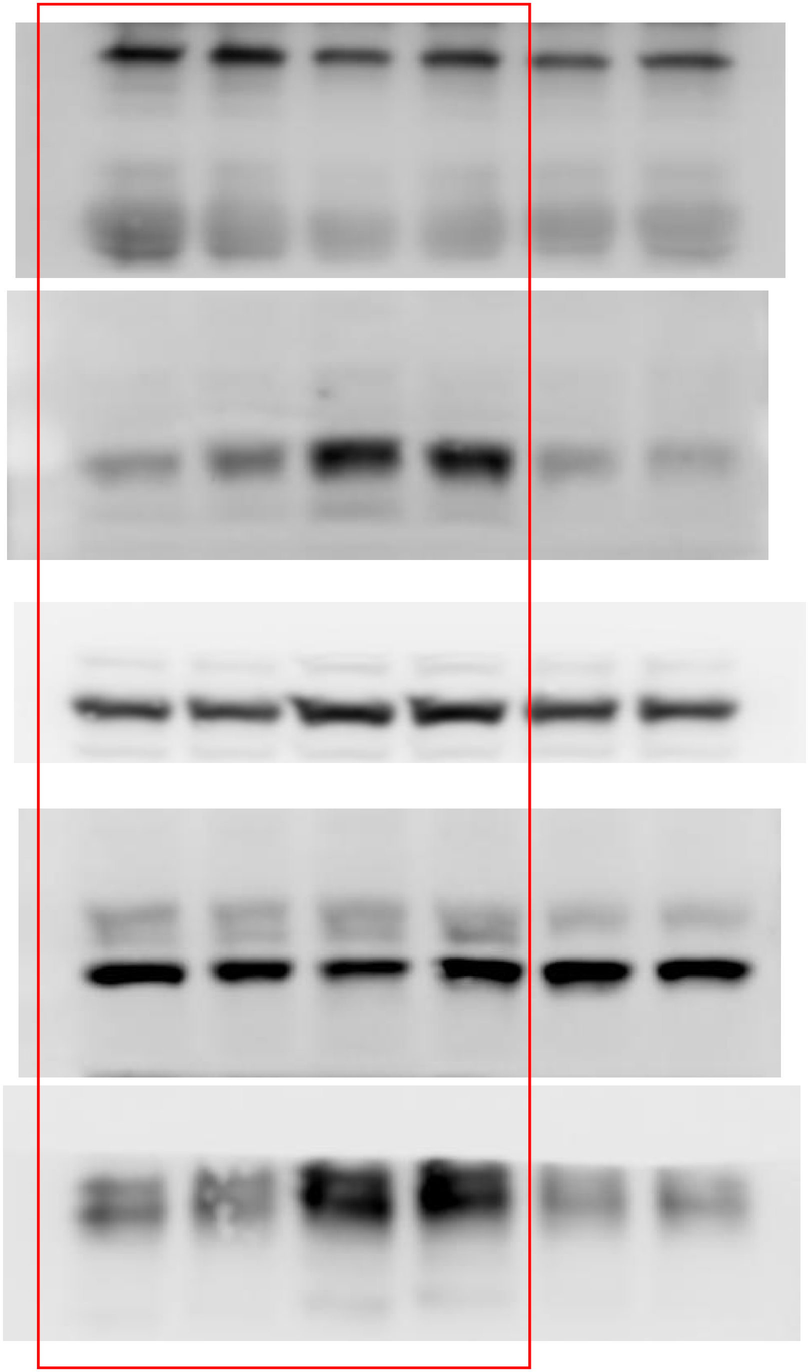

Fig.6F

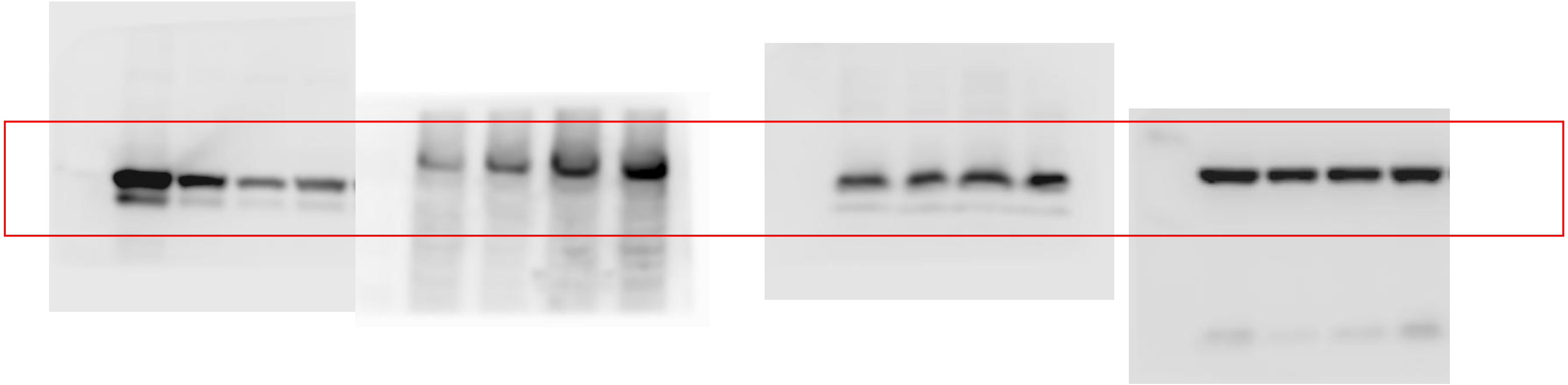

Fig.6G

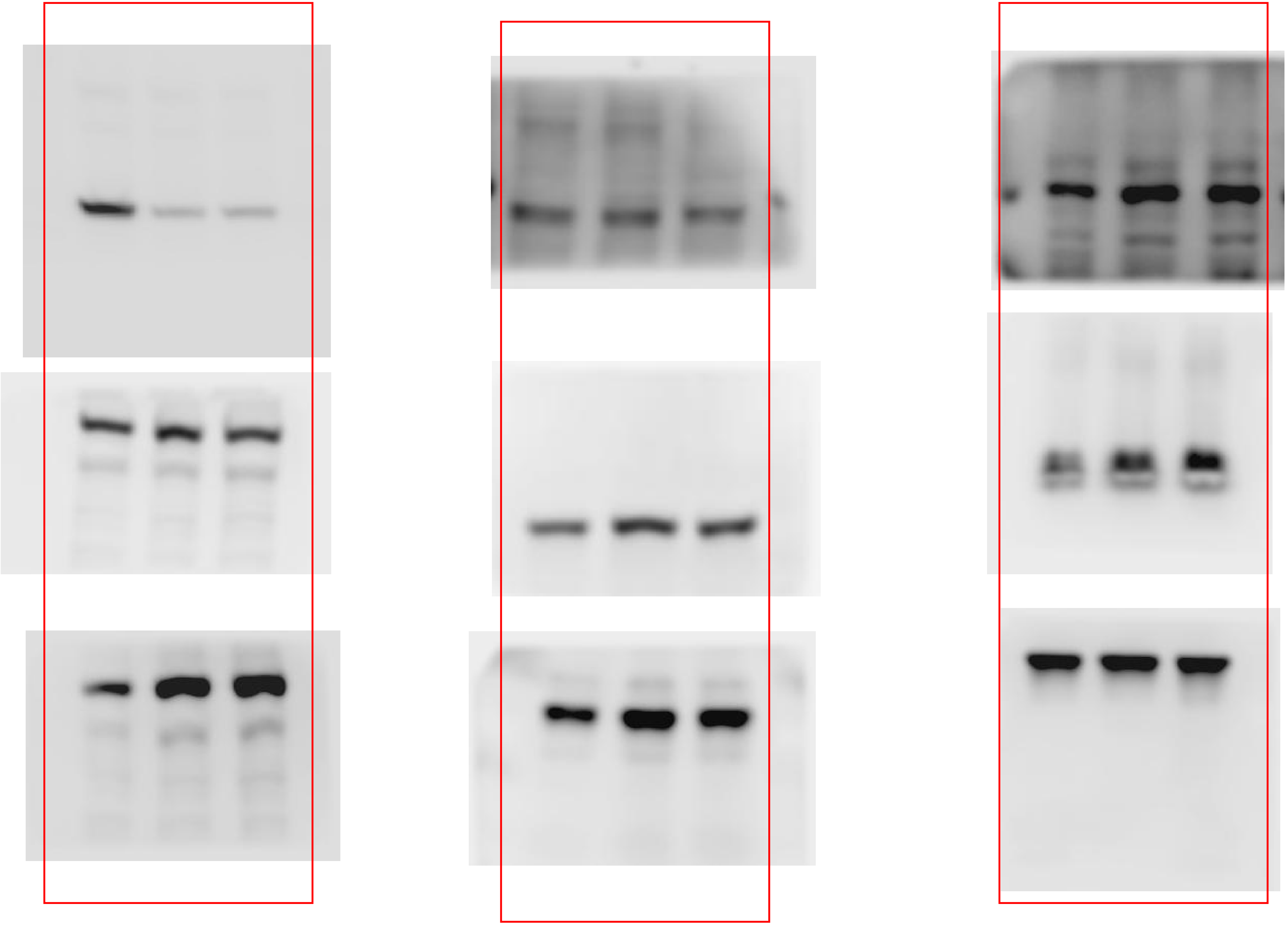

Fig.6H

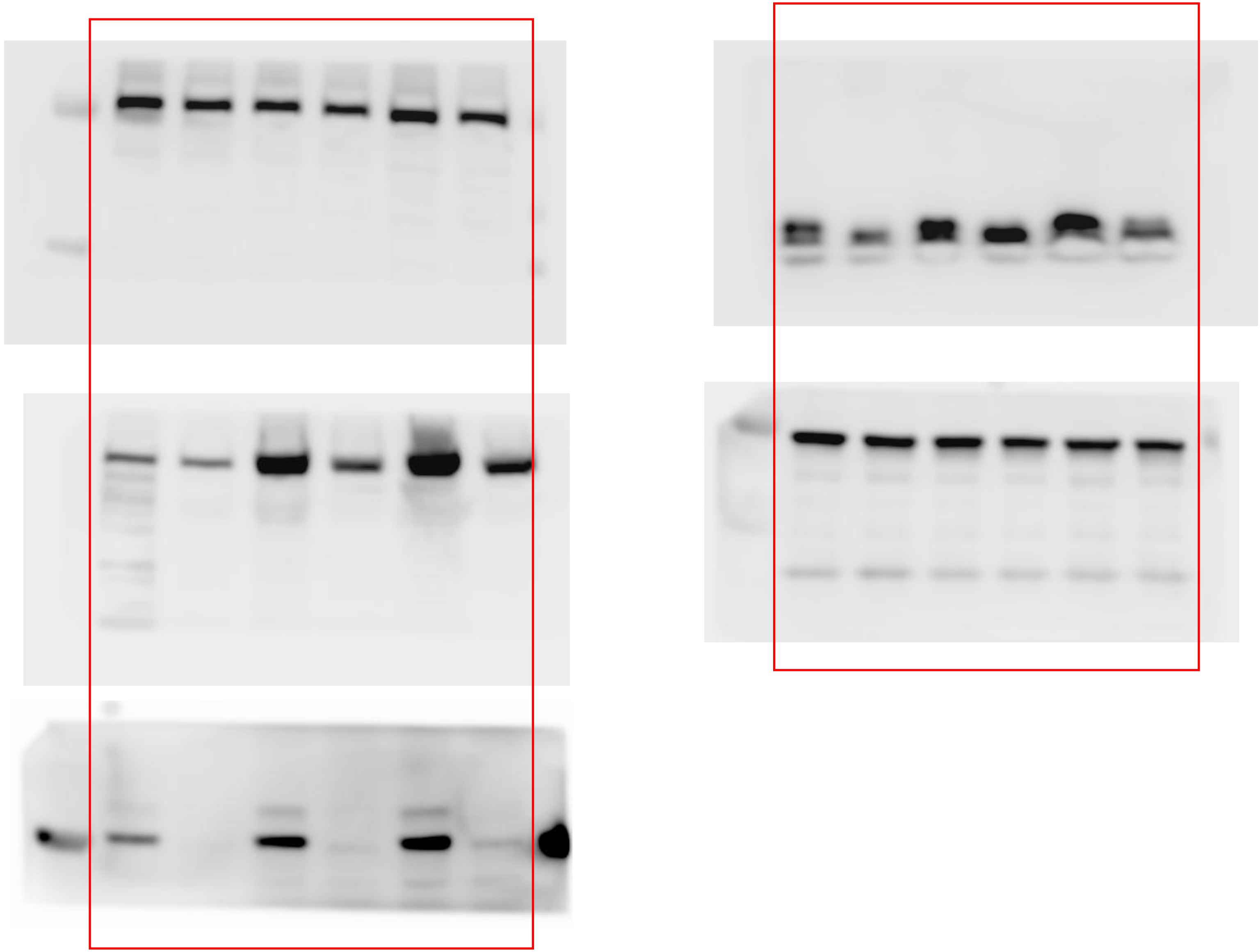

Fig.6J

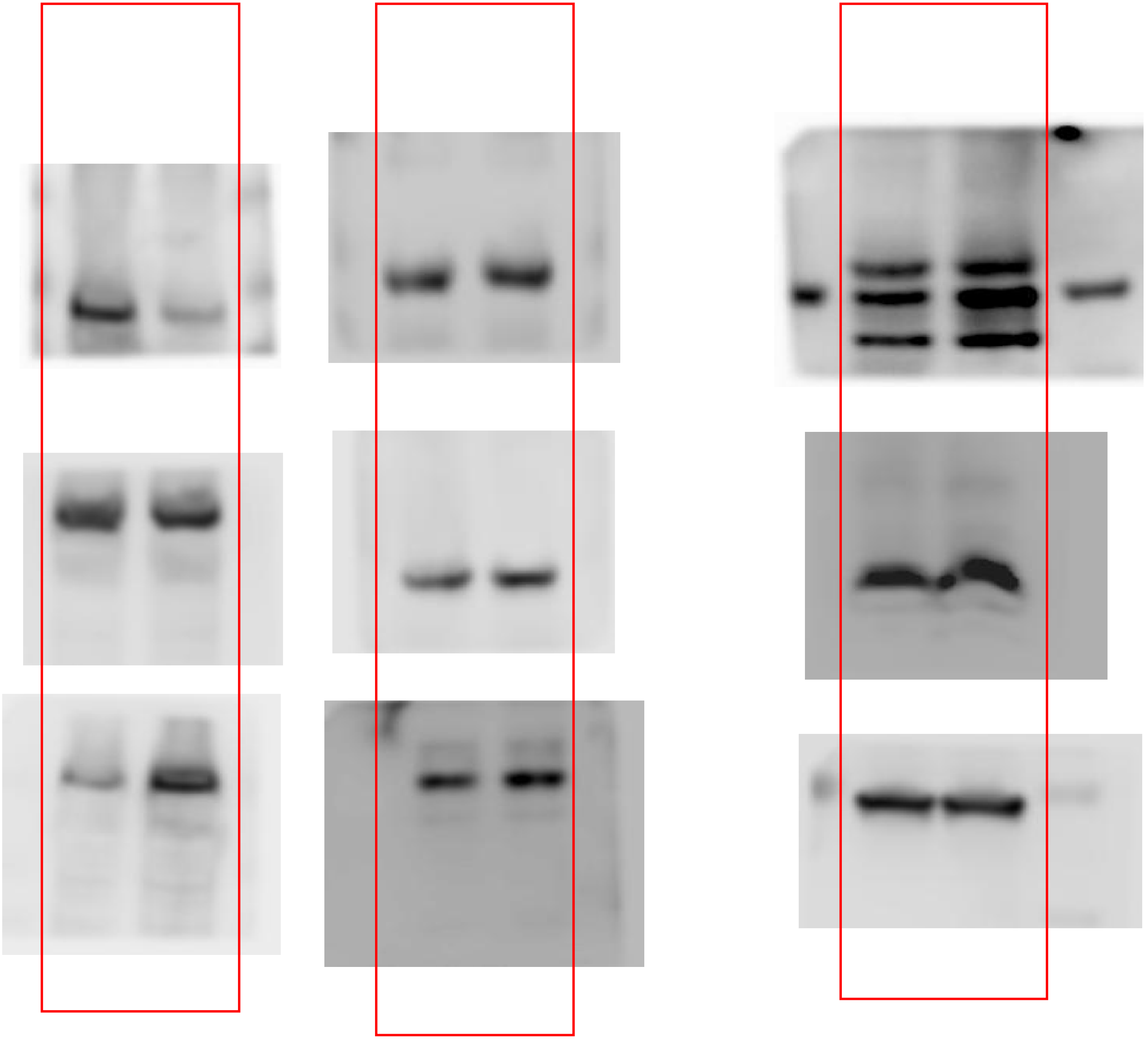

Fig.7E

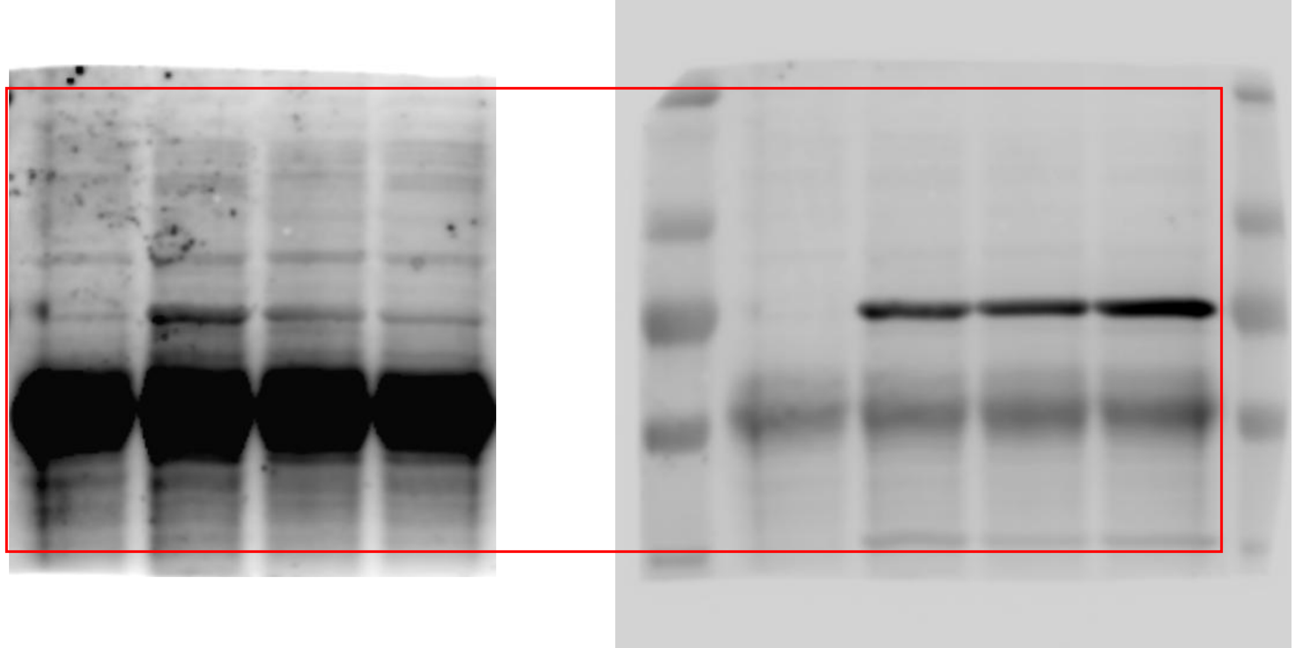

Fig.7F

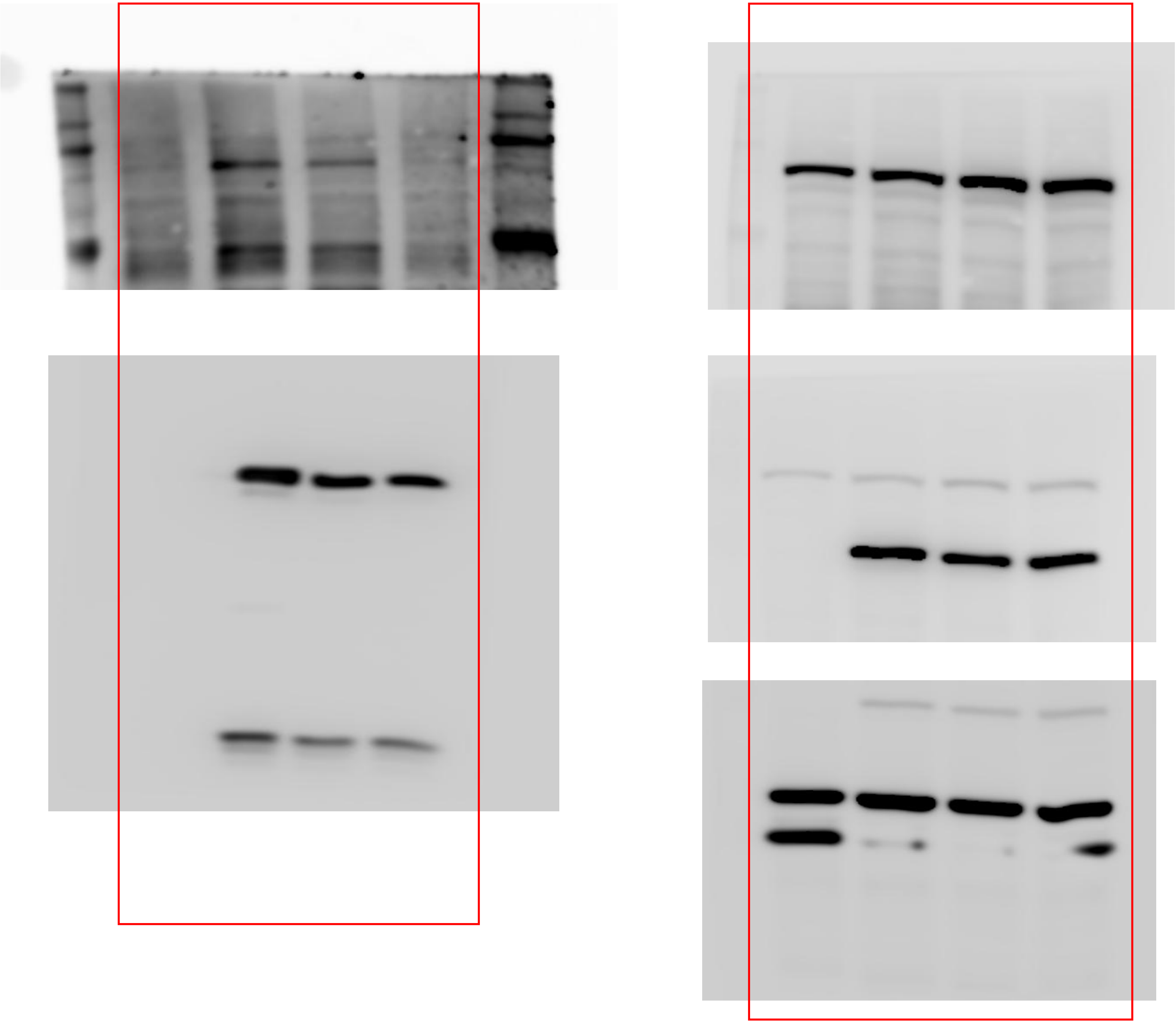

Fig.7G

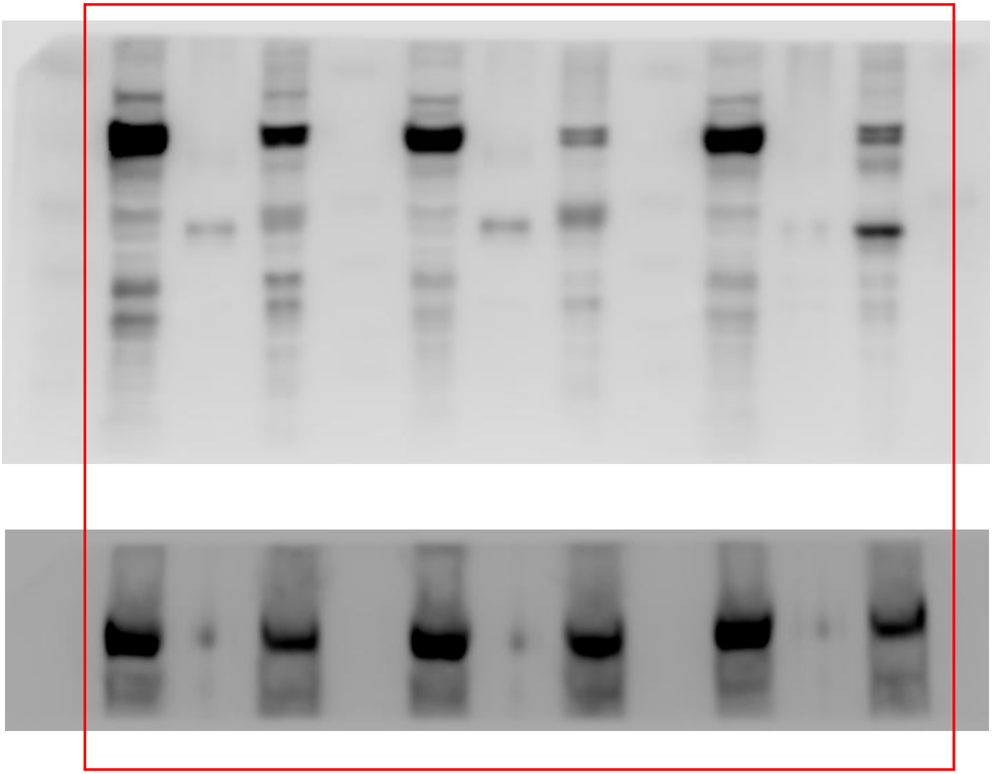

Fig.7H

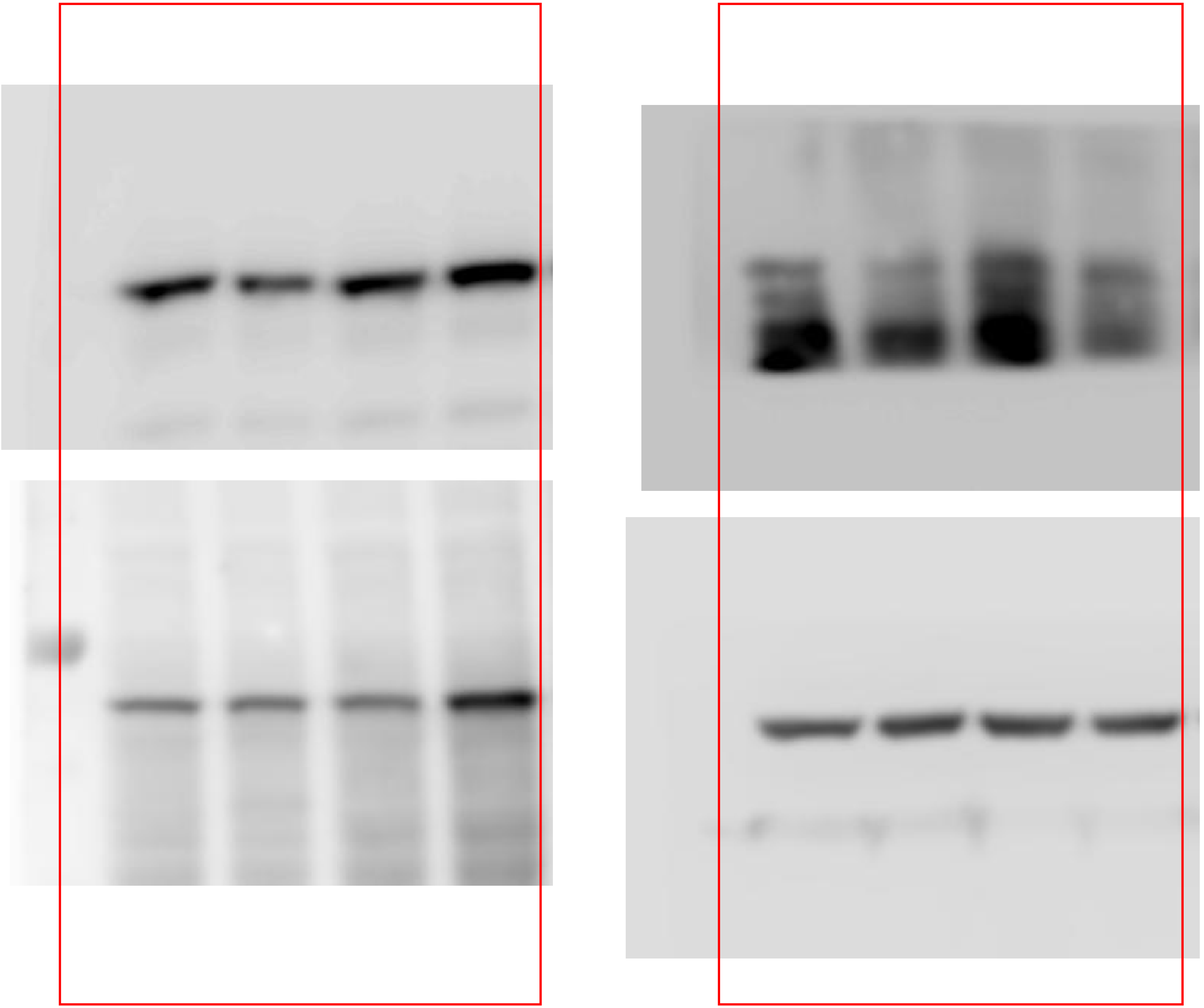

Supplement: Supplementary file 2 — Supporting File 2: advs75617‐sup‐0002‐Data_Raw WB gels.pdf. [file ADVS-13-e19856-s001.pdf]
